# Acceptance and commitment therapy for moral injury: conceptualizing and intervening on moral injury through the lens of contextual behavioral science

**DOI:** 10.3389/fpsyt.2026.1923699

**Published:** 2026-09-09

**Authors:** Lauren M. Borges, Jacob K. Farnsworth, Sean M. Barnes, Robyn D. Walser

**Affiliations:** 1 Rocky Mountain Mental Illness Research, Education and Clinical Center for Veteran Suicide Prevention, Aurora, CO, United States; 2 Department of Psychiatry, University of Colorado Anschutz Medical Campus, Aurora, CO, United States; 3 Rocky Mountain Regional Veterans Affairs Medical Center, Aurora, CO, United States; 4 National Center for PTSD Dissemination & Training Division at the Veterans Affairs (VA) Palo Alto Health Care System, Menlo Park, CA, United States

**Keywords:** acceptance and commitment therapy - ACT, contextual behavioral science (CBS), moral injury, psychosocial functioning, veterans

## Abstract

Moral injury is at a critical juncture in its conceptualization. Current dominant approaches to conceptualizing and assessing moral injury are rooted in syndromal frameworks with implicit medical model assumptions. In these approaches, painful moral emotions and cognitions are often conceptualized as pathological symptoms to be reduced or corrected. Such approaches risk pathologizing deeply human moral responses and may inadvertently position clinicians as arbiters of which moral appraisals or emotional reactions are appropriate, proportionate, or adaptive. In this paper, we present Contextual Behavioral Science (CBS), the philosophical foundation of Acceptance and Commitment Therapy for Moral Injury (ACT-MI), as an alternative framework for conceptualizing and treating moral injury without pathologizing moral pain or requiring clinicians to adjudicate the validity of clients’ moral judgments. Drawing on CBS, Relational Frame Theory (RFT), and evolutionary science, we examine the social functions of moral emotions, the human capacity to construct verbal moral narratives, and the implications of these processes for intervention. Building on these foundations, ACT-MI is described as an approach aimed at disrupting the behavioral patterns that maintain moral injury at both individual and group levels. Rather than targeting moral pain to reduce symptoms, using ACT-MI can facilitate transforming the individual’s relationship to it. Through functional assessment, clients’ efforts to avoid, control, or resolve moral pain are identified and addressed with core ACT processes, including acceptance, defusion, contact with the present moment, self-as-context, values, and committed action. Within this framework, individuals learn to hold moral pain while continuing to engage in values-based living and meaningful social functioning at individual and group levels of treatment. Assessment targets, methods, and hypotheses are identified to support testing the mechanisms and impacts of ACT-MI in future research.

## Introduction

Human beings are capable of profound moral anguish. When deeply held morals are violated, whether through one’s own or others’ behavior, the result is often long-lasting suffering. Over the last 35 years, the mental health field has increasingly recognized the long-lasting suffering brought on by moral violations as “moral injury” and sought to understand, assess, and treat it. Despite recognition that pain related to the violation of morals should not be “medicalized or pathologized” (1), we believe these laudable goals are hampered when syndromal models that implicitly draw on medical assumptions are used to conceptualize moral emotions and cognitions as symptoms to be eliminated. Such a framing risks obscuring what moral pain may signify: that deeply held morals have been violated and that the resulting anguish may be an understandable expression of our moral lives rather than evidence of disorder.

Moral injury interventions must be grounded in well-articulated theories that account for challenges in functioning, without pathologizing morality, implying that internal moral experiences are causal to suffering, or otherwise focusing on the need to repair moral pain. In keeping with this, we offer an alternative approach to conceptualizing moral injury, rooted in contextual behavioral science (CBS), and describe how its core theoretical assumptions facilitate conceptualizing even the most severe moral suffering without implicitly or explicitly pathologizing morality. Having laid this foundation, we present Acceptance and Commitment Therapy for Moral Injury (ACT-MI; 2–5), which offers a promising application of CBS principles for understanding and influencing the behavioral processes that contribute to ongoing moral suffering.

## Why morality matters

As social beings, morality guides and supports cooperation and survival as a species (6–8). Humans have survived not primarily as isolated individuals, but as intensely social organisms dependent on cooperation, shared norms, and coordinated group living (6–9). Both painful (e.g., guilt, shame, contempt; 10–13) and pleasant (e.g., awe, gratitude, pride; 14–16) moral emotions support human cooperation and survival by helping regulate social behavior in ways that maintain trust, cohesion, reciprocity, caregiving, and group stability (17, 18). These emotions, related thoughts, and associated urges help to reinforce the coordinated group living that has allowed humans to flourish. While pleasant moral emotions and cognitions tend to be desired and approached, painful moral emotions and cognitions are more likely to evoke efforts to avoid, escape, or reduce them. Thus, when a person experiences a moral violation, the painful emotions and thoughts that accompany it serve a function: motivating future behavior, typically in service of social values such as belonging, cooperation, community, and justice.

Moral emotions can motivate prosocial behavior (7, 10, 11, 14–16). One way to appreciate the functions of moral emotions is to reflect on common human experiences. Consider how guilt or shame may lead a person to recognize the harmful consequences of their actions on others. Conversely, emotions such as awe or gratitude can foster connection with the broader world and with meaningful relationships. Imagining the absence of these emotional capacities highlights their importance in maintaining social relationships and participation in communities.

Moral emotions play a critical role in guiding human behavior toward social connection, cooperation, and collective well-being. When these moral emotional signals are diminished, disregarded, or dismissed for competing contingencies, behavior may become increasingly organized around power, self-interest, or immediate reinforcement, sometimes with profound negative consequences for others ranging in severity from increased interpersonal struggles to large-scale violent societal conflict.

At the same time, inflexible responses to painful moral emotions and cognitions can contribute to increased suffering when attempts to avoid or control moral pain lead to further restriction in living (e.g., isolation and related relationship loss). Naturally, those suffering and their mental health providers are motivated to find treatment, but these efforts to help can inadvertently pathologize moral emotions central to our shared humanity.

## Medicalizing moral injury in psychology

Most models for assessing and intervening on moral injury that have emerged in the mental health field take a syndromal approach to conceptualization (19–22). Describing moral injury as a syndrome reflects the dominant culture of assessment and diagnosis in psychology, shaped by the Diagnostic and Statistical Manual (DSM; 23) and the International Classification of Diseases (ICD; 24). It has been argued that nosological systems like the DSM are in reality not atheoretical but instead reflect assumptions of a weakly articulated medical model (25) and imply latent disease classification (26). As defined in the DSM, a syndrome is “characterized by a clinically significant disturbance in an individual’s cognition, emotion regulation, or behavior that reflects *a dysfunction in the psychological, biological, or developmental processes underlying mental functioning*” (23, p. 14, emphasis added). This syndromal conceptualization, which focuses on symptom topography, emphasizes identifying the extent to which these symptoms are characteristic of a “harmful dysfunction” (27). Implicitly, then, this approach can frame distress as evidence that something within the individual is not functioning properly and therefore requires correction or repair.

Health systems and insurance companies anchored in the medical model have perpetuated the practice of measuring symptoms, classifying them as causal of functional impairment, and intervening to reduce them. Next, we describe how we believe that the medical model has been extended to the classification of moral injury. First, we briefly provide historical context for the identification of moral injury in psychology and then describe how some of the most prevalent approaches to conceptualizing and assessing moral injury may run the risk of pathologizing morality.

### The syndromal assessment of moral injury

The term “moral injury” emerged in the psychological literature in 1994, introduced by psychiatrist Jonathan Shay (28). Shay referred to moral injury as a “betrayal of what’s right” by someone who holds authority in a high-stakes situation. Although this definition laid the foundation for conceptualizing moral injury in psychology, it did not address the broader spectrum of moral violations humans may encounter. As Shay’s model and the existing PTSD diagnostic syndrome were not sufficient to characterize moral suffering, Litz and colleagues (2009) came forth with a more comprehensive definition for moral injury, referring to it as “the lasting psychological, biological, spiritual, behavioral, and social impact of perpetrating, failing to prevent, or bearing witness to acts that transgress deeply held moral beliefs and expectations” (19; p. 697). In Litz and colleagues’ conceptual model, moral injury is defined as a “psychological syndrome,” and the Moral Injury Outcome Scale (MIOS) operationalizes this syndromal conceptualization by assessing its proposed symptom manifestations (19; e.g., p. 3). Furthermore, moral emotions, beliefs, and behaviors are characterized as experiences that are “functionally impairing alterations” of this moral injury syndrome (29; p. 259).

In another syndromal model, moral injury is described similarly, likening it to a “stress-related syndrome” (30; p. 2) and Norman and colleagues developed the Moral Injury and Distress Scale (MIDS; 31) as a measure designed to assess “moral injury symptoms” (31, p. 1). In outlining their team’s goals for the MIDS development, Maguen and colleagues state, “identifying clinically meaningful moral injury goes beyond symptom detection and is the first step toward designation of a syndrome that is separate from other mental health conditions” (20, p. 687). These quotes establish that the two most prominent self-report measures of moral injury, the MIOS and MIDS, are explicitly based on syndromal frameworks. The goal of these syndromal conceptualizations is to assess clinically significant moral injury symptoms, including moral pain itself, and then track their reduction (and, by extension, the elimination of the underlying syndrome) in response to a course of treatment (19, 20, 29–31).

A syndromal framing of moral injury focused on decreasing moral distress is also at the core of many extant models of moral injury intervention (32–35), including treatments evaluated in randomized controlled trials (RCTs). These treatments include Adaptive Disclosure where letter writing to a harmed person to repair the harm is central to intervention (33); Impact of Killing that targets identifying and challenging maladaptive killing-related cognitions (32); Trauma Informed Guilt Reduction that emphasizes guilt appraisal (35); and Building Spiritual Strength that focuses on resolving moral distress through the empty chair technique to facilitate forgiveness from a higher power (34). Across these interventions, treatment aims tend to include identifying, challenging, and alleviating painful moral emotions and cognitions. Defining treatment success in terms of reductions in moral injury symptoms implicitly frames painful moral emotions and cognitions as indicators of an underlying dysfunction and positions their alleviation as a primary therapeutic goal. While moral injury is not currently classified as a psychological disorder in the DSM (currently a Z code in the DSM-5-TR [23] under the category “Moral, Religious, or Spiritual Problem”), some authors have advocated for the inclusion of moral injury as a new psychiatric diagnosis (36–38).

### Scientific assumptions of a syndromal approach

Using a syndromal framework to understand moral injury is a conceptual choice (not a necessity). Rather, it reflects scientific assumptions that both shape and constrain how moral injury is identified, assessed, and addressed (39). To further expand on the limitations implicit in a syndromal approach to moral injury and facilitate direct comparison with a CBS model, we will first identify the scientific assumptions underlying these frameworks regarding ontology, the unit of analysis for assessment and intervention, epistemology, and truth criteria. We then illustrate how these differing assumptions shape the conceptualization, assessment, and treatment of the same clinical case involving moral injury.

In a syndromal framework, an *ontological approach* to understanding moral injury is adopted, where moral injury is conceptualized as an underlying condition or syndrome inferred from the presence of particular thoughts, emotions, and behaviors. This ontological stance treats moral injury as an entity that can be identified, measured, and targeted for intervention. Consequently, clinical attention is directed toward the syndrome, and the *unit of analysis* is the symptom—the abnormal behavior (40). Thus, through a syndromal lens, the form of the symptom becomes the primary focus of evaluation and intervention, with painful thoughts, emotions, and behaviors conceptualized as manifestations of an underlying disorder that explains suffering. As previously noted, treatment success is typically defined by clinically meaningful symptom reduction or remission of the disorder. Within this framework, moral injury can be represented categorically (presence or absence of the symptom) or dimensionally (severity across a continuum). The syndromal framework’s *epistemological assumptions* position the clinician as an objective, independent observer who is outside of, and therefore separate from, the moral injury phenomenon being assessed. In this way, the clinician assumes a role analogous to that of a physician evaluating whether a patient’s signs and symptoms correspond to a particular disease process. Finally, the framework relies on a *truth criterion*, whereby the accuracy of a clinical account is judged by the extent to which an individual’s reported symptoms correspond with the predefined features of the moral injury syndrome. Clinical interviews and standardized self-report measures provide the means to evaluate this correspondence and determine the presence or severity of the features associated with the syndrome.

Not all providers using symptom measures to classify moral injury will explicitly believe that moral pain is in and of itself pathological. However, the implicit assumptions of syndromal frameworks for moral injury, where intervention outcomes focus on symptom reduction, may still inadvertently position clinicians as epistemic authorities regarding what constitutes healthy, adaptive, or appropriate moral responding. As noted earlier, defining a specific list of moral experiences as a syndrome necessarily conveys that those symptoms reflect dysfunction. However, because there is no empirical method to validate the accuracy of moral statements or experiences (41), asking providers to adopt intervention models that frame a client’s moral thoughts and emotions in this way might implicitly position them as moral authorities in the therapeutic relationship. In addition to being difficult to justify empirically, such a dynamic may also risk invalidating the client’s moral distress if they feel it is morally appropriate.

For example, a client who deeply values protecting vulnerable people may experience moral distress after witnessing the sexual assault of a vulnerable person and not intervening. Following this experience they may feel that their moral distress rightfully results from the moral violation. In such cases, clients may experience a syndromal approach to assessment and intervention (focused on reducing moral pain related to this event) as minimizing or misunderstanding the meaning and function of their moral suffering. Conceptualizing distress as the primary target of intervention therefore may risk pathologizing what may be an understandable and functionally coherent moral response. When providers have not experienced their client’s moral violations and do not share the same social values, an approach focused primarily on symptom reduction may not align with the client’s expectations of treatment, potentially contributing to disengagement or early termination.

Through the following scenario we illustrate this ontological, syndromal-based approach to conceptualizing and intervening on moral injury. A veteran completes a measure of moral injury and meets criteria for the syndrome. When asked about their morally injurious event, they explain that they killed a child in the context of returning fire after an enemy combatant positioned themselves behind women and children, placing the veteran’s unit in immediate danger. They express that they did not intend to harm the child and on the moral injury measure identify that they strongly agree with statements related to shame, such as “I blame myself.” Although the veteran reports that they acted to protect fellow service members, the event still violated their moral principles regarding protecting children. In the context of a syndromal conceptualization of moral injury, this veteran would be considered to have moral injury *(ontological)* due to elevated scores on the endorsed symptoms such as “I blame myself” *(unit of analysis).* The assessor, using putatively objective methods, recognizes the veteran’s moral injury symptoms *(epistemological)*. The accuracy of the resulting clinical formulation is evaluated according to how well the veteran’s reported experiences correspond to the predefined features of the moral injury syndrome (*truth criterion*). From this perspective, grounded in assumptions about objective moral correctness, particular treatments may then focus on helping the client determine whether the belief “I blame myself” and associated thoughts are rational, distorted, or inaccurate. If it is held that the veteran made the correct tactical decision under the circumstances, these assumptions may logically point to interventions like challenging the thought “I blame myself,” restructuring associated cognitions, or reducing shame-related distress. The priority here, is targeting the symptom topography viewed as harmful and reducing symptoms like “I blame myself” which are indicative of the moral injury syndrome and thus become objects of correction, rather than potentially useful moral responses.

## Applying a contextual behavioral philosophy of science to moral injury

### Defining moral injury as a pattern of behavior

The principles of CBS can serve as an alternative framework for addressing moral injury. As a philosophy of science, CBS supports a conceptual model of moral injury with clear theoretical assumptions rooted in evolutionary science and Relational Frame Theory (RFT; 44). The theoretical and scientific assumptions of CBS (40) provide a framework for viewing moral injury not as a psychological syndrome but as a pattern of behavior. Potentially morally injurious events (PMIEs; e.g., the aftermath of killing, betrayal, abuse, abandonment, systemic injustice, failure to protect others, or other moments in which individuals feel they have otherwise violated what matters most to them) are understood similarly across CBS and syndromal models as situations representing moral violations. However, painful moral emotions, cognitions, urges, and sensations are conceptualized differently.

In contrast to a syndromal medical model that treats moral distress as symptoms representing underlying pathology, moral injury is defined in a CBS model by one’s behavior in response to painful moral emotions and cognitions (i.e., “moral pain”) (4, 42). That is, moral injury is not the presence of moral pain or a certain amount of pain, but it is defined by the specific behaviors used to avoid, control, or repair morally painful experiences in ways that increasingly constrict values-based living and social connection (4, 42). In a CBS model, moral pain does not become moral injury once it reaches a specific threshold of intensity, as an individual could experience that pain and still live in a way that is consistent with their values. Rather, moral injury results when an individual’s pattern of relating to their moral pain adds to their own and others social, psychological, or spiritual suffering. Because suffering is a subjective experience determined by each individual, moral injury results not from the presence or absence of a certain behavior, but *how* an individual interacts with their moral pain. The client’s efforts to avoid and control moral pain are targets of assessment and treatment, rather than the pain itself, and the outcome of interest is improvement in functioning through psychological flexibility rather than symptom reduction (4, 42). Therefore, applying CBS principles to moral injury-related suffering results in a different assessment and treatment approach based on theoretical assumptions that are fundamentally different from syndromal approaches. Next, we review the scientific assumptions and targets of a CBS approach. ACT-MI is then presented as an intervention framework with assessment practices and testable hypotheses identified for future research.

### An a-ontological approach

Unlike syndromal approaches, where the primary unit of analysis is the symptom cluster presumed to reflect an underlying disorder, within CBS a monistic, functional perspective is adopted in which thoughts, emotions, sensations, and overt actions are understood as forms of behavior occurring in context. From this perspective, internal and external events do not require separate explanatory systems and can therefore be analyzed using the same functional principles. At the ontological level, CBS adopts a pragmatic *a-ontological* stance, focusing on the prediction and influence of behavior rather than on determining the ultimate nature of reality. This perspective is compatible with monism where mental and physical events are understood as part of a unified reality rather than separate substances; the mind and body constantly interact, so they cannot be meaningfully separated. External physical activities and internal mental activities are all viewed as behaviors that can be subjected to functional analysis (see ACT-MI section). Monism, therefore, gives CBS-oriented clinicians the conceptual freedom to directly intervene on any set of behaviors regardless of their location or topography.

### The act-in-context (the unit of analysis)

Rather than focusing on symptoms (a syndromal, medical model’s unit of analysis), in CBS the unit of analysis for assessment and treatment is the *act-in-context* (40). The act-in-context is a holistic approach in which any activity or event is inseparable from the historical and situational contexts in which it emerges. Clinically, this means that rather than conceptualizing intervention as the identification and reduction of unwanted symptoms, the CBS clinician seeks to understand the functions that a behavior serves for the individual, focusing on what the behavior accomplishes in context rather than on its form (e.g., the specific thought, emotion, or symptom).

CBS clinicians begin by identifying functional processes that may be maintaining suffering (e.g., avoidance and control-related processes) and examine how emotions, thoughts, and behaviors participate in those processes within an individual’s context. For example, rather than targeting the content of the thought “I blame myself,” a CBS clinician would examine what the client does when the thought arises, the contexts in which these responses occur, and their consequences for the client’s functioning. When internal experiences are conceptualized functionally rather than syndromally, thoughts and emotions are no longer viewed as symptoms requiring corrections, but as contextual events that influence the client’s ongoing patterns of responding.

Assessing moral injury from a CBS perspective, then, is not about identifying the symptom but about examining how individuals interact with both their covert internal experiences (e.g., thoughts, memories, emotions, and urges) and their overt environmental conditions (e.g., interpersonal and situational contexts). Internal experiences are conceptualized as contextual events that participate in an ongoing stream of human responding (40). An important implication of this perspective is that behavior cannot be meaningfully reduced to isolated symptoms or singular causal processes. Instead, behavior is organized into dynamic, relational networks, or relational frames, that continually and mutually influence behavior (see the RFT section for a description). Consequently, targeting the form of any singular thought or emotion by attempting to change its content may prove ineffective when the broader contextual and relational systems that maintain suffering remain unchanged. Instead, in CBS, the focus is on changing an individual’s relationship to these experiences in the *contexts* in which they arise (2, 42).

### Evolutionary epistemology

CBS is rooted in an evolutionary theory of knowing, in which knowledge develops through processes of variation, selection, and retention operating within context at individual and group levels to facilitate survival (43). At the level of group survival, behaviors that effectively help individuals immediately navigate their environments, reduce threats, obtain resources, maintain social connections, or respond to challenges are more likely to persist over time (43). By focusing on the purposes moral pain serves for the individual (and species) rather than attempting to reduce these experiences, the importance of that pain is honored, and the contexts that gave rise to the individual’s experiences are validated.

Within CBS, humans are contextually situated beings with their own histories and circumstances. It is therefore critical to consider how these histories and contexts shape the therapeutic relationship; the context in which moral injury is assessed and addressed. Within CBS, a goal of assessment is to understand how a client’s idiographic history and current context shape and maintain patterns of responding associated with moral injury-related suffering. These same processes apply to clinicians; accordingly, clinicians’ learning histories and ongoing verbal behavior, including private events, are understood to influence the therapeutic process continuously. A clinician’s contexts (their memories, emotions, and beliefs) continuously shape their stream of behaviors and their responses to their client (44).

Thus, it is impossible for any human to fully escape their own verbal behavior or completely transcend their own subjective learning history. This observation applies equally to clients and clinicians, precluding either party from occupying a wholly objective position from which to answer moral questions in therapy. Given this recognition, the CBS clinician chooses not to serve as a moral arbiter, determining whether a client’s actions, emotions, or judgments are morally correct or incorrect. Nor does this stance imply moral relativism; rather, it recognizes that moral judgments themselves occur within historical, relational, cultural, and situational contexts. Instead of presuming to know what is moral, CBS clinicians enter therapeutic relationships as fellow humans with their own morals, values, histories, and experiences, and, from that stance, work collaboratively with clients to understand how their behaviors may be maintaining suffering in their environments, in service of the client considering behavioral change.

### Pragmatic truth criterion

A CBS framework requires clinicians to ask a different question about truth. Instead of defining the truth about the presence of moral injury by meeting a threshold of symptoms, an individual’s behavior is examined in terms of how it functions in their life. In other words, truth is evaluated based on its utility to the individual (45). Applied to moral injury intervention, the question, for example, is not whether a morally painful thought is “true” in an absolute sense, but rather whether the individual’s way of responding to that thought effectively supports the kind of life they wish to build and the values they seek to embody. This focus on workability (pragmatism) is prioritized over objective truth and language is approached as a social behavioral tool rather than being reflective of pathology or health.

A pragmatic approach to truth also allows for evolution, experimentation, and flexible implementation related to assessment and intervention. Rather than relying on ontological truth statements that may not be relevant to the individual’s goals, history, or current context, a focus on workability requires the clinician and client to examine the extent to which a client’s behavior in a particular moment supports or interferes with the pursuit of personally meaningful and socially significant aims. Case conceptualizing to identify a behavior’s functions (immediate consequences) through functional analysis, and to explore the workability of that behavior in the context of values and goals (longer-term consequences), is ongoing and constantly evolving.

### CBS and relational frame theory

CBS is grounded in functional contextualism as a philosophy of science, with Relational Frame Theory (RFT; 46) providing a behavioral account of human language and cognition inside this tradition. RFT is a theory of learning, language, and cognition in which human thinking is understood as a behavioral operant process (for a more detailed review, see 46). Actions (including verbal behavior in the form of thoughts) are conceptualized as behaviors that influence (i.e., operate on) an individual’s internal and external environments, thereby affecting the likelihood of future behaviors. Put simply, RFT explains how human beings learn to connect experiences, words, memories, emotions, and ideas together through language (i.e., relational frames).

As language develops in a particular area (e.g., thoughts about an experience) and patterns form (i.e., relational networks are established), these connections strongly influence how people experience themselves and the world. Importantly for moral injury, these relational networks can alter the psychological functions of experiences, such that events, thoughts, memories, or other stimuli can acquire painful moral functions without direct learning experiences. Furthermore, in the case of moral injury, once exposed to a morally injurious event, people’s previously innocuous experiences can come to elicit moral pain because language (thinking) can draw connections between disparate experiences.

As relational networks develop, an increasingly broad range of contexts may come to evoke moral pain, including interpersonal relationships and behaviors that were otherwise unrelated to the original morally injurious event. Given the generativity of thinking and the availability of potential triggers, targeting single symptoms for reduction or elimination may not be practical or efficacious, as this strategy is unlikely to stop moral pain from emerging again in different forms.

When internal experiences are conceptualized functionally rather than syndromally, thoughts and emotions are understood not as symptoms requiring correction, but as contextual events that influence the client’s ongoing patterns of responding. Accordingly, the goal of intervention in CBS is to alter the functional relations among behaviors and their contexts, thereby breaking the patterns that maintain suffering and creating opportunities for more flexible, values-consistent behavioral repertoires to emerge.

We will now revisit the case example from earlier, but instead of using a syndromal approach to case conceptualization, we will use CBS and RFT-informed principles to conceptualize the behaviors maintaining moral injury. When the veteran is reminded of the time he killed a child in war, he experiences intense shame, nausea, painful memories, and the thought, “I blame myself.” Over time, this thought becomes increasingly embedded in a broader relational network involving beliefs about being dangerous, unworthy, or incapable of being a good person. As the veteran’s life continues after deployment, this network of meaning extends into new contexts and relationships (47). When the veteran later becomes a parent, interactions with his children begin to evoke the same shame and self-condemning narratives associated with the wartime event. Seeing his children triggers thoughts such as “I blame myself,” “I am a monster” and “I will harm them,” even though the veteran is no longer in combat and poses no actual danger to them.

In response to these experiences, the veteran withdraws from his children to avoid shame, fear, and painful memories. This avoidance provides temporary relief from his emotional pain. Although avoidance reduces short-term distress, it simultaneously limits opportunities for closeness, connection, and reinforcement through interaction with his children. Over time, these patterns increasingly organize the veteran’s life around avoiding shame, rather than values-based connection with his children. The goal of intervention with this veteran would not be to remove morally painful experiences or decrease the intensity of moral pain, but to alter how these experiences function in relation to his behavior. As the veteran gains understanding of the functions of his behavior in response to moral pain (e.g., avoidance) and the associated consequences (e.g., reduced connection and engagement with his children), intervention can support more flexible responding in the presence of that pain. The veteran can build the ability to recognize and hold his pain while honoring the meaning behind it- that he wants to protect and support children- particularly his own. Rather than treating shame as a symptom to be reduced, it can be recognized as a signal of the veteran’s values and the opportunity to connect with, protect, and cherish his children. This functional understanding creates opportunities for values-consistent behavior, such as remaining engaged with his children even when shame, painful memories, and self-condemning thoughts are present.

## Acceptance and commitment therapy for moral injury

Having defined moral injury from a CBS perspective, we describe Acceptance and Commitment Therapy for Moral Injury (ACT-MI) as a contextual behavioral intervention and introduce its structure and testable hypotheses to evaluate its impacts in the sections that follow. We explore how moral injury is targeted in ACT-MI through processes grounded in RFT. Rather than deciding if past actions were morally justified or trying to remove, repair, or correct their painful consequences, in ACT-MI the focus is on helping individuals learn skills to relate to moral pain in ways that expand opportunities for meaningful living, connection, and flexibility. By building these skills, clients may pursue their values even in a world where their morals have been violated and may be violated again.

### The structure of ACT-MI

**Figure 1:**
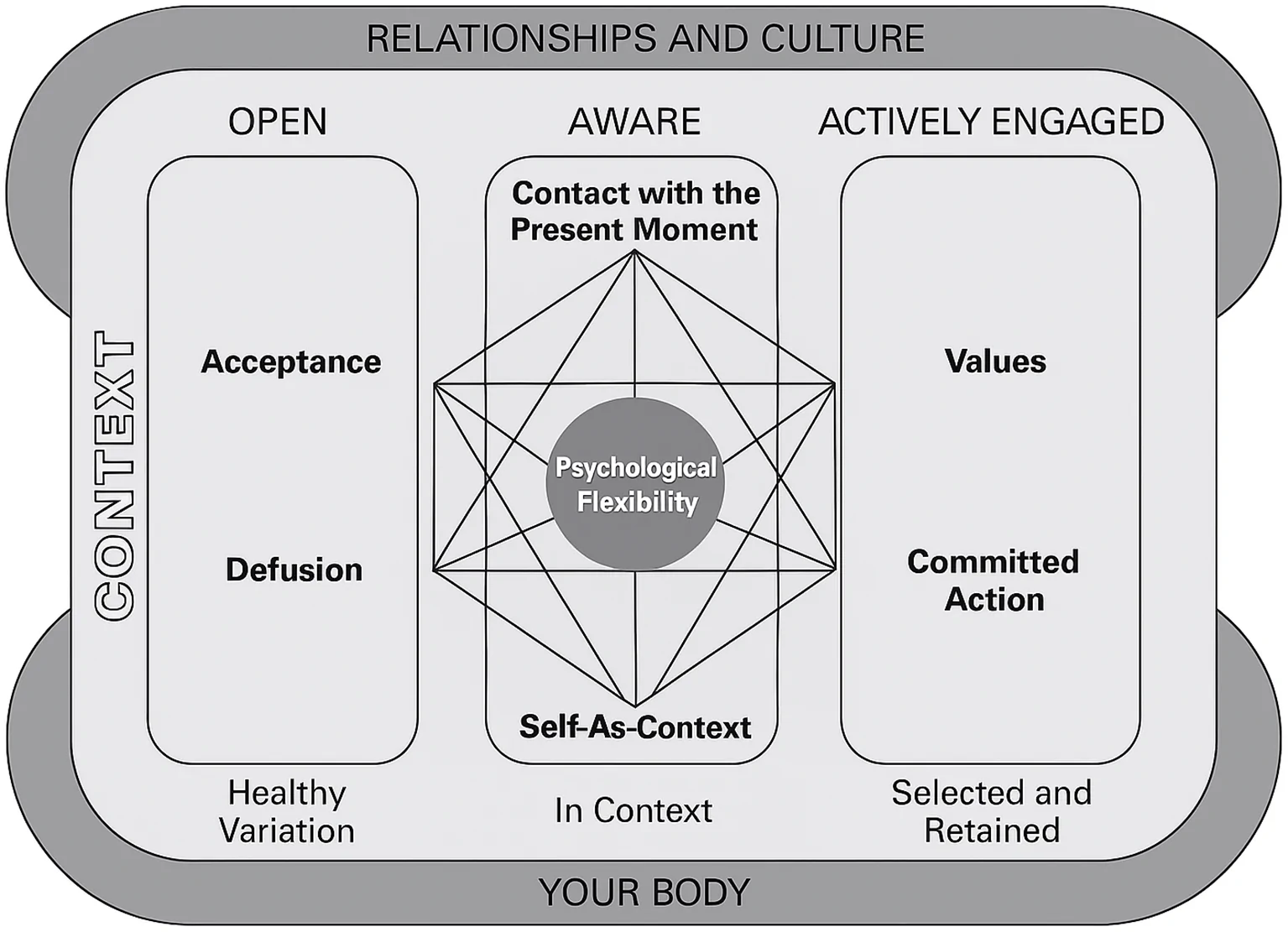
Hexaflex illustrating acceptance and commitment therapy pillars and processes. Adapted with permission from Roblyn Wasler.

ACT-MI is a specific application of Acceptance and Commitment Therapy (48) designed to support clients in learning skills to live their values even in the presence of moral pain. Like the general ACT model, ACT-MI is centered around six core processes that mutually support one another to facilitate the practice of psychological flexibility. These six processes fall into three pillars, each representing two similarly focused ACT processes (**Figure 1**). These pillars (with their respective processes) are Open (Acceptance and Defusion), Aware (Contact with the Present Moment and Self-as-Context), and Engaged (Values and Committed Action).

ACT-MI is organized as a 15-session hybrid group (12 sessions) and individual (3 case conceptualization sessions at sessions 1, 7, and 15) protocol that can be delivered in-person or virtually (see **Supplementary Image 1** for ACT-MI Clinician Outlines and **Supplementary Image 2** for ACT-MI Client Workbook). Individual ACT-MI sessions are 30 minutes and focus on disrupting the idiographic behavioral repertoires that maintain moral injury across clients, facilitating case conceptualization through formal functional analysis (chain analysis). Group ACT-MI sessions are 90 minutes and involve a skills review at the start of group (typically including an experiential exercise and a values review), followed by deepening the practice of a skill in the second half of group with an intensive experiential exercise and processing to facilitate generalization of the skill to be practiced in the client’s life.

Both individual and group ACT-MI sessions include experiential exercises that create sensory contexts for practicing relating to moral pain differently and connecting more fully with values. The first half of the treatment protocol (sessions 1-6) involves introducing ACT core processes to cultivate an observer’s perspective in the presence of moral pain, thereby increasing clients’ capacities to respond flexibly to these experiences and engage in values-consistent behavior. The second half of the protocol (sessions 7-15) builds on the skills introduced in the first half and helps clients practice engaging with ACT processes using novel content.

Borges and colleagues (2) conducted a randomized controlled pilot trial of 74 warzone-deployed veterans with the aims of determining 1. the acceptability of ACT-MI; 2. the feasibility of the research design for a future full-scale efficacy trial; and 3. selecting a psychosocial functioning measure for the future efficacy trial. Pilot trial findings revealed that ACT-MI was acceptable to clients, feasible to implement virtually, and may improve psychosocial functioning (2). A fully powered, large-scale randomized controlled efficacy trial is currently underway to rigorously evaluate the effects of ACT-MI on psychosocial functioning. We now describe how clients and therapists work collaboratively in ACT-MI to explore the functions of the behaviors that may maintain moral injury-related suffering. We then describe how the six core ACT processes are applied to build flexibility in responding to moral pain and support values-consistent behavior in both individual and group formats. Additional details are provided in the ACT-MI protocol (**Supplementary File 1**) and client workbook (**Supplementary File 2**).

## Case conceptualization and building motivation

### Individual level

ACT-MI begins with an individual case conceptualization session using chain analysis to explore the functions of behaviors maintaining moral injury (42; **Supplementary File 1** [p. 1-7] and **Supplementary File 2** [p. 6-7]). Chain analysis provides a method for organizing the streams of behavior relevant to moral injury, so that both client and therapist can recognize why the behavior persists and focus on disrupting the patterns maintaining it. Identifying the reinforcers that sustain suffering can foster openness to engaging with moral pain more flexibly, with the client learning that their behavior makes sense in context, and that behavior change may create opportunities to more meaningfully connect with values.

In ACT-MI session 1, clients explore the reasons they continue to engage in behaviors that maintain moral injury (short-term reinforcers), while considering the long-term costs of these patterns across different areas of their lives (e.g., relationships, self-care, spiritual practice, work). Often, moral injury maintaining behaviors facilitate avoidance of moral pain through negative reinforcement, temporarily reducing or removing an individual’s experience of moral pain (e.g., using alcohol to reduce guilt). Moral injury can also persist through positive reinforcement. In this case, behaviors in response to moral pain function to increase access to a desirable consequence not previously accessible; for instance, thinking about suicide in the presence of feeling overwhelmed by moral pain may facilitate a sense of control.

By using chain analysis to explore the short- and long-term consequences of behaviors potentially maintaining moral injury, clients begin to recognize how unworkable these patterns are in supporting the lives, relationships, and moral commitments they wish to cultivate. In sessions 7 (**Supplementary File 1**, p. 44-49, **Supplementary File 2**, p. 33-35) and 15 (**Supplementary File**, 1 p. 87-92, **Supplementary File 2**, p. 56-58), interventional strategies practiced in treatment are woven into the chain to help clients see how they might disrupt their patterns of avoidance and control maintaining moral injury. The client and therapist also practice these strategies in session while engaging the chain. Given the wide array of behaviors individuals engage internally (e.g., ruminating about their role in a moral violation) and externally (e.g., binge eating) to manage their moral pain, ongoing case conceptualizing through functional analysis is a crucial component of ACT-MI.

### Group level

Clients bring the avoidance strategies they identified in the first chain analysis session (ACT-MI Session 1) to the first group session (ACT-MI Session 2; **Supplementary File 1** p. 8-13, **Supplementary File 2** p. 8-16). The group then collectively generates a comprehensive list of all the strategies they have used to manage moral pain across their lives (e.g., substance use, isolation, eating behaviors, suicidal ideation, suicide attempts, therapy, medication, aggression). The facilitators guide the group to experientially connect with the strategies they have used to cope with pain, the years they have spent struggling with that pain, and the extent to which these strategies have “worked” to resolve or control it. This exercise serves to both destigmatize the process of moral injury for group members and motivates each group member to begin considering alternative, less costly ways of relating to their moral pain.

In the next session (ACT-MI Session 3; **Supplementary File 1**, p. 14-21, **Supplementary File 2**, p. 17-21), group members are guided to confront their struggles with moral pain through an experiential exercise called the “tug of war” (48). In this exercise, group members imagine an image that represents their moral pain (e.g., a monster, a radioactive rod, a heavy weight, a dark version of themselves). The image’s content is inconsequential as long as it conveys their moral pain. Group members imagine that a rope stretches between them and their moral pain across a deep pit. They are invited to imagine that they and their moral pain are positioned across each other on opposite sides of the pit, each holding one end of the rope and engaged in a desperate tug-of-war. As they pull, fighting to draw their moral pain into the pit, the group emotionally connects with the struggle (attempts to avoid painful internal experiences) and realizes that, despite years of collective effort, the moral pain has not disappeared. No matter how hard they fight, their moral pain remains. The attempts to eliminate their pain also comes at a high cost, moving them away from what they care most about (e.g., family, work, spirituality, health). If they do not decide to do so spontaneously, group members are presented with the option of “dropping the rope,” which means letting go of their struggle and accepting that their moral pain may continue. However, it also means their lives no longer need be consumed by the unwinnable tug-of-war. Instead, now with free hands, they can choose to pursue their sources of meaning and purpose even as their moral pain persists. The rope will continue to be available to pick up, but group members can learn to notice the urge to reengage in the struggle and choose to drop it again, making room for their moral pain to be present without organizing their behavior around efforts to control it. This exercise, and others with similar functions, create a context for choice—helping group members consider what might be possible if they continue to choose to drop the rope and approach moral pain.

By building on shared experiences of the unworkability of avoiding and attempting to control moral pain, and by introducing choice in how group members relate to moral pain, a social community begins to emerge, where group members find meaning in their interactions together. This community typically enhances the group’s willingness to practice alternatives to avoidance and control, including vulnerably sharing in session and expanding their connection with values outside of session. Indeed, the concept of values is introduced in early group sessions through a values card sort exercise, where group members practice choosing and connecting with different ways of living meaningfully. They generate examples of how to practice living those values throughout the week through small, deliberate behavioral actions.

Having examined the costs of avoidance and created a context for change, the facilitators next introduce the relationship between values and pain (ACT-MI Session 4; **Supplementary File 1**, p. 22-28, **Supplementary File 2**, p. 22-24). The purpose of this session is to continue supporting the willingness to “drop the rope” in the service of building additional, more workable strategies for relating to moral pain. To this end, members are invited to imaginally approach their image of moral pain from session 3 and sense what it is seeking – what it longs for. Group members begin to discover for themselves what their moral pain means. They learn that their pain is profoundly connected to their deepest moral values. Their pain signals what matters most to them; guilt may imply a value of integrity. Shame may imply a value of honor. Experiencing moral pain is a signal of their humanity.

The group explores the significance of this experiential connection between values and moral pain as “two sides of the same coin” (42), recognizing that what makes moral pain possible is also what gives life meaning: the capacity to care deeply about what matters. For instance, caring about people requires a willingness to feel pain along the way, especially when interpersonal interactions remind us of past moral violations. The ability to experience this poignant but painful relationship is supported by fellow group members who help one another to interact differently with moral pain. The reinforcing qualities of living values with a social community often motivate continued engagement in treatment despite the aversive nature of interacting with moral pain. In some cases, identifying the inseparable connection between pain and values begins to transform group members’ relationship with their pain from one of antagonism, to one they can appreciate, honor, and learn from.

## The open and aware processes

### Individual level open and aware processes

As noted, because moral injury is conceptualized to be maintained through efforts to avoid, control, and otherwise change moral pain, intervention strategies are needed to reduce reliance on these efforts and expand behavioral repertoires characterized by flexibility, choice, and values-consistent action. In ACT-MI, interrupting avoidance and control requires developing the capacity to observe and contact moral pain without immediately attempting to alter or escape it.

To help clients practice observing the rise and fall of moral pain, facilitators guide them to mindful awareness of their internal experience through moment-by-moment mindfulness interventions, as well as through formal group exercises during sessions and between-session practice exercises. ACT-MI facilitators invite clients to slow down and observe the experiences emerging in the moment (contact with the present moment), deliteralizing these experiences and noticing them for what they are whether in the form of thoughts, emotions, memories, physical sensations, or urges to act (defusion), and holding these experiences regardless of their content (acceptance). By staying grounded in the present, clients can better access their moral pain and what it signals about their values. Exercises focused on observing moral pain are always linked to the client’s values, reminding clients of the meaning behind their pain and empowering them to choose to relate to it differently so they can participate more fully in their life. Linking moral pain with values allows the client and clinician to also identify the most workable ways to connect with pain across the client’s environmental contexts (e.g., observing moral pain in session rather than practicing this skill in a potentially dangerous argument with their partner).

Allowing moral pain to rise and fall may be unfamiliar as most individuals have not been previously reinforced for intentionally connecting with their painful emotions, thoughts, and memories. Therefore, after introducing the observation of moral pain and building willingness to interact with it differently, one of the next ACT-MI targets is to strengthen the recognition of a context from which moral pain can be observed for what it is, engaging the critical ACT core process of self-as-context (the observer-you perspective). Creating recognition of such a context places clients in contact with themselves as a “place” where they can observe these experiences without automatically responding through avoidance or control strategies. Self-as-context processes cultivate this observer perspective, allowing clients to notice thoughts, emotions, memories, and urges without being fully defined or behaviorally constrained by them. Ultimately, building an observer’s repertoire means adopting a perspective that is more expansive than any collection of memories, thoughts, or emotions.

Self-as-context is particularly important when working with moral injury, as clients often become attached to their moral violations and see them as experiences that now define who they are and how they can behave in the future. In contrast, when practicing the observer-self perspective, clients directly experience a part of themselves that can “see” moral pain, and therefore, must be separate from the pain being observed. The moral violation is “part” of their identity, and they are the vessel for that experience. This widening of perspective also involves helping clients contact a sense of self as the point of view from which their experiences have been observed across time, even when that perspective has gone unnoticed.

To help clients practice the observer-self perspective, the therapist uses metaphors to increase experiential contact. For instance, the chessboard metaphor (ACT-MI Session 5; **Supplementary File 1**, p. 29-33; **Supplementary File 2**, p. 25-27) helps clients notice that in a way similar to how a chessboard holds its pieces but is not defined by them, they too hold their moral pain yet remain distinct from it. By inviting clients to “notice who is noticing” their moral pain, clients become more skilled at distinguishing self from experience.

From this point on in ACT-MI, each session focuses on experiential exercises in which some facet of moral pain is approached individually from an observer perspective (activating all open and aware processes). Different forms of content (e.g., emotions, thoughts, sensations, urges, memories) are engaged to build flexibility in holding moral pain and fluency in approaching it from multiple angles, thereby increasing flexibility across different elements of clients’ entangled networks (for an example see ACT-MI Session 6; **Supplementary File 1** p. 34-43). In doing so, clients decide how to live their values in response to what they directly experience, rather than relying on moral rules that are insensitive to context and may not bring them closer to what they care about.

The work of contacting moral experiences is deepened in the second half of ACT-MI, as clients are guided to connect with emotionally evocative memories. Clients practice identifying and intentionally experiencing a range of memories as part of a life timeline exercise, including painful memories associated with the morally injurious event and morally pleasant memories (ACT-MI Sessions 8-10; **Supplementary File 1**, p. 50-66, **Supplementary File 2**, p. 36-42). By practicing holding these observed memories, clients can acknowledge these past events as important points in their histories, while also recognizing that these experiences do not define them in the present moment. In the final group sessions of ACT-MI, clients are reminded that strengthening their capacity to observe internal experiences from a broader perspective can create greater space for values-consistent choice in the present moment (ACT-MI Sessions 11, 12; **Supplementary File 1**, p. 67-75, **Supplementary File 2**, p. 43-50).

### Group level open and aware processes

Though group members are asked to engage with their own pain during experiential exercises (e.g., practice observing the rise and fall of a painful moral emotion), they do so in the presence of other group members and facilitators. As a result, in ACT-MI group members witness each other’s interactions with their own and the community’s moral pain. Participating in ACT-MI invites a willingness to engage in experiential exercises together and to process these experiences within the therapy group’s social community. This practice of engaging with moral pain and then describing their experience of relating to it back to the group teaches group members that sharing pain can lead to increased connection and trust (rather than isolation and betrayal). Sharing in the context of group may generalize to veterans’ lives. That is, veterans learn that, in a group of trusted others, they are not rejected or betrayed for expressing their moral pain, which may encourage them to approach and share their pain in relationships outside of the group, with their families, friends, and others in appropriate social contexts.

While each experiential exercise includes processing among group members, some exercises involve group members directly interacting with each other’s pain. In ACT-MI Session 6 (**Supplementary File 1**, p. 34-43), each group member is invited to write down the moral violation they struggle with most on a piece of paper, then crumple it up, hold it in their hands, and observe the moral pain it evokes within them. They then describe the experience to the group, often aligning with the theme that their pain makes them more damaged or less human than everyone else in the group.

Next, group members are invited to exchange these “pain balls” with each other (without opening it or knowing what is written inside their partner’s paper) and observe their experience of holding another group member’s moral pain. Group members often describe holding another’s moral pain as a sacred experience and feel an urge to care for and protect it. Facilitators encourage the entire group to reflect on the discrepancy between their behavior in relating to their own pain through attempting to avoid, control, and get rid of it, and another’s moral pain through attempting to hold, honor, and protect it. Group members are invited to consider that their moral pain, including feelings of isolation and mistrust, represents a shared humanity present across the group.

Facilitators then invite the pairs to return their partner’s pain and to receive their own moral pain again, not to change it, but to intentionally choose to relate to it with the care and openness they showed their partner. This experiential group exercise creates a context for a social perspective on moral pain, broadening each group member’s perspective and allowing tangible practice in holding moral pain with openness. The social functions of moral emotions are activated in a live group context, and by holding their partner’s pain, participants learn that their partner can accept them regardless of what is written on the paper.

In addition to these planned experiential group exercises, in-the-moment functional analysis is used to identify and intervene on the potentially avoidant functions of behaviors as they emerge in the group. For instance, a group member might start telling a joke in response to a moment where a particularly evocative experience of moral pain is being discussed. In such cases, facilitators might invite the group member who made the joke to slow down and notice what they were experiencing immediately prior to telling the joke, and how their experience shifted after telling it. Facilitators then bring the group in and ask who else engages in these kinds of behaviors in response to pain. Avoidance as a strategy to cope with pain is identified, normalized, and recognized. The group is then invited to practice connecting with moral pain in the present moment rather than engaging in further avoidance.

## Engaged processes

### Individual level engaged processes

In contrast to the open and aware ACT processes focused on holding moral pain, engaged processes (values and committed action) are centered on helping clients explore and live their values. When people are entangled in verbal narratives about themselves and the world related to moral injury, they often become stuck in cycles that reinforce those narratives’ messages and limit their contact with the expansiveness of the world around them. These narratives are often reinforcing in the short term, in that relying on their predictions facilitates the avoidance and control of the moral pain that may arise in the context of living freely. Such stories can shape future behavior and limit people’s behavioral repertoires. For instance, imagine a person’s willingness to introduce themselves to someone new while a narrative about being a monster who hurts others runs through their mind. The narrative becomes part of the current context they consider when choosing future behavior. In ACT-MI clients learn that rather than allowing this narrative to dictate their behavior, they can make room for its presence while choosing an action guided by what matters to them.

This broader perspective can open possibilities for valued action that extend beyond the futures prescribed by clients’ stories. Communicating this sense of possibility, Kelly Wilson, one of the original developers of ACT once shared in a training, “the goals I hope for clients are the ones they can’t even think of yet” (49). Clients do not necessarily enter treatment knowing what their values are or what is meaningful to them. Indeed, for an individual who has lived a restricted, narrow life governed by avoiding and controlling pain, the idea of connecting to expansive possibility can feel an extremely overwhelming prospect. Rather, the opportunity to freely choose behavior that may not have been considered possible previously is at the core of working with values in ACT—choosing based on yearning and allowing that yearning to guide meaningful action.

Clients practice small, meaningful moves (committed action) that, on the surface, may not appear impactful, but over time and across contexts, create a foundation for significant changes in their lives. A metaphor often shared in session is that small, seemingly inconsequential, values-based actions add richness and flavor to life, in the same way that soup stock requires many basic ingredients and time to develop depth – even the most basic ingredients are consequential (49). The more exploration, persistence, and care taken, the more possibilities are available.

Across ACT-MI sessions, clients repeatedly engage with their values to expand possibilities for meaningful living. This sense of expansiveness can be difficult to evoke when values work feels routine or obligatory (e.g., being assigned a set of committed actions every week). When values are treated as destinations or goals rather than ongoing directions or ways of being, committed action can become rigid and rule-governed, narrowing rather than expanding behavioral repertoires (e.g., “I just have to get a job and then I can have a meaningful life”). Emphasizing values as ongoing qualities of action instead keeps attention focused on how clients choose to live in the present, including when desired outcomes cannot be controlled. The choice to connect with values as directions rather than goals is particularly important in ACT-MI, where rigid pursuit of prescribed outcomes may itself function as a way to avoid or control moral pain (e.g., living by rules because the outcome is known).

The distinction between values and rules is particularly relevant when considering repair, a component of many moral injury treatments (32–35). However, although it may often be congruent with a clients’ values, repair in and of itself is not a necessary element of engaging values within ACT-MI. Attempts to repair a moral violation (e.g., donating money to a group the client hurt) may overtly appear healthy for the client, but may at times function as another means of avoiding and controlling moral pain, such as when repair involves rule governed behavior (e.g., “If I volunteer to help the group I hurt, then I will be forgiven”). If the client focuses on attaining a specific outcome through repair (e.g., feeling better), such behavior may not actually allow connection with meaning in the present moment and may facilitate controlling moral pain and potentially maintain moral injury. If efforts to repair are guided by a client’s values (e.g., contributing) and freely chosen (e.g., connecting with the present moment with an expansive quality and not engaged to feel less pain or a more desirable experience), then repair-related behavior can conceptualized as living values.

To that end, chain analysis may be useful in determining the functions of repair-related behavior. An additional note concerns the language of repair. If repair is defined based on its definition as “fixing something that is broken,” then clinicians may need to be cautious in using this or similar terms lest they accidentally imply that the client (or their internal experience) is a problematic symptom to be eliminated and that corrective behavior (repair) will somehow change that internal experience. We believe this can represent another example of attempting to change experience rather than living a meaningful life informed by moral pain. If the client is in a scenario where repair is mandated (e.g., community service related to their moral violation), ACT-MI clinicians work with the client to identify and connect with their values even in the context of such external social prescriptions.

One way to engage values from a new perspective is to explore contexts that activate values through pleasant moral emotions (e.g., caring, gratitude, awe, elevation) rather than only identifying values through pain. An experiential exercise called the “sweet spot” can help to elicit this expansivity (50). In the “sweet spot” exercise, clients are asked to recall a sweet, cherished memory. Clients are guided to connect with the emotions, thoughts, sensations, urges, and other experiences that arise in the memory’s presence. After connecting with what the memory evokes, clients are asked to consider what it also signals matters to them, (i.e., what makes this memory sweet? Why is it meaningful)?. This practice allows clients to consider how they can continue living the essence of this cherished memory in the present moment and going forward. In other words, “what lingers after the fireworks have worn off” (51, 52). Clients process the internal experiences that engaging in the sweet spot exercise has activated for them, as well as the potential values it points to. Clients then explore ways they might live that value in the present moment, and the small actions they can take moving forward that represent the essence of why this memory matters to them.

## Group level engaged processes

In the authors’ collective experience, most people seek treatment for moral injury because they care about social values and have significant difficulty engaging meaningfully in interpersonal relationships. One of the most powerful elements of ACT-MI is the opportunity to build meaningful social relationships among group members and to use this momentum to deepen and expand relationships in clients’ personal lives. For instance, thoughts associated with shame are experienced about the self in relation to others (e.g., “I am bad and will contaminate whomever I’m around”), so those who struggle with these experiences tend to disconnect or isolate in their relationships. Those who have experienced moral violations in the form of betrayals often struggle with other-directed emotions like anger and contempt and may become attached to narratives that support these experiences (e.g., stewing in anger and mistrust as a way to protect themselves from additional betrayals). This strategy can lead to hostility and aggression in relationships, particularly when elements of the moral violation are activated (e.g., feeling unseen or deceived). In either case, finding connection and meaning in the group itself often has a repertoire-expanding quality for group members, helping them more flexibly relate to others in their lives.

The sweet spot exercise (ACT-MI session 10; **Supplementary File 1**, p. 62-66) is also used to create a context for group-level connection as a social community. As part of the exercise, group members are asked to share their sweet, cherished memory with the group and share what was evoked. This sharing typically elicits variability in moral emotions and experiences, both pleasant (e.g., appreciation for deceased loved ones) and painful (e.g., mourning their passing).

The goal of these group-level processes isn’t to change the group’s mood or to use pleasant moral emotions to overshadow moral pain, but to connect with the expansive nature of values and the variability of emotions they are capable of experiencing. Exploring what might be possible facilitates the group’s connection with freedom and choice together. As another example of how values are engaged at the communal level, in the final group (ACT-MI Session 14; **Supplementary File 1**, p. 82-86, **Supplementary File 2**, p. 54-55) members are asked to reflect on a moment from the group that was meaningful to them having similar functions as the sweet spot exercise but often explicitly related to the impacts of group members’ behaviors on each other.

### How engaging in ACT-MI facilitated living values in the presence of moral pain

Now that ACT-MI has been described, we will identify how ACT-MI was engaged with the case of the Veteran whose moral injury development we have presented from both syndromal and CBS perspectives. The veteran participated in ACT-MI and learned to observe his moral pain and the memory of the moral violation as experiences separate from himself and his identity. To do so, he needed to expand his repertoire of perspective taking skills, identifying his behavior of killing a child in the war as an experience in the past separate from his present moment experience. He learned to use hierarchical framing (53) to see his past, the behavior of killing in war, as “a part of” his experience but not all of his identity. He learned this through experiencing that because he can *see* (observer-self perspective) his pain, he can’t *be* his pain (he is more than his pain because he can observe it). Nausea and “I blame myself” became cues to “hold [his] shame” and his children became cues for connection rather than avoidance. The veteran learned to respond to his values regardless of his experiences, holding killing a child as an act in context.

We worked together to build a behavioral repertoire increasing flexibility in interacting with thoughts like “I am a monster, so I must avoid my children” using self-as-context and contact with the present moment. To build a “healthy selfing” repertoire (53), where the veteran’s experience of himself was bigger than any one event, we practiced noticing the variability of the veteran’s experiences over time. To more fully disrupt the stimulus control of “I am a monster, so I must avoid my children” over the veteran’s behavior, we built on the perspective that the veteran is the place where his experiences happen, deepening his experiential contact through the chessboard metaphor. He is the one who holds “I am a monster…,” his physiological experience of shame, his memories, and his current experiences. We expanded on this perspective through questions like, “*who has that shame … where does the thought “I am a monster” exist?”* Through relational framing, the veteran learned that, because he holds shame, he must be bigger than it. From this context, he could experience shame in the presence of his children and “hold it” while still choosing to interact with his children. His behaviors in response to moral pain shifted from avoidance to approach and connection.

### Assessing change in treatment, hypotheses, and future directions

While functional analysis is a key method for case conceptualizing and measuring progress across ACT-MI treatment, additional methods of assessing change are important in evaluating the efficacy of the intervention and generalizability. Psychosocial functioning and psychological flexibility processes are critical to measuring ACT-MI outcomes and processes, respectively. To better understand the impacts of ACT-MI on psychosocial functioning, our team is currently conducting a national randomized controlled efficacy trial pre-registered in clinicaltrials.gov (Identifier: NCT0771052). This study will tell us whether ACT-MI improves psychosocial functioning above and beyond Present Centered Therapy for Moral Injury (PCT-MI) and will provide insight into potential mechanisms of treatment. We will use symptom measures of moral injury (e.g., the MIOS) in the trial to understand how our sample of veterans compares with others in different treatment studies. Related to functioning, we hypothesize that participants in ACT-MI will report significantly greater improvements in psychosocial functioning at post-treatment relative to pre-treatment on the Outcome Questionnaire-45 (OQ-45; 54). We will explore whether changes in psychological flexibility and inflexibility scores on the Multidimensional Psychological Flexibility Inventory (MPFI; 55) mediate pretreatment to posttreatment changes in OQ-45 functioning scores. If psychological flexibility is found to be a mechanism of change impacting functioning in ACT-MI, learning to live values while holding moral pain may be identified as a key element of moral injury treatment. In exploring the relationship between values and pain, we hypothesize that engagement in values (MPFI) will predict better psychosocial functioning (OQ-45) even in the presence of moral pain (MIOS).

In addition to assessing psychological flexibility and functioning using self-report measures, assessment practices in CBS research warrant consideration. Self-report measures have significant limitations; they rely on client understanding of items, accurate identification of symptoms (including the challenge of locating moral emotions), and they often do not reflect state-based experiences. Additionally, typical statistical methodologies for evaluating psychosocial functioning and psychological flexibility using these measures involve comparing pre- and post-treatment changes.

Analyzing group-level changes through averages rather than individual changes over time may not accurately characterize behavioral change for each individual (56). Novel assessment methods are needed to track changes in individual level psychological flexibility in relation to moral pain including ecological momentary assessment (EMA) and behavioral coding of group and individual psychotherapy sessions, assessing indicators of open, aware, and engaged ACT-MI processes. EMA could facilitate better understanding of individual change trajectories and paired with statistical methods like Group Iterative Multiple Model Estimation (GIMME; 57, 58), allow for sophisticated individual- and group-level analysis without assuming the same trajectories across clients. Behavioral coding in individual and group sessions could be used to assess markers of treatment progress including variability and frequency of emotional expressivity, variability in communication related to sense of self (not just me = moral violation), and connection with other group members.

Additionally, evaluating treatment moderators and differences in treatment structure (e.g., hybrid vs. individual vs. group) would help us better understand ACT-MI’s impacts. For instance, we would hypothesize that social connection within treatment cohorts will moderate the relationship between moral pain and psychosocial functioning such that greater connection will be associated with improved psychosocial functioning.

Future research is needed to evaluate the impacts of ACT-MI on psychosocial functioning and to understand treatment mechanisms, and potential moderators. Additionally, research is needed to better understand the most efficient and effective methods of assessing flexibility in responding to moral pain and engagement with values. Future research is also needed to evaluate which moral injury interventions work best for which people.

### Summary

Just because syndromal conceptualizations of psychological suffering are the status quo does not mean that these models should be the default for working with issues of morality in treatment. In this paper, we present the potential problems inherent in conceptualizing issues of morality syndromally and offer an idiographic approach to assessment and intervention, rooted in CBS and RFT, as a robust alternative to a syndromal approach. We describe ACT-MI as a clinical application of CBS, that may allow providers and clients, as equals, to connect with the power of moral pain rather than treating it as a symptom to be extricated, reduced, or repaired. ACT-MI was rigorously developed in the context of a randomized controlled pilot trial (2) and is currently being evaluated in a large-scale efficacy trial of warzone-deployed veterans where psychosocial functioning is the primary treatment target.

While our research is veteran focused, given the flexible and idiographic nature of the intervention, the treatment content (the focus on relating to moral pain while living values) and structure of ACT-MI (hybrid group and individual intervention with virtual treatment) are likely applicable to a broad array of clients struggling with moral injury from different sources (e.g., healthcare workers, teachers, law enforcement personnel, refugees, social activists, those who have experienced significant racism, sexism, homophobia or other forms of prejudice). Following the efficacy trial, we will have more information about how this approach impacts veterans’ lives and if it makes sense to invest in widely disseminating this framework and continuing to evaluate its effectiveness for different types of clients beyond veterans moving forward. Formally evaluating an intervention grounded in a testable theory of moral injury development will facilitate scientific progress, hopefully enabling efficient and effective intervention.

## Data Availability

The original contributions presented in the study are included in the article/**Supplementary Material**. Further inquiries can be directed to the corresponding author.
