# Supplementary material for: Acceptance and commitment therapy for moral injury: conceptualizing and intervening on moral injury through the lens of contextual behavioral science
Source: Front Psychiatry. 2026 Sep 9;17:1923699. doi: 10.3389/fpsyt.2026.1923699 (PMC13597758; doi:10.3389/fpsyt.2026.1923699)
Supplement: Supplementary file 1 [file DataSheet1.pdf]

Acceptance and Commitment Therapy for Moral Injury (ACT-MI)

Client Workbook

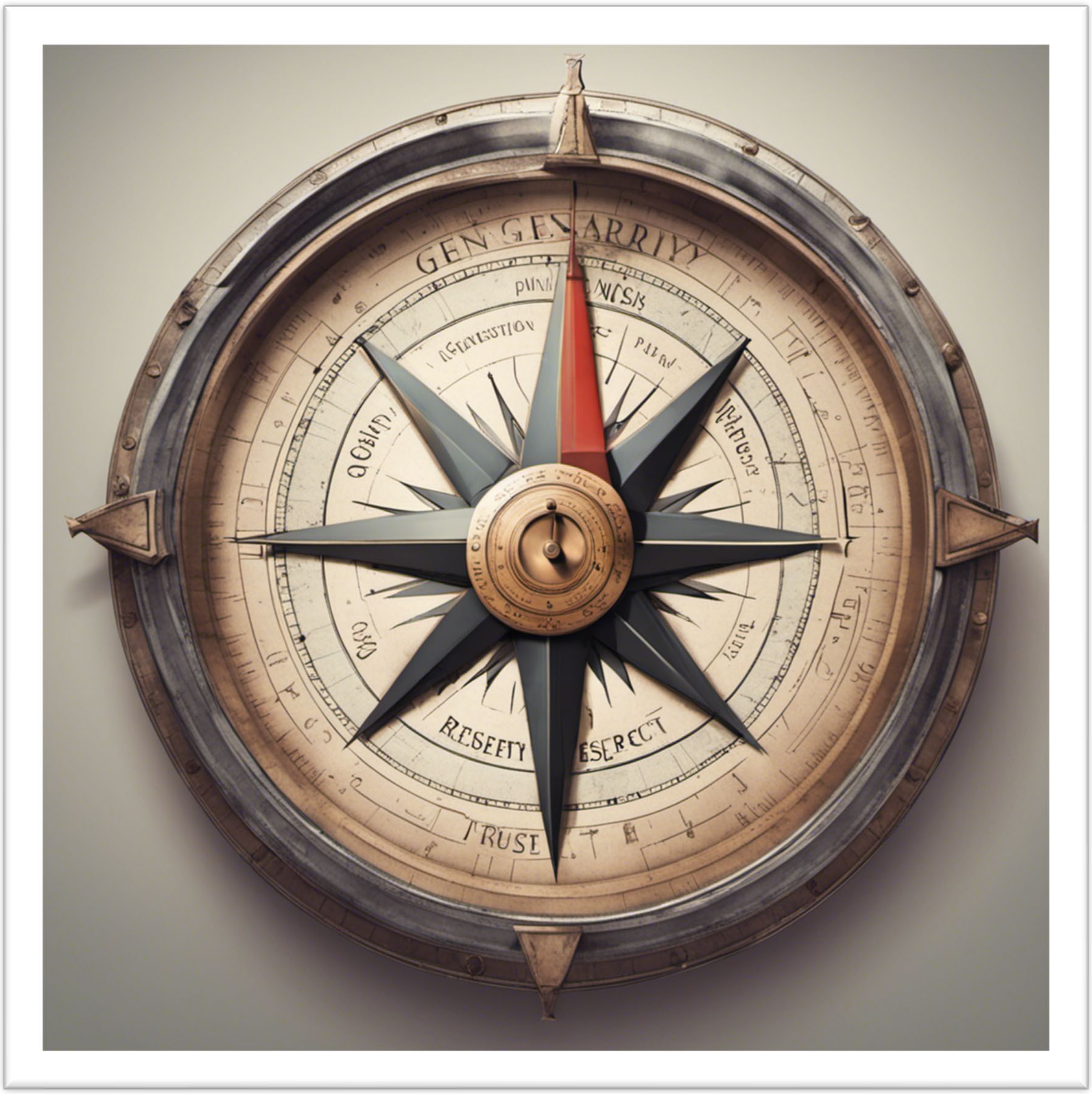

Borges*, L. M., Farnsworth*, J. K., Barnes, S. M., Drescher, K. D., & Walser, R. D. (2026). Acceptance and Commitment Therapy for Moral Injury (ACT-MI) Client Workbook.
*Both authors contributed equally to this workbook

CONTACT INFORMATION

**Facilitator Name(s):**	**Phone Number: (extension)**
______________________	______________________
______________________	______________________

Veterans Crisis Line: Dial 998 (press 1 for Veterans)

In case of a mental or medical emergency call 911 or go the nearest Emergency Department

ACT for Moral Injury Group Member Expectations

Expectations for all ACT-MI Groups

- Show respect to other group members and to group facilitators by arriving on time for session, having completed practice exercises and being ready to share comments and questions related to the general focus of the group.
- Do not come to session under the influence of substances or having recently used substances.
- Do not record sessions.
- Do not eat or use tobacco during session.
- As mental health providers, ACT-MI facilitators are required by law to help prevent imminent harm. In some very rare cases, the facilitators may need to break confidentiality in order to protect the safety of a group member or others. Facilitators will discuss this step with a group member before taking any such action.
- Group members can reach out to the ACT-MI facilitators during normal work hours (see workbook for contact info).
- For crisis situations outside of normal work hours, take any of the following steps:
    - Call the Veteran's Crisis Line (Dial 988, then press 1)
    - Call 911
    - Go to your nearest emergency department

Additional Expectations for Virtual ACT-MI Groups

- To preserve confidentiality, group members should join from a private room where they are alone.
- Do not drive or be riding in a car during group. You may call in from a parked vehicle if you are alone.
- Be fully dressed and in an upright position.
- Have devices plugged in or fully charged prior to the group.
- Place your device in a position where the camera can see your full face.
- Try to minimize distractions in your surroundings, such as unnecessary noise or movement.
- Cell phones should be in airplane mode or turned off if not being used to join session.
- Do not text, use email, use the internet, or engage in any other activities during session.

NOTES

NOTES

Starting to See Connections

People naturally avoid painful experiences and seek out experiences that provide pleasure or relief from discomfort. Although this can be helpful in the short-term, sometimes our tendency to avoid pain and seek pleasure can create suffering in the long-term, leading to patterns of behavior that prevent us from living meaningful lives. These behaviors may take the form of social isolation, substance use, considering suicide, aggression, and other costly or harmful behaviors. We may want to change these patterns of behavior but not know how. We may not feel that there are other options, since we've used these behaviors for such a long period of time.

Chain analysis is one way to understand the behaviors we engage in that cause moral injury to persist. Doing a chain analysis can help us understand why we behave the way we do and can also help us explore new possibilities for breaking the chain, influencing how we might behave in the future.

1) Start with picking a behavior that causes suffering in your life and you want to understand better (we call this the "target behavior"). It might even be a behavior you have thought about stopping in the past. Common examples of target behaviors in moral injury are isolation, alcohol or substance use, thoughts about suicide, aggression, or being rigid or inflexible.

2) Next, ask yourself, "What triggered the target behavior this time?" You probably don't engage in this behavior every second of every day. That means something happened to trigger it. Do you remember what specifically was going on that prompted you to do the target behavior this time?

3) Explore what happened between the triggering event and the target behavior. This should help tell the story of what happened between the triggering event and the target behavior, including the thoughts, emotions, sensations, and urges you remember, your behaviors—how you acted before the target behavior, and things that were happening in the situation that influenced you. Each experience becomes a link in the chain that connects the triggering event to the target behavior.

4) Identify what your emotions, thoughts, sensations and urges were right before the target behavior. See if you can rate the intensity of these experiences.

5) Describe how your emotions, thoughts, sensations, and urges changed . Was there a slight or big change in your own experience **during and right after** the target behavior? Did the behavior change how other people acted in the moment?

6) How does this behavior impact you in the long-term? Did it bring you closer to or farther away from the things that matter to you? For example, did it help you live with more meaning and purpose, or did it move you away from the things you care about?

Eventually, as we learn about the behaviors causing us suffering, what prompts them, and why we keep engaging in them over time, we will learn to use the skills and concepts of Acceptance and Commitment Therapy to break these patterns, create more choice in the way we live, and use that choice to live our values in a deeper and more purposeful way.

Session 1 Practice Exercise

We invite you to practice doing chain analyses on your own by following the instructions on the blank Chain Analysis Worksheet included in this section of the workbook. You can pick any behavior you want to better understand and do a chain analysis on it. Here are some tips:

- Pick a specific and recent action of your own as the target behavior. For example, choose “Turned down invitation to family gathering and played video games in the basement” instead of “isolating” or “Yelled at other driver” instead of “road rage.” Do not choose someone else’s actions as the target behavior.
- Choose a triggering event that is within hours or minutes of when the target behavior happened, instead of being in the more distant past (not days or years).
- If it’s hard to identify what triggered the chain of events leading to the target behavior, you can try working backward, identifying what you thought, felt, saw, experienced right before the target behavior and before that and so on until you reach the triggering event.
- If you have trouble identifying links in the chain, remember the triggering event and ask yourself “What happened next?” See if you can play back the situation in slow motion and catch additional parts of the event or your experience you may have not noticed before.
- It’s OK if you get stuck or confused. This is a different way of practicing looking at your behavior. You might want to consider reaching out your ACT-MI facilitators so they can help guide you.

Breaking Patterns of Moral Injury Worksheet

Step A: Write down in **Circle 1** something you did in response to feeling shame, guilt, anger, disgust, or contempt that would be helpful to understand.
Step B: What triggered the target behavior **this time**? Write this in the circle labeled **2: What triggered the target behavior this time?**
Step C: How did you get from the **trigger in Circle 2** to the **target behavior in Circle 1**? Fill circles **3-9** with the thoughts, emotions, sensations, urges, behaviors, or actions that happened in between the trigger **(Circle 2)** and the behavior you're trying to understand better **(Circle 1)**.
Step D: What happened to your *internal experiences* immediately after the **target behavior**? Write this in **Box 10**. (Rate the intensity from 0-100)
What happened to your *external experiences* immediately after the **target behavior**? Write this in **Box 11**. (Rate the intensity from 0-100)
Step E: What are the long-term impacts of the **target behavior**? Write these in **Box 12.**

2: What **triggered** the target behavior *this time*?

3.

What happened next?

4.

5.

6.

7.

8.

9. (Just before the behavior)

1: Target Behavior

10: What happened to **my** thoughts, emotions, sensations, urges, and memories right after? (*internal experiences)*

11: How did **others** respond, or my surroundings change, right after? *(external experiences)*

12: What are the **long-term impacts** of the target behavior in Circle 1? (e.g., Relationships, spirituality, Impacts on mental and physical health)

SESSION 2: INTRODUCTION TO ACT FOR MORAL INJURY

What is ACT-MI?

Acceptance and Commitment Therapy for Moral Injury ('ACT-MI' for short) is a treatment designed to help individuals pursue what matters to them even in the presence of painful moral emotions and thoughts. ACT-MI is based on ACT, an evidence-based psychotherapy with over 1000 randomized controlled trials supporting its benefit for different conditions.

Understanding Moral Injury

In defining moral injury, we would like to introduce the experiences that can lead to the development of moral injury and the behaviors that cause it to persist.

A *Morally Injurious Event* is one of more events that represent a departure from your personal values in a deep and profound way. Service members can come contact with morally injurious events in different ways. These include:

- Directly causing the event yourself
- Failing to prevent or stop the event from happening to someone else
- Witnessing the event happen to someone else
- Learning about the event after it was over
- Having the event directly happen to you

Moral Pain is <u>the natural and expected pain</u> we feel in response to a morally injurious event. This pain can take different forms, including emotions (e.g., guilt, shame, anger, disgust), thoughts (e.g., blaming yourself or others for the morally injurious event), physical sensations (e.g., turning stomach) and urges to act (e.g., urge to isolate or attack). Moral pain can occur during or after the event has ended.

Moral Injury is <u>the additional suffering</u> we experience when our own efforts to avoid, control, or escape moral pain limit our ability to meaningfully live our values. For example, socially isolating may help us avoid the feeling of shame in the short term, but eventually lead to weakened relationships.

The focus of ACT-MI is on **living your values even in the presence of moral pain.** This includes…

1) Learning to notice and carry the moral pain with you in the here and now for the sake of living your values.
2) Connecting with a you that is bigger than any event, including those of the past and future… a you that is bigger than any emotion or thought… a you that observes experiences instead of being defined or controlled by them.
3) Identifying and acting on what matters most to you, your core values, including any that you may have departed from during or after the morally injurious event and those values that may connect you to others around you.

This process of moving forward from moral injury is also depicted in the figure on the next page.

1. A morally injurious event (MIE) causes moral pain

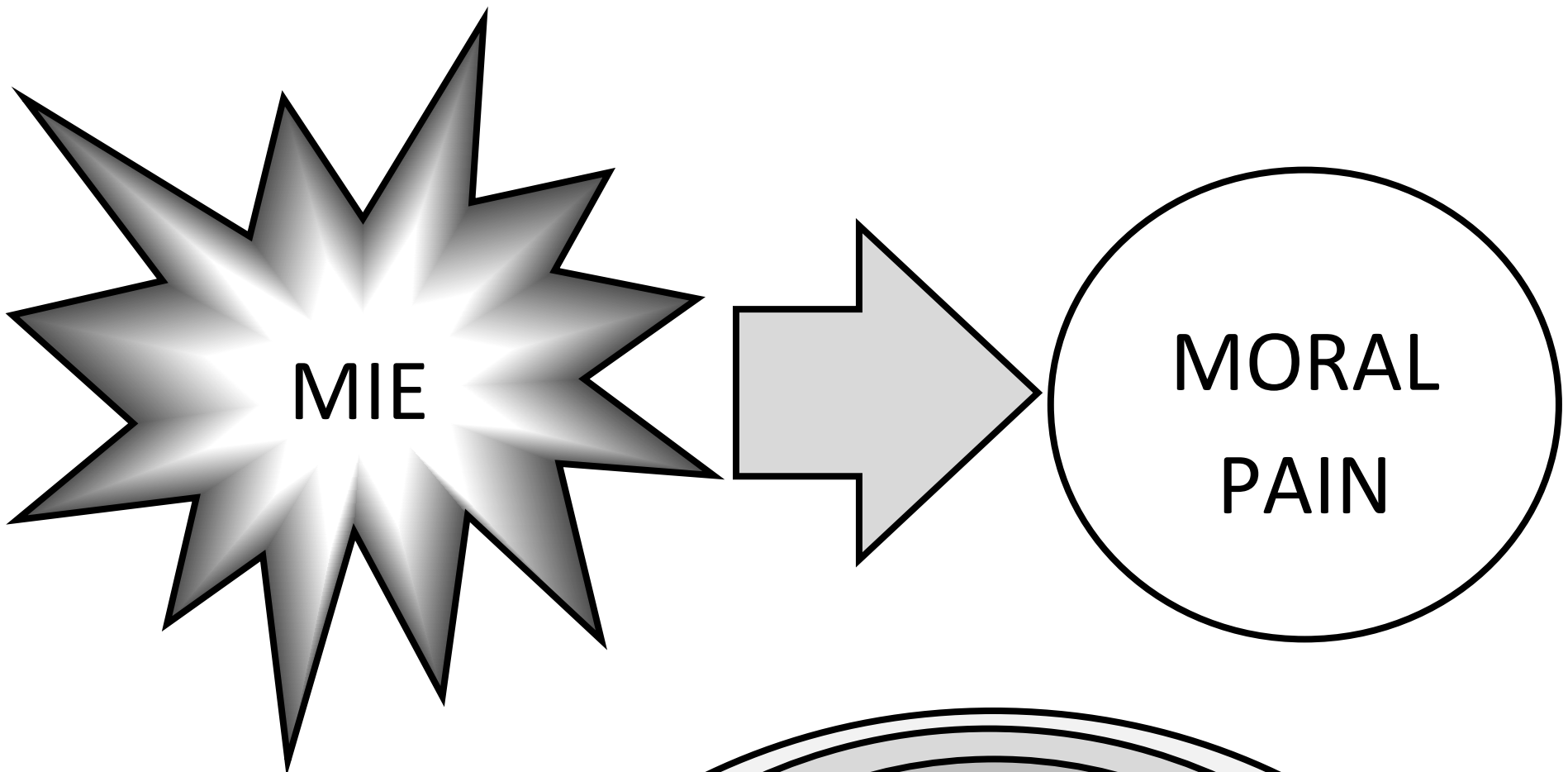

2. Moral injury creates new suffering as we try to escape or control moral pain

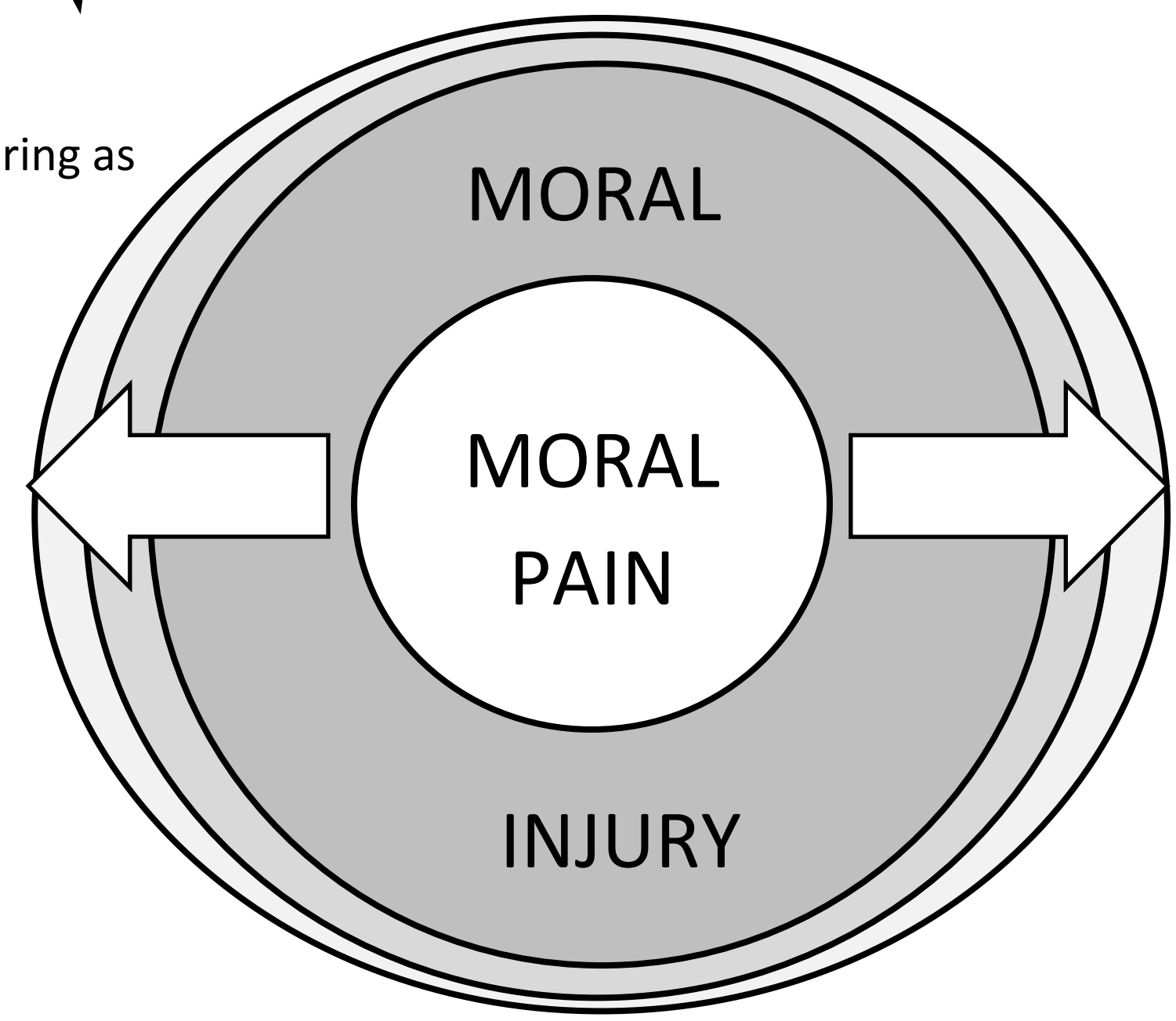

3. Living our values stops the morally injuring behaviors and reconnects us to meaning and purpose.

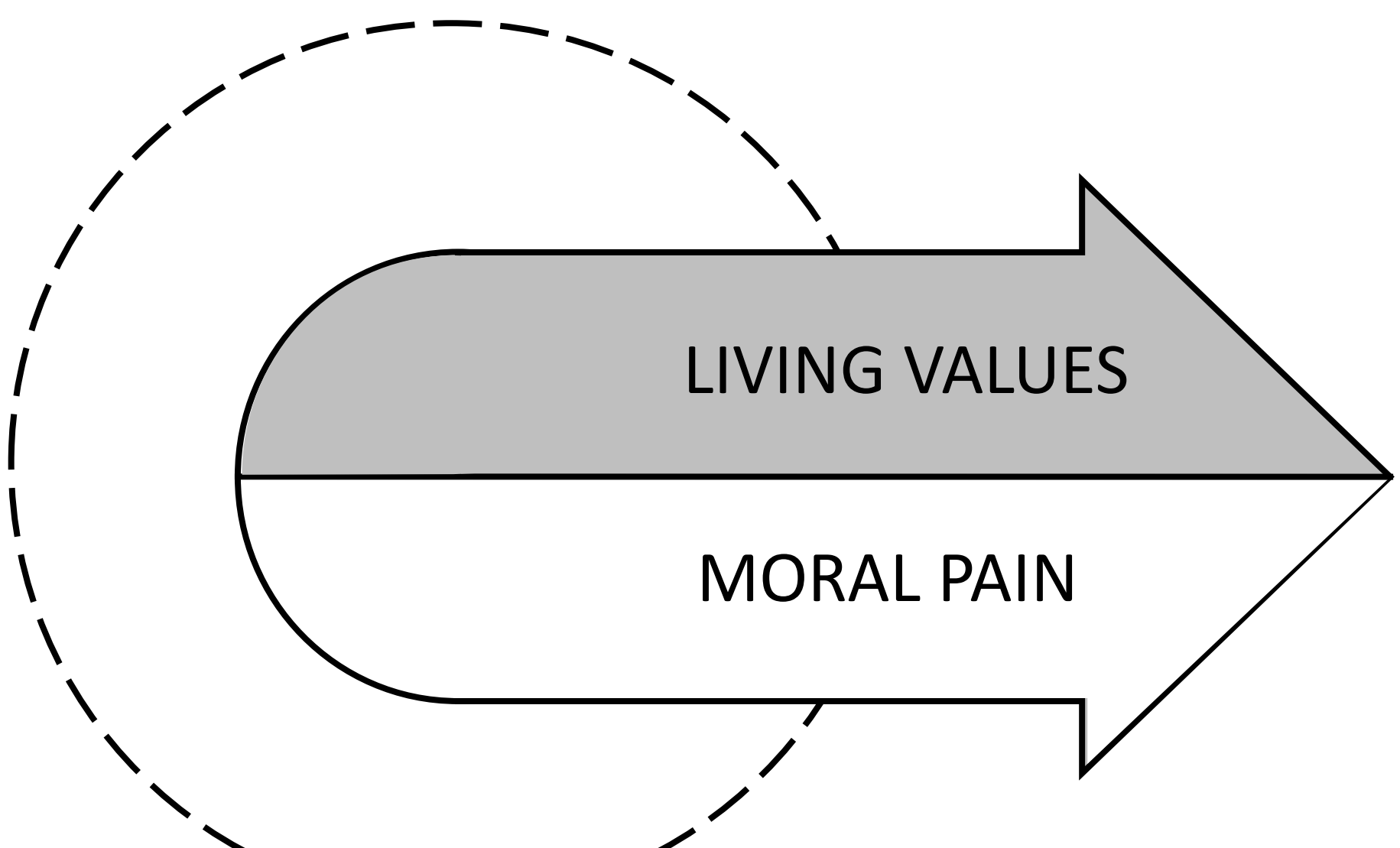

Identifying Morally Injuring Behaviors

Separating our moral pain from our moral injury is an important step in learning how to live our values more fully after a morally injurious event. We can do this using the diagram below.

First, take a moment and reflect on the new thoughts, emotions, sensations or urges that appeared after the morally injurious event(s) happened. In the inner circle below, list words or phrases that capture this moral pain (guilt, shame, anger, I don't deserve to…, etc.)

Next, in the outer circle, list the strategies you have tried to avoid, control, or solve your moral pain (alcohol, isolating, self-punishment, suicidal thoughts, medication, even therapy). What do you notice? Have the strategies worked in the long run? What have the strategies cost you and others?

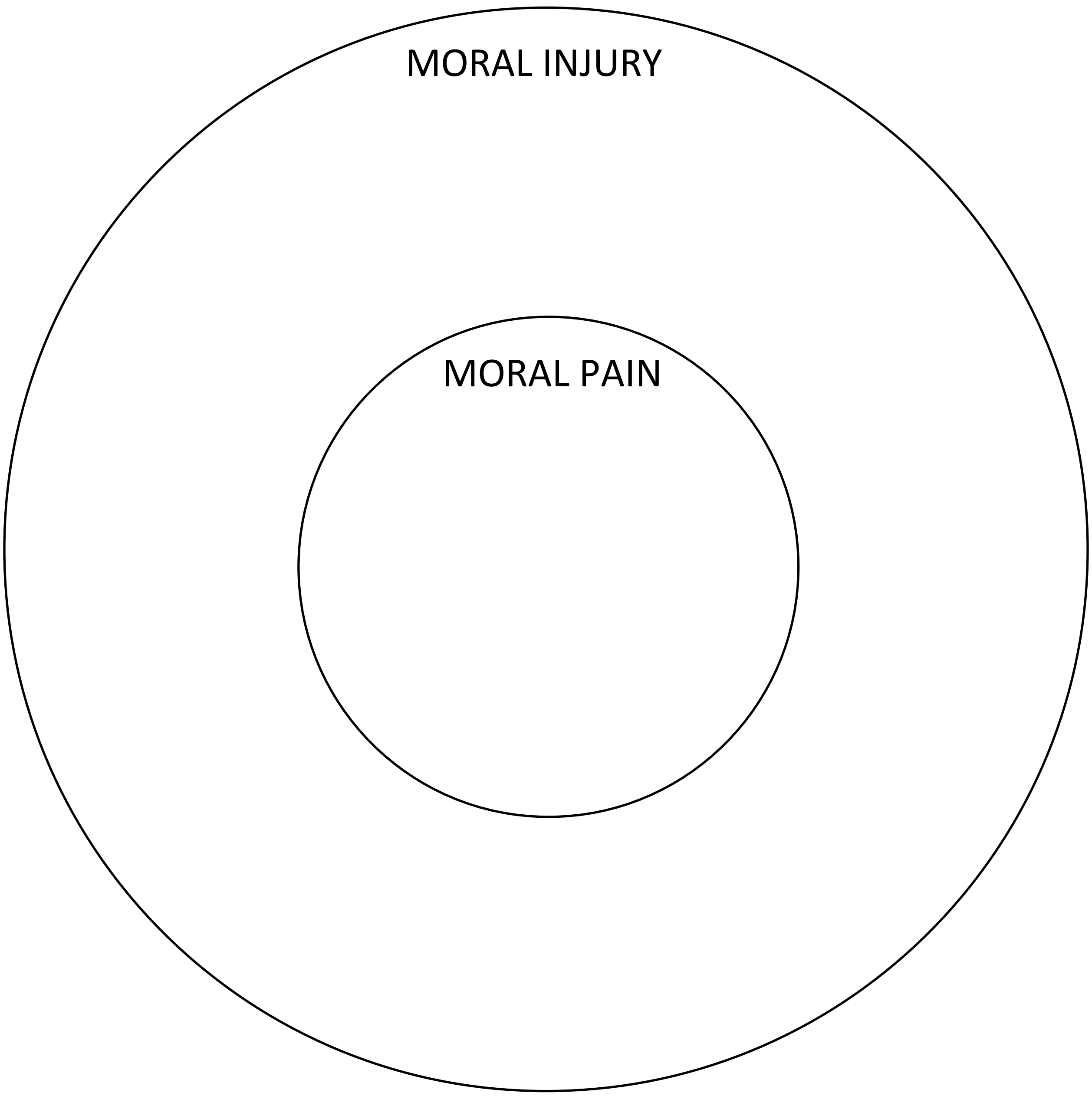

The Core Skills of ACT

ACT-MI is an application of a more general therapeutic approach called Acceptance and Commitment Therapy or ACT for short (said as the word "ACT"). ACT teaches us skills for learning how to work with painful experiences like moral injury in ways that allow us to move forward with our lives and what we care about. Your understanding of each core skill will grow as you attend sessions, engage in exercises, and practice these skills on your own between sessions.

The first skill we have begun practicing is Being Present. This skill involves allowing our attention to rest completely and entirely on the current moment as it unfolds around us. We can be present with our internal experiences (thoughts, emotions, physical sensations, urges to act) or our external experiences (sights, sounds, other people). When we are present, we notice our urges to act before they are turned into actual behaviors. Because of this, being present allows us to act with choice, rather than reacting out of mindless habit or instinct. In this way, being present is the first step in learning to living our values more fully.

The table below lists other ACT-MI skills that we will be focusing on together:

ACT-MI SKILL	DESCRIPTION
Being Present	Slowing down and noticing what is happening in the present moment. Staying grounded in the here and now and all that the present contains.
Accepting	Opening up to and allowing emotions, thoughts, or other internal experiences to be in this moment, just as they are. This does not mean we enjoy the experiences or condone the events they are connected to. Rather, we acknowledge and stop fighting against the reality of the events and the experiences themselves.
Seeing Thoughts as Thoughts	Disentangling ourselves from thoughts and seeing them as just some of the many other experiences we may be having in a given moment. When we see our thoughts as thoughts, we neither believe or disbelieve them but instead simply notice that we can be aware of what our minds may be telling us.
Observer Self	Shifting our perspective to notice emotions and thoughts as things that we experience, but don't define us. Looking from the observer self allows us to connect to a part of ourselves that holds all our experiences, including moral pain.
Connecting to Values	Making the choice to connect to what matters most to us in a particular situation. This can include awareness of multiple values that are sometimes in tension with each other.
Living Values	Taking deliberate action to live our values. This can include taking concrete steps in our physical actions or practicing different ways of relating to our internal experience.

Session 2 Practice Exercise: Values Sort

Values Point the Way
Values are chosen qualities that, when lived, allow us to connect with meaning and purpose in our lives. Examples might be pursuing relationships with honesty, kindness, or fairness. A value is a like the needle on a compass. It points us in a general direction, even if it doesn't tell us exactly how to get there. When we live a value, we put a quality into action.

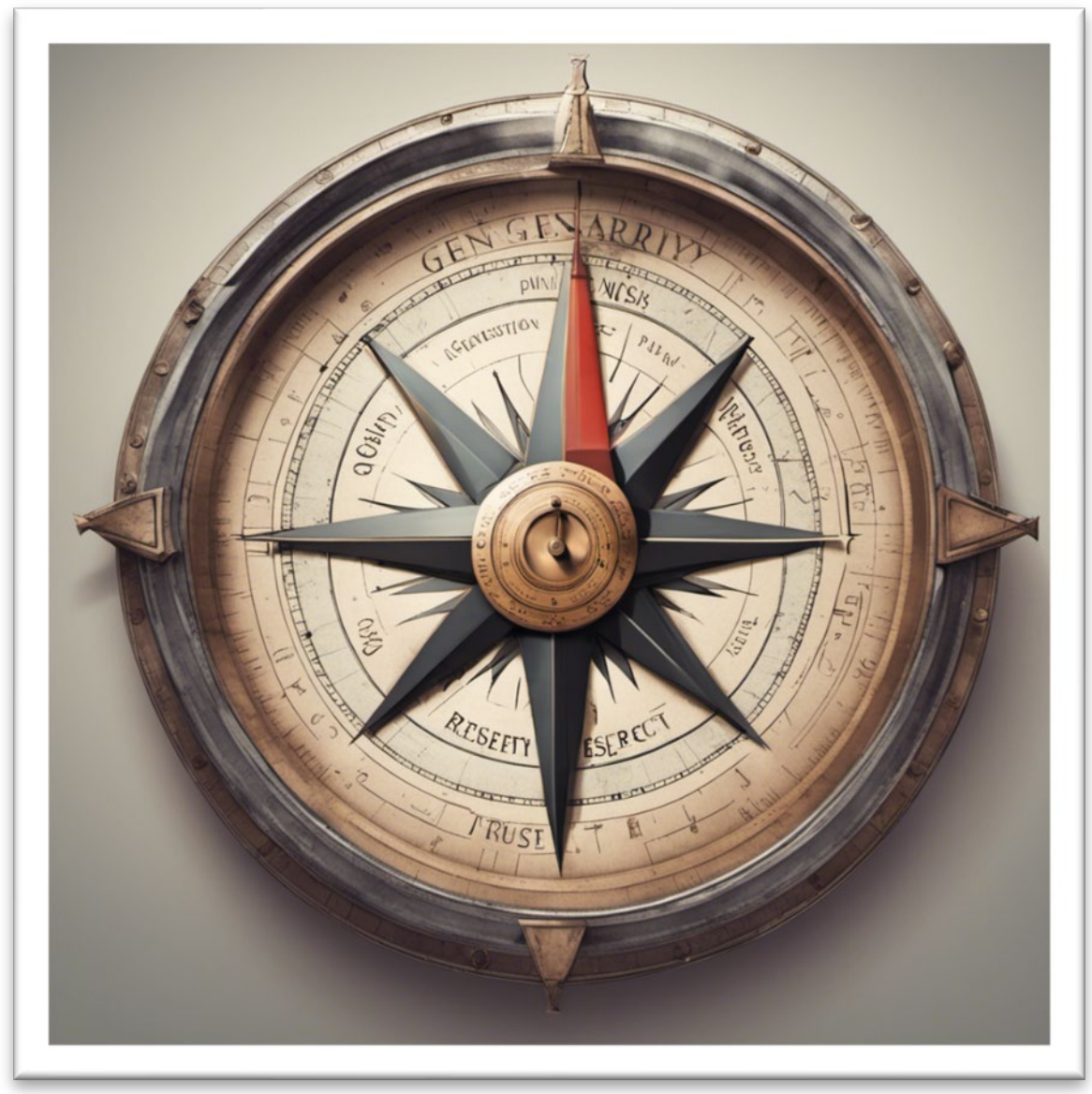

Before we can move in a direction we care about, we need to know what matters most to us. There are many values we might choose from. The values sort exercise is designed to help you identify which values are currently most important for you.

Values Sort Instructions

1. Review the values listed on the next page (a list of the values and their definitions are included)
2. In the blank rectangles you may add any values that are important to you but are not already included on the list of cards.
3. If using a printed version of this workbook, cut out all the values and category cards. If using an electronic version, proceed to step 5.
4. Shuffle the values cards so they are in random order.
5. One at a time, take a value and notice what degree of importance and meaning it has for you.
6. Place the value in the column that best fits how important or meaningful the value is to you personally (Less Important, Somewhat Important, Most Important). This exercise isn't about whether you are currently living a particular value. Instead, sort the value according to how much it matters to you.
7. Repeat this process until all values have been placed in a category. After you've sorted them all, feel free to review and re-sort them until you are satisfied.
8. Now take the values in the 'Most Important' pile and rearrange them in order from highest to lowest in terms of how important they are to you personally.
9. Pause and reflect on what it was like to complete this exercise by answering the questions in the Values Sort Review

Values Sort List

Adventure	Beauty	Caring for others
Citizenship	Compassion	Connectedness
Courage	Creativity	Curiosity/Learning
Discipline	Industrious	Efficiency
Equality	Physical Health	Forgiveness
Honorable	Independence	Integrity
Interdependence	Intimacy	Justice
Kindness	Leadership	Loyalty
Openness	Orderliness	Patience
Perseverance	Power	Reliable
Respectful	Security	Sensuality
Simplicity	Spirituality	Spontaneity
Stewardship	Structure	Tolerant
Understanding	Fill in your own	Fill in your own
Fill in your own	Fill in your own	Fill in your own
Fill in your own	Fill in your own	Fill in your own

Possible Value Definitions

The following list includes examples of potential values and their definitions

Value	Definition
**Adventurous**	Seeking new and exciting things
**Beauty**	Giving things the quality of being physically attractive
**Caring**	Doings things to keep others healthy and safe
**Citizenship**	Being a responsible member of a community
**Compassion**	Awareness of others' distress together with a desire to help
**Connectedness**	Investing in shared life experiences with others
**Courageous**	The ability to do something that you know is difficult
**Creative**	Making new things or thinking of new ideas
**Curious**	The desire to learn or know more about something or someone
**Disciplined**	A willingness to closely follow rules or orders
**Industrious**	Being constantly active and hard-working
**Efficient**	Producing desired results without wasting resources
**Equality**	Seeking for all to have the same rights and privileges
**Physically Healthy**	Seeking to be in good physical health
**Forgiving**	To stop blaming and allow room for error
**Honorable**	Showing honesty and good moral character
**Independent**	Being self-reliant for taking care of personal needs
**Integrity**	Being honest and fair with yourself and with others
**Interdependence**	Relying on one another to take care of needs
**Intimacy**	Being able to share something of a personal or private nature
**Justice**	Being unbiased, impartial, or fair
**Kindness**	Showing a gentle nature and a desire to help others
**Leadership**	The power or ability to lead other people
**Learning**	Gaining knowledge or skill through teaching or experience
**Loyalty**	Showing complete and constant support for someone or something
**Openness**	Willingness to share and receive genuine feelings and thoughts
**Orderliness**	Arranged or organized in a logical or regular way
**Patience**	Enduring challenges calmly or without complaint
**Perseverance**	To continue trying even though it is difficult
**Powerful**	The ability to control or influence people or things
**Reliable**	Able to be trusted to do or provide what is needed : able to be relied on
**Respectful**	Showing admiration for someone or something that is good, valuable, or important
**Security**	Doing things to make people or places safe
**Sensuality**	Devoted to producing enjoyment through the physical senses
**Simplicity**	Focusing on things that are ordinary but enjoyable
**Spirituality**	Given attention and concern to sacred matters
**Spontaneity**	Doing things in a natural and often sudden way and without much planning
**Stewardship**	Protecting and being responsible for something
**Structured**	Following a sequence, pattern or set of rules
**Tolerant**	Willing to accept feelings, habits, or beliefs that are different from your own
**Understanding**	Willing to see people's behavior from their perspective

Less Important	Somewhat Important	Most Important

Value Sort Review

1. List your top 3 most important values below:

1.	
2.	
3.	

2. Different values may be important for different areas of your life. Look at the areas below and, if they apply, list the values that may be especially important in these areas for you:

Area of Life	Important Value(s)
Family Relationships:	
Marriage/Significant Other:	
Friendships:	
Personal well-being:	
Employment:	
Spirituality:	
:	
:	

(If needed, add your own areas)

3. Write down what you noticed while looking through and sorting all of the values in this exercise. What thoughts, emotions, sensations, and urges did you notice when you considered particular values?

SESSION 3: DROPPING THE ROPE

Our Moral Emotions

When talking about moral pain and moral injury, we typically think about painful emotions like guilt, shame, embarrassment, anger, disgust, or contempt. We tend to feel these painful moral emotions when an experience we have conflicts with our personal values in some way. On the other hand, when we behave in a way that aligns with our values, we may feel emotions like pride, caring, gratitude or inspiration. We may even feel pleasant and painful moral emotions at the same time. After a morally injurious event, the experience of these moral emotions can be very intense and come with strong thoughts, physical sensations, and urges to act.

The Tug of War

When we try to avoid, control, or solve our moral pain, it's like being in a tug-of-war with a monster. You and the monster are positioned face to face, connected by a rope crossing a deep, dark pit. No matter how hard or how long you pull on the rope, the monster doesn't go away…it doesn't fall in the pit. What's more, pulling on the rope takes you away from the people and things that matter to you. How long have you been in your tug-of-war? What have been the costs of this struggle? What else could you be been doing with your time and energy if you didn't have to keep pulling on the rope? Does it feel important to stay in this tug-of-war? Why or why not?

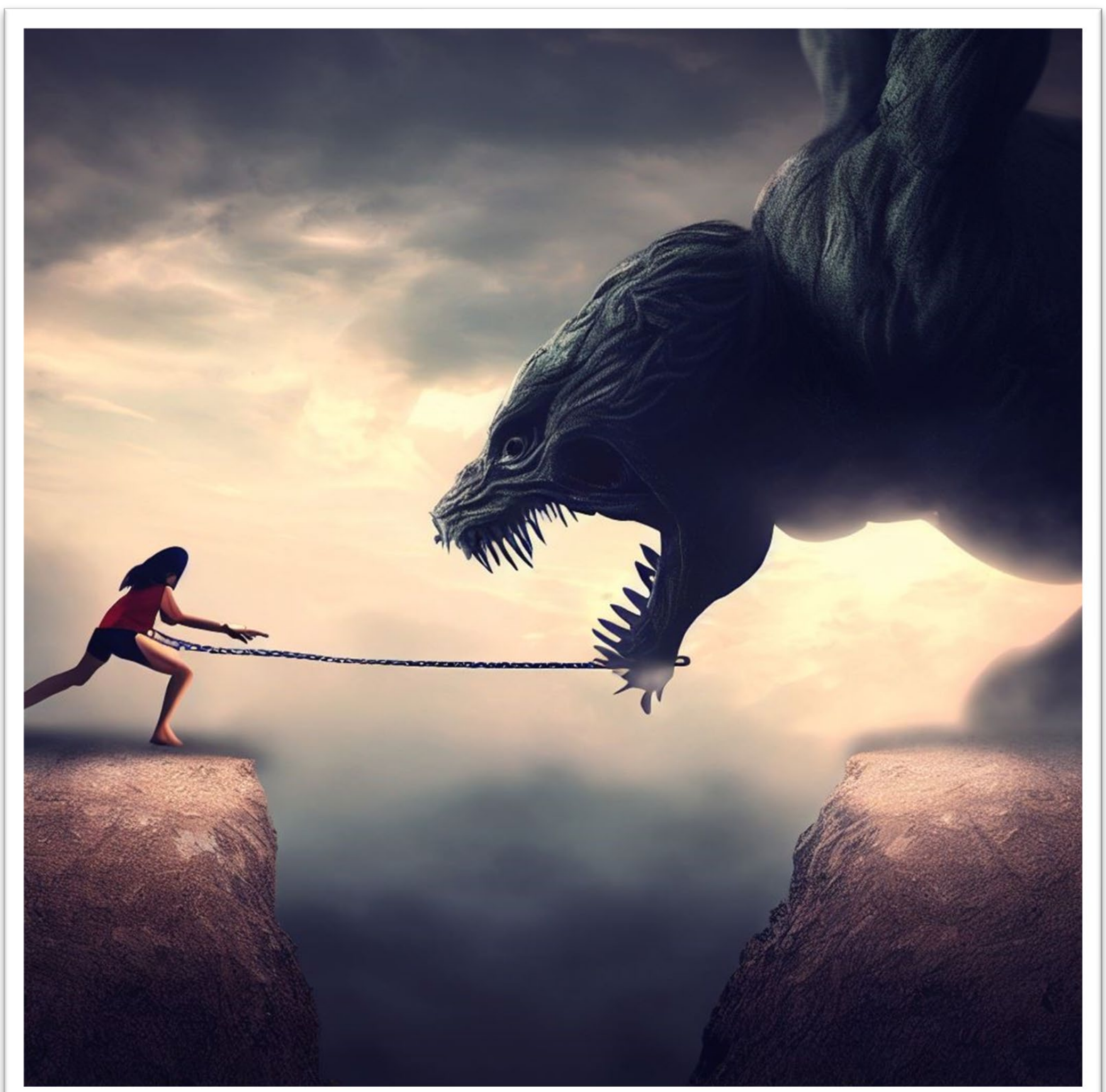

This figure shows some of the common pleasant and painful moral emotions and how each comes with thoughts, sensations and urges

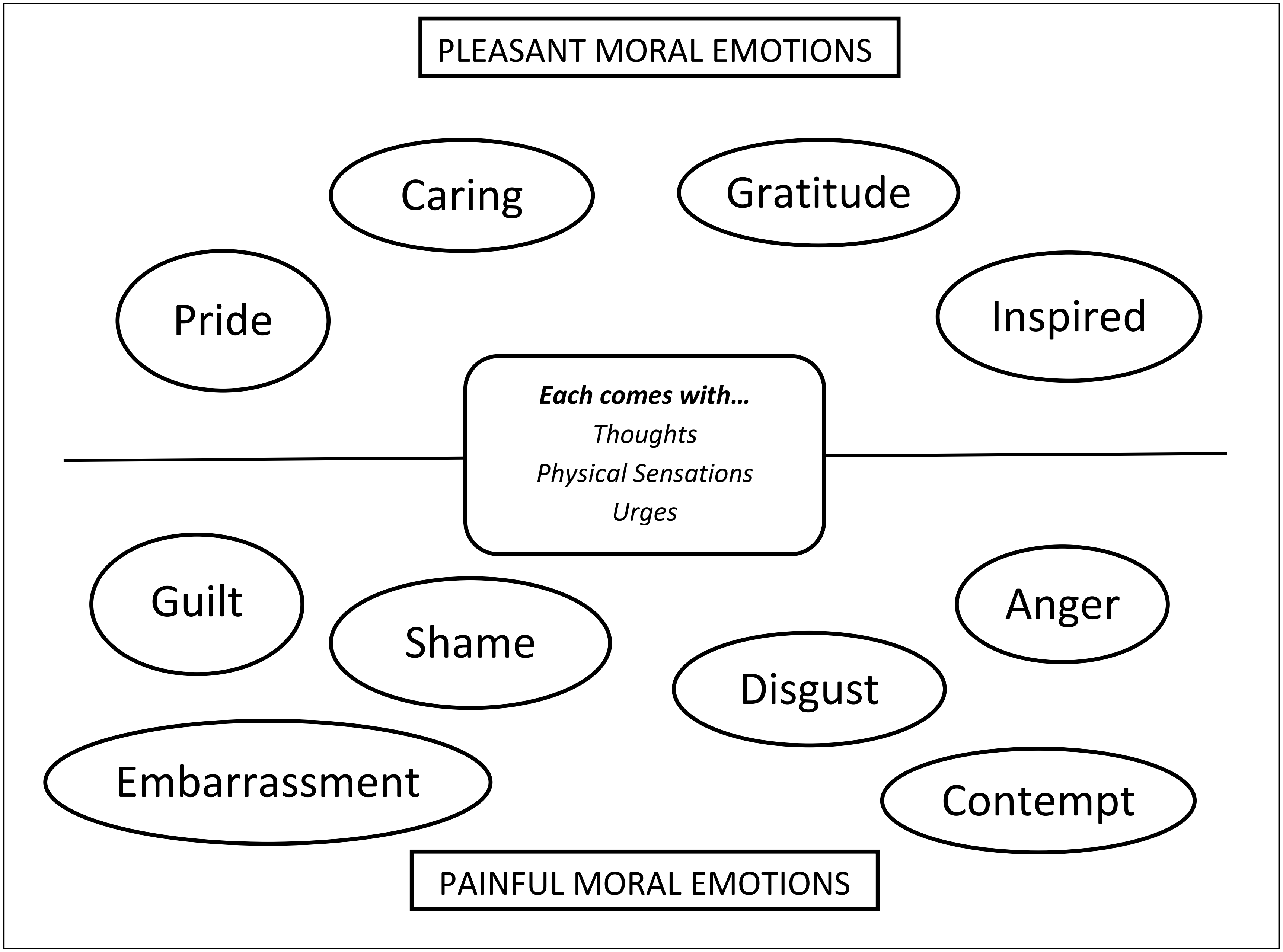

UNDERSTANDING MORAL EMOTIONS

This table names different moral emotions, how they might feel and the situations they might be triggered by.

	Emotions	Might be triggered by…	Might feel like…
**Pleasant Moral Emotions**	**Pride**	Behaving in a competent and virtuous way	Expansion of chest, confident and satisfied
	**Caring**	Encountering someone vulnerable or in need	Softening of the face, a desire to help, warmth
	**Gratitude**	Receiving support or assistance from others	Being friendly, an urge to return the favor
	**Inspired**	Witnessing virtue, generosity, or moral courage	Being uplifted, having a desire to improve
**Painful Moral Emotions**	**Embarrassed**	Losing the respect of others	Feeling flustered and confused
	**Shame**	Believing something is wrong with yourself	Urges to hide or isolate from others
	**Guilt**	Causing hurt or harm to those we care about	Sadness, a desire to repair the relationship
	**Anger**	Acts of unjustified insult or harm	Urges to protect, defend, or retaliate
	**Disgust**	Dehumanizing or degrading behavior	Urge to avoid/expel the source of disgust
	**Contempt**	Incompetent or inadequate behavior	Desire to ignore, exclude, or put down

Session 3 Practice Exercise: Seeing Possibilities

Return for a moment to the values you explored in Session 2. Reflect on the values that you wrote down in the workbook and then come back to this page.

It's not uncommon to feel confused about how to live your values. You may even feel like it is impossible to move forward with them because there are too many obstacles or because it's been so long you've forgotten what it looks like to live them. The process of getting unstuck can begin with a simple act of imagining.

Imagine for a brief moment that all your obstacles and barriers were removed, and you could act in any way that felt meaningful to you. What might you start doing? What steps could you take? How would those steps show up in your day-to-day life?

It's okay if opening up to those possibilities feels overwhelming. Try to stay present and imagine what a person might do if they chose to live those values. Then in the table below, write down those possibilities that seem meaningful to you. The possibilities don't have to be practical or even realistic for you in your current circumstances. This exercise is just to help us explore new possibilities for connecting to meaning.

	Top 3 Value	Possible Ways to Live the Value
1.		
2.		
3.		

As we noted before, different values may be important for different areas of your life. Return to the values you wrote down for the areas below in Session 2 and, just like above, imagine some possible ways those values might look if you were to live them in that particular area of your life. Write them in the table below.

Area of Life	Possible Ways to Live the Values
Family Relationships:	
Marriage/Significant Other:	
Friendships:	
Personal well-being:	
Employment:	
Spirituality:	
:	
:	

If you have trouble seeing possibilities, that's okay. Notice what comes up for you as you consider this and make note of it. It may help to look on the following page for some examples of possible ways to live values.

These tables include some examples of possible ways to live values.

Family (other than couples/parenting)	Marriage/Intimate Relationships	Parenting	Friends/Social Life	Work/Career	Education/Training
Call or text a loved one with whom I've lost touch<br><br>Invite a loved one to share how their day has gone<br><br>Show physical affection (ex. hug)	Spend time with my significant other<br><br>Share my feelings with significant other<br><br>Engage in physical intimacy with my significant other<br><br>Explore dating opportunities	Practice being in the present moment with my kids<br><br>Set aside time to listen to my children<br><br>Let kids choose an activity to do with me	Attend a social event<br><br>Invite others to do something together<br><br>Join a common interests group	Find something at work that matters and make it part of your day<br><br>Explore new job opportunities<br><br>Seek advancement in your career	Learn or practice a skill (e.g., wood working, painting)<br><br>Start a new area of learning and study<br><br>Research going back to school or taking community classes

Recreation/Fun	Spirituality/Religion	Citizenship/ Community Life	Physical Self-Care	Emotional Self-Care	Other:
Engage in a hobby<br><br>Do something spontaneous<br><br>Try something new	Spend time in nature<br><br>Contribute to others' welfare (service)<br><br>Personal devotions (prayer, meditation)<br><br>Attend a religious service of your choosing	Talk to your neighbors<br><br>Explore volunteer opportunities<br><br>Volunteer with a community organization	Eat healthy food<br><br>Exercise<br><br>Good sleep practices<br><br>Take medications as prescribed<br><br>Make a medical appointment	Spend money on yourself<br><br>Create specific time or space for yourself<br><br>Request help or support from others	

SESSION 4: HOLDING MORAL PAIN

Two Sides of a Coin
Moral pain is a healthy response when we experience something that conflicts with or departs from our values. In this way, our moral pain and our values are like two sides of the same coin. We feel pain because we care, and because we care, we open ourselves up to feel pain. The moral pain we feel is a signal for us to notice and pay attention to the values the pain is connected to.

Moral pain, like physical pain, can be very uncomfortable and distressing. So, it's understandable that when we feel moral pain, we might instinctively try to escape or ignore it by distracting ourselves or doing something to numb our emotions. These strategies might provide us some relief in the moment, but just like ignoring physical pain can result in greater injury to our bodies, avoiding or ignoring moral pain often leads us into greater emotional suffering. Without the pain to alert us to our values, we lose the feedback that would help guide us in our choices. In effect, by not allowing ourselves to feel moral pain, we lose touch with the parts of life that matter most to us.

Rather than avoiding moral pain, if we can learn to become an observer of our moral pain, sitting with it and gently holding it, we have space to create new possibilities. We can start to listen to it and better understand the value it is reminding us of. The skill of noticing and gently holding moral pain takes time and practice to learn.

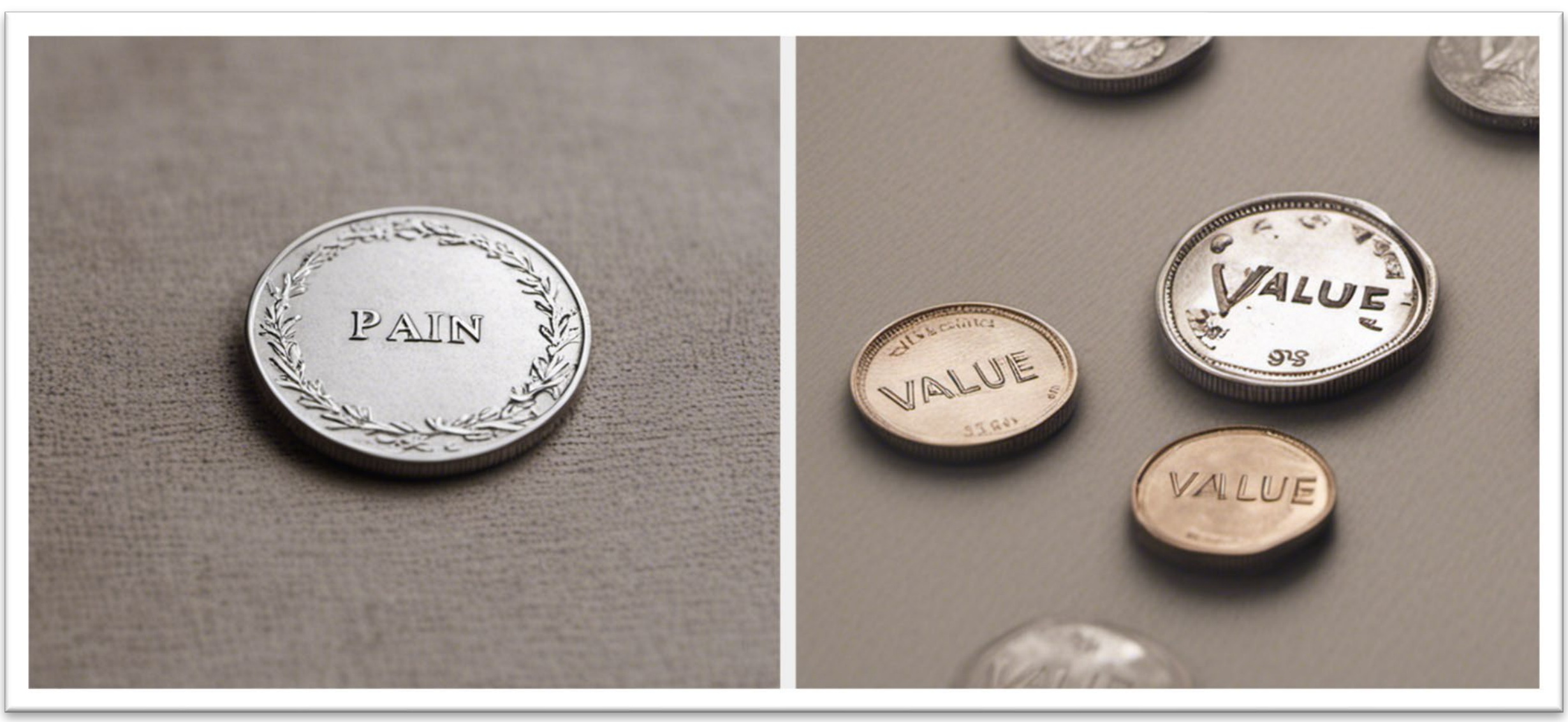

Session 4 Practice Exercises

Carry the Two-sided Paper

From now until the next session, carry the two-sided paper around with you. Carry it in your pocket, your shoe, take it out each day. Make space for each side of the paper. Set aside a few moments each day to intentionally turn your attention to the paper (If helpful, set reminders using a phone or a clock). Notice how you are carrying it. Take it out of your bag or pocket or shoe. Look at one side, then the other. Notice how the two sides are connected, how one side can't exist without the other. Notice any urges to avoid what is on the paper, and bring your attention to that content, notice how it feels in your hand, how you are holding it. Open your hand to both sides of the paper and allow it to be there with you.

Living Values

Return for a moment to the possibilities you explored in session 3. Reflect on the possibilities that you wrote down and the in the workbook and then come back to this page.

You're invited to continue taking steps in the service of living your values. What steps you take and when you take them is up to you but for change to happen, you will need to act.

Some opportunities to live our values are unplanned but others can be identified and planned out ahead of time. Planning out steps to live our values helps us practice ACT-MI skills and interrupt our patterns of avoidance. You're encouraged to plan out and take at least one step towards living your chosen values each day between ACT-MI sessions.

Using the worksheet on the following page can help you to plan and reflect back on the steps you took this week. The worksheet will ask you these questions that you can answer in boxes in the table:

- What step did you take to live your chosen values?
- Was your step planned or unplanned?
- What showed up inside you when you took the step?
- How did you choose to respond?
- What did you learn from the experience?

At the next group you'll be given an opportunity to share what steps you chose to take and your experience of living your values.

Use this table to plan and reflect back on taking steps to live a more meaningful and purposeful life before your next ACT-MI session.

	**What step did you take to live your values?**	**Was it planned or unplanned?**	**What showed up inside? (Thoughts, emotions, sensations, urges)**	**How did you choose to respond?**	**What did you learn?**
DAY 1					
DAY 2					
DAY 3					
DAY 4					
DAY 5					
DAY 6					

SESSION 5: THE OBSERVER YOU

The Place, Not the Pieces

We have been learning skills and perspectives that help us hold moral pain and orient ourselves towards our chosen values. Another powerful skill that can help us hold moral pain involves learning how to see from a perspective called "the observer-self." The observer-self is not an idea or an experience you can look at. Rather, the observer-self is a perspective that we can learn to see from. Another way to describe the observer-self is as the place where your internal experiences happen. It's the place where your thoughts, memories, emotions, sensations, and urges all show up. When we see our own experiences from the perspective of the observer-self, we are able to notice and hold them, without getting pulled off-course by attempts to control or avoid.

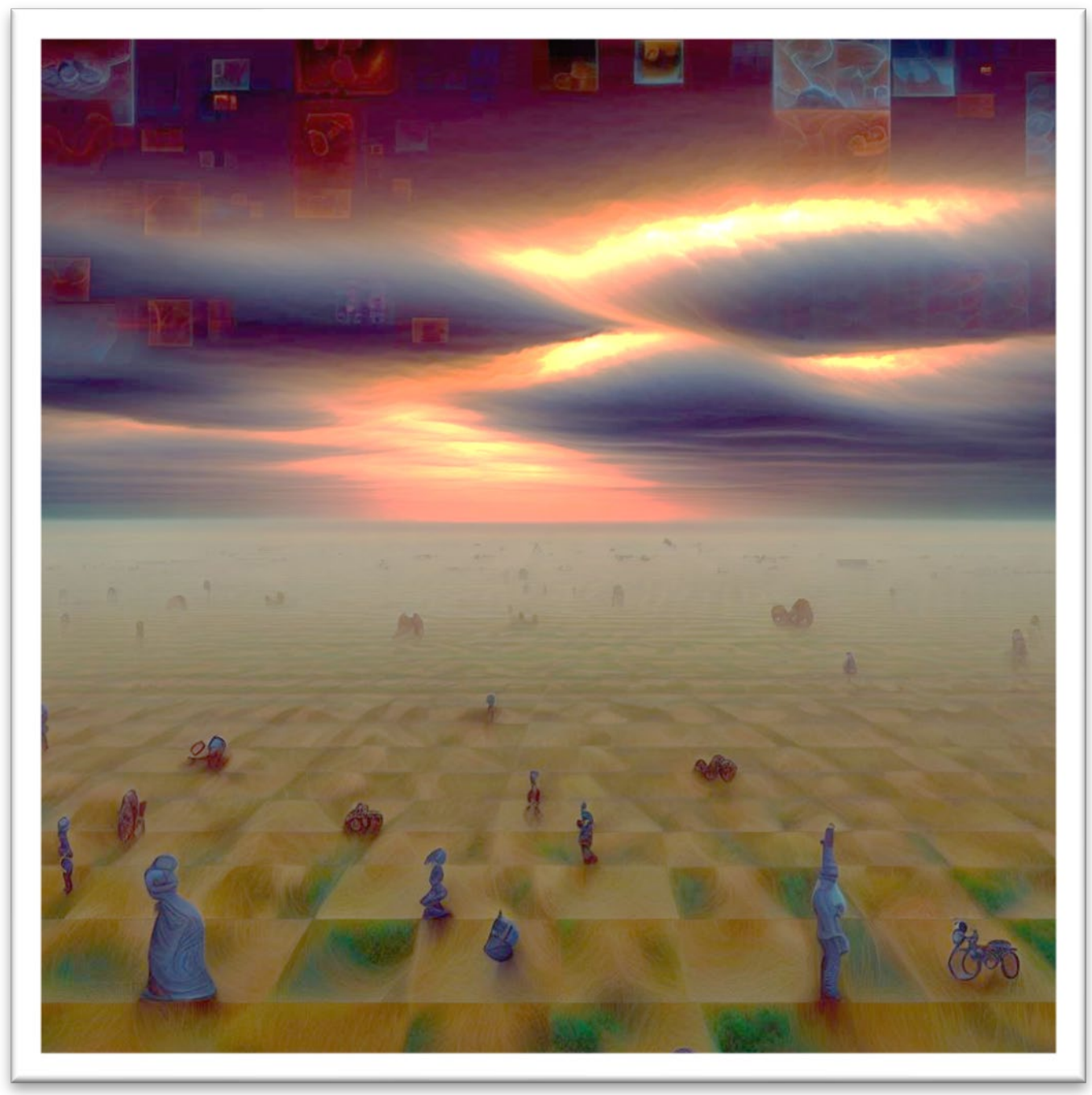

A way to help us understand the observer-self is to think of a chessboard that extends infinitely in all directions (if you prefer a different metaphor, an infinite sports field also works). On the chessboard are many chess pieces of different sizes and shapes. The pieces are in constant motion, including moving closer to or father away from any given spot on the board. The pieces touch the board, but they don't define it. The pieces come, they stay a while, and the move away, but the board itself remains unchanged. The board always holds all the pieces, no matter what form they take.

Our internal experience is like the chess pieces and the chessboard. The chess pieces are all your memories, emotions, thoughts, sensations, and urges, including both your painful and pleasant moral emotions. The chessboard is like the observer-self. It's the part of you that <u>holds</u> all your thoughts, emotions, sensations and urges. But because the observer-self holds your experiences, we know it can't be defined by them. The chessboard can instead be in contact with the pieces and allow them to move across its surface without the board itself being changed by the pieces. In the same way, as observer-selves, we can notice that we have thoughts, emotions and other experiences that move through our awareness, without changing the essence of who or what we are. When we practice seeing from the perspective of our observer-self, we're able to notice our thoughts and emotions and allow them to rise and fall without reaching out to grab them or push them away. We instead become an observer of the many and ever-shifting pieces of our own experience. What's more, when we take the role of an observer of our own experience, we can choose to redirect the time and energy we had been spending on fighting the pieces on our board and put that effort towards things that bring us greater meaning and purpose, such as being more present with and connected to the people and pursuits we care most about in life.

Session 5 Practice Exercises

Connecting to the Observer Self
We'd invite you to practice the skill of the Observer-Self in your relationships and other social interactions. Try the following exercise at least two times before the next group session.

Take a few minutes before an expected social interaction. For example, it could be before a meeting with a coworker, or before you walk into your home where a family member is inside, or before paying a cashier at a store. Bring the person you'll be interacting with into your mind and imagine what thoughts and emotions they might be having in that moment. Ask yourself, are those thoughts and emotions all of who they are? Notice that they aren't defined by those particular thoughts and emotions, but that, whether or not the person even realizes it, they have their own observer-self that is holding all the pieces of their experience.

Now notice your own experience… what thoughts and emotions are you having in this moment? Are you completely defined by those particular thoughts and emotions? Whatever your mind says in response to those questions, notice who is noticing what your mind is saying. See if you can recognize that no matter what thought, emotion, memory or experience you're having, that experience is distinct from the space that is holding it. That space that is holding the thought or emotion, is your observer-self.

Trying to stay in contact with the sense that both you and this other person are each more than their thoughts and emotions. Notice if using that perspective changes how you might interact with them. How might it provide you with new or more helpful ways of relating to each other? Notice also if it changes how you relate to your own experiences and if it opens new ways you might act towards yourself. Now move forward with your interaction, trying to stay present, open and aware.

Living Values
You're invited to continue taking steps in the service of living your values. What steps you take and when you take them is up to you but for change to happen, you will need to act.

Some opportunities to live our values are unplanned but others can be identified and planned out ahead of time. Planning out steps to live our values helps us practice ACT-MI skills and interrupt our patterns of avoidance. You're encouraged to plan out and take at least one step towards living your chosen values each day between ACT-MI sessions.

Using the worksheet on the following page can help you to plan and reflect back on the steps you took this week. The worksheet will ask you these questions that you can answer in boxes in the table:

- What step did you take to live your chosen values?
- Was your step planned or unplanned?
- What showed up inside you when you took the step?
- How did you choose to respond?
- What did you learn from the experience?

At the next group you'll be given an opportunity to share what steps you chose to take and your experience of living your values.

Use this table to plan and reflect back on taking steps to live a more meaningful and purposeful life before your next ACT-MI session.

	What step did you take to live your values?	Was it planned or unplanned?	What showed up inside? (Thoughts, emotions, sensations, urges)	How did you choose to respond?	What do you want to learn from this experience?
DAY 1					
DAY 2					
DAY 3					
DAY 4					
DAY 5					
DAY 6					

SESSION 6: CARRYING MORAL PAIN

How We Hold Moral Pain

A common response to moral pain is to try to escape it. We might try to push it away or numb it. If we're ashamed of the pain, we might try to hide it from others. The urge to control our own moral pain is often so familiar and happens so fast that we often don't notice it until we are already acting it out. However, the more we try to avoid moral pain, the more we are pulled away from living our chosen values, in turn creating more moral pain. Avoiding becomes an ever-expanding cycle of moral injury .

With intentional practice over time, we can learn to hold our moral pain gently and with openness. This doesn't mean that the pain itself will change. **Instead, what changes is how we are choosing to relate to our moral pain.** We can use our own hands can demonstrate different ways of holding our moral pain. If you were holding your moral pain in your palm, would your hand be gripping it tightly? Would your hand be pushing it away or trying to drop it? Now, imagine instead that your hand is palm up, open, and relaxed, with the moral pain gently resting on its surface. This way of holding moral pain is what we mean by acceptance. It is not condoning or agree with what happened. It is allowing your present experience to be, just as it is.

Opening up to our moral pain in this way allows new possibilities for living that don't revolve around avoiding or controlling your unwanted experiences. It creates the opportunity to do things that matter to you. Holding moral pain gently and with openness also allows us to hold the moral pain of others and allow our moral pain to be held by them in return. Holding moral pain together, if done with care, trust and respect, allows us to show and be shown compassion and is a powerful way to honor our values and the connections that exist with those around us.

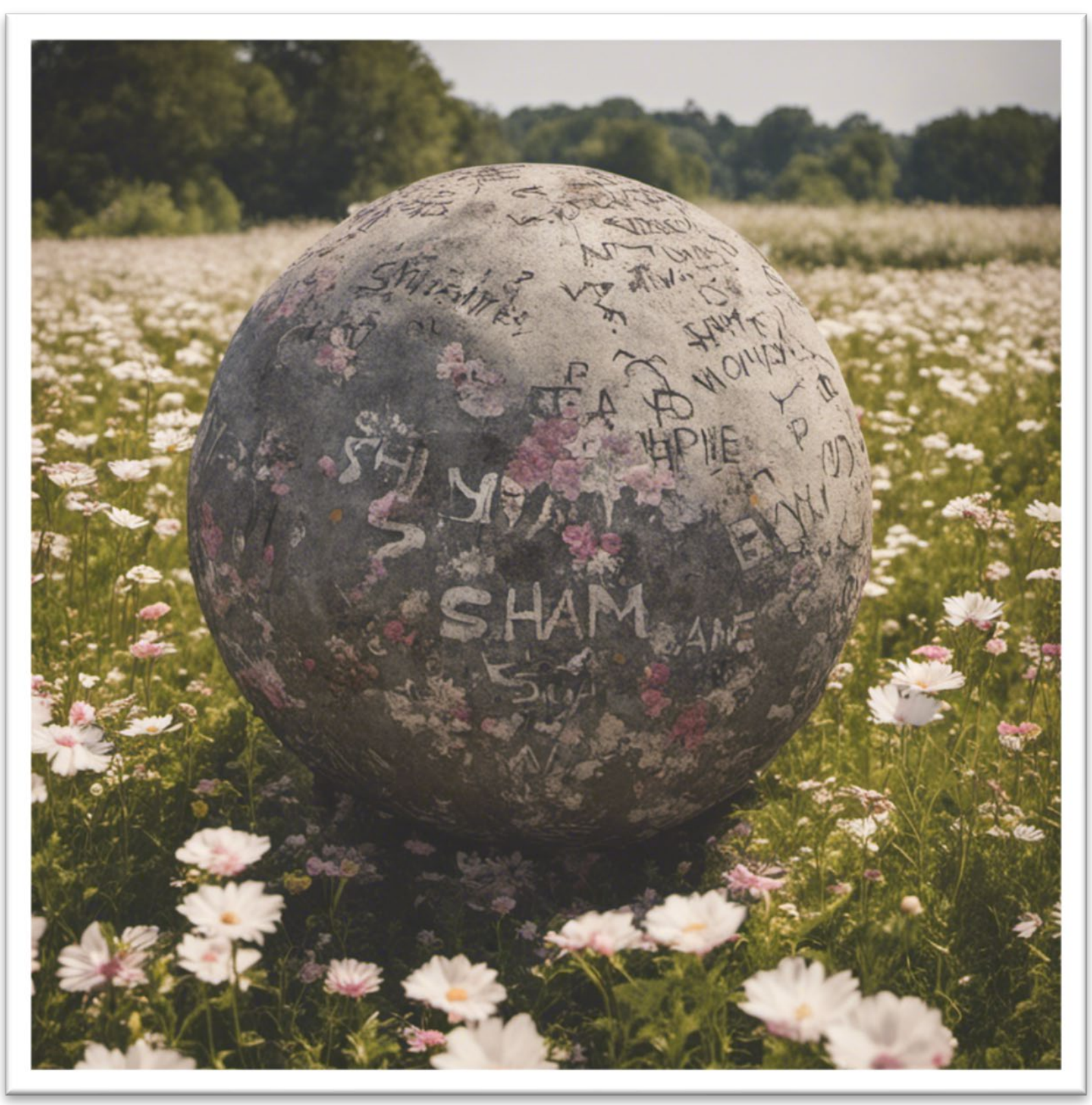

Session 6 Practice Exercises

The Timeline

On the next page is a dotted line. The line represents your time alive on earth, starting at your birth and moving through the present moment.

Above or below the dotted line, draw dots to indicate morally pleasant and morally painful events you've experienced. Examples of pleasant events might be getting married, the birth of children, or military promotions. Examples of painful events would be morally injurious experiences that happened before, during or after your military service. The distance of the dot from the dotted line indicates the intensity of the experience, with farther distance meaning more intensity. If an event was both pleasant and painful, instead of a dot, draw a vertical bar that extends above and below the dotted line.

As part of ACT-MI, you've already identified some examples of your morally painful events. To help recall examples of morally pleasant events from your past, you might consider times when…

… someone did something that changed the course of your life for the better.

… you were able to help someone else who needed you.

… you achieved something that had felt meaningful but out of reach.

… you witnessed something that made you want to live your values more fully.

Next to each dot you draw, write the approximate date or year of the event and a few words that described what you were feeling during or immediately after the event.

After you're done adding events to the page, pause and reflect on the timeline you've created. What stands out to you? Are there parts left blank or parts with lots of events? What thoughts or emotions are you having as you look at the timeline? You can write down what you notice in the box below.

Use this page to complete the Timeline exercise

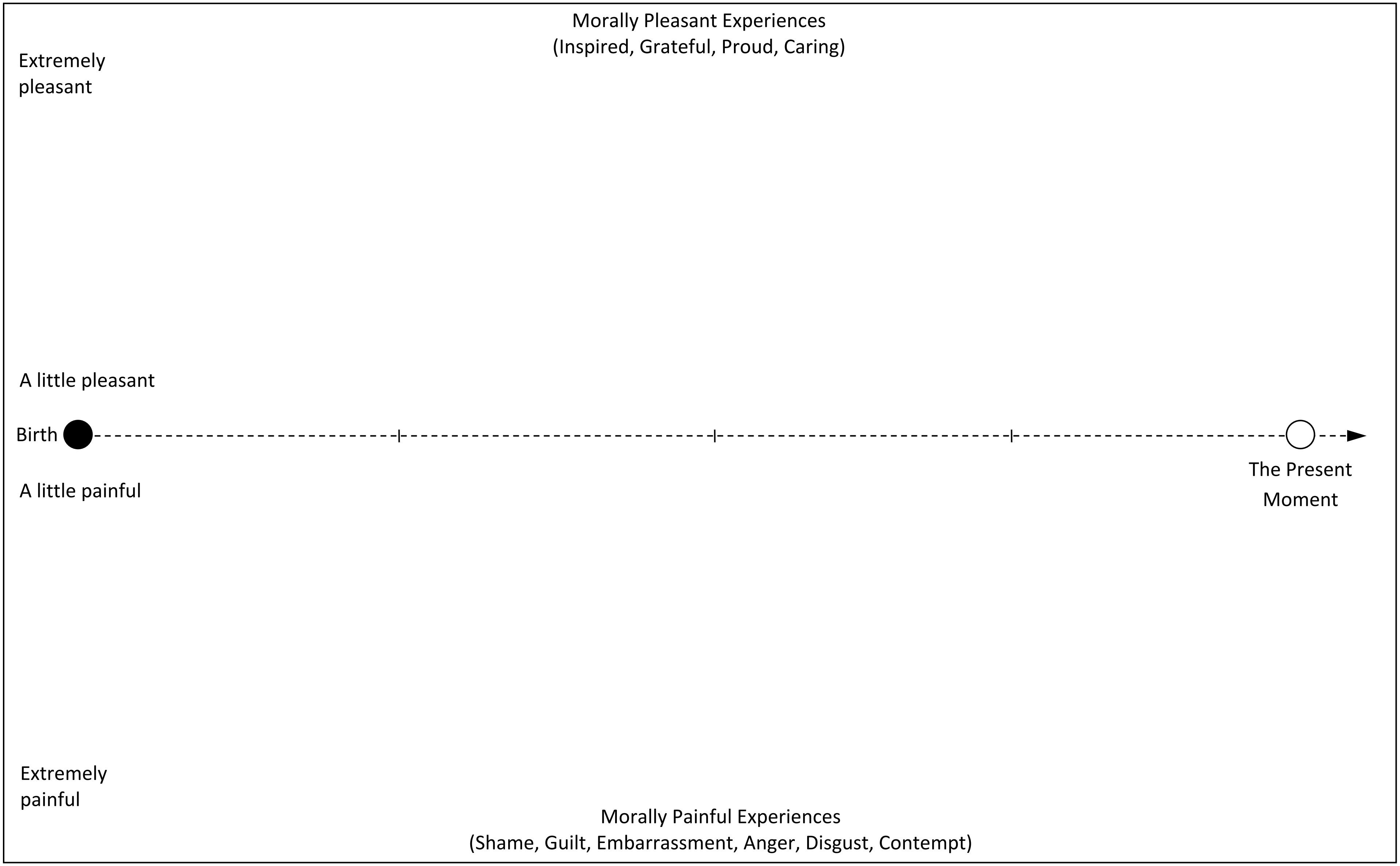

Living Values

You're invited to continue taking steps in the service of living your values. What steps you take and when you take them is up to you but for change to happen, you will need to act.

Some opportunities to live our values are unplanned but others can be identified and planned out ahead of time. Planning out steps to live our values helps us practice ACT-MI skills and interrupt our patterns of avoidance. You're encouraged to plan out and take at least one step towards living your chosen values each day between ACT-MI sessions.

Using the worksheet on the following page can help you to plan and reflect back on the steps you took this week. The worksheet will ask you these questions that you can answer in boxes in the table:

- What step did you take to live your chosen values?
- Was your step planned or unplanned?
- What showed up inside you when you took the step?
- How did you choose to respond?
- What did you learn from the experience?

At the next group you'll be given an opportunity to share what steps you chose to take and your experience of living your values.

Use this table to plan and reflect back on taking steps to live a more meaningful and purposeful life before your next ACT-MI session.

	What step did you take to live your values?	Was it planned or unplanned?	What showed up inside? (Thoughts, emotions, sensations, urges)	How did you choose to respond?	What did you learn?
DAY 1					
DAY 2					
DAY 3					
DAY 4					
DAY 5					
DAY 6					

SESSION 7: DISRUPTING MORAL INJURY

Over the past several sessions, we've practiced skills that can help us notice and hold moral pain without getting stuck in or controlled by it. We can use the skill of chain analysis (see session 1) to apply these skills and concepts in situations where we still feel stuck in response to moral pain to get some ideas about how to break patterns of moral injury.
As reminder a chain analysis involves…

1) Picking a behavior that we want to understand better (we call this the "target behavior").
2) Asking yourself, "What triggered the target behavior this time?"
3) Exploring your experiences and behavior between the trigger and the target behavior including what you were doing, feeling, and thinking. Each link in the chain can help us better understand why the target behavior keeps occurring and options you have for choosing different behavior.
4) Connecting with what you were feeling, thinking, and experiencing ***right before*** the target behavior
5) Paying attention to how your emotions, thoughts, sensations, and urges immediately changed during and after the target behavior. Was there a slight or big change in your experience **during and right afte**r the target behavior? Did the behavior change other people's responses to you in the moment?
6) Reflecting on whether your target behavior had any long-term impacts on living your values. For example, did it help you live your values more fully or did it turn you away from them?

<u>Now add the following step…</u>

7) Look back over the links in the chain and explore places where the ACT-MI skills covered so far in our sessions might have changed how this situation unfolded. For example, you might ask yourself these questions:

ACT-MI SKILL	QUESTION
Being Present	Could I have slowed down somewhere and noticed what was happening?
Accepting (Drop the rope)	Were there emotions, thoughts, or other experiences I could have opened up to and allowed to be in that moment?
Seeing thoughts as thoughts	Were there any thoughts that I might have unhooked myself from?
Observer Self (Holding pain)	When might I have shifted to notice my experiences as pieces I hold (like the chessboard)?
Connecting to what matters	Where could I have connected to what mattered to me in the situation?
Living Values	What step could I have taken to do something meaningful?

Session 7 Practice Exercise
You're invited to practice chain analyses on your own by following the instructions on the blank Chain Analysis Worksheet included in this section of the workbook. You can pick any behavior you want to better understand and do a chain analysis on it. Looks for places to use your ACT-MI skills in your chain so that you can break it and choose to live your values in any situation even with the pain you are experiencing. Come ready to share your chain next session.

Breaking Patterns of Moral Injury Worksheet

Complete this worksheet the same way you did in session 1, but this time, also identify ways to break the pattern of moral injury by inserting ACT-MI skills between the links in the chain (see next page for more instructions)

1: Target Behavior

2: What **triggered** the target behavior *this time*?

3.

4.

5.

6.

7.

8.

9. (Just before the behavior)

10: What happened after to **my** thoughts, emotions, sensations, urges, memories?

11: How did **other people** respond or my surroundings change right after?

12: What are the **long-term impacts** of the target behavior?

Breaking Patterns of Moral Injury Worksheet

Step A: Write down in **Circle 1** something you did in response to feeling shame, guilt, anger, disgust, or contempt that would be helpful to understand.
Step B: What triggered the target behavior **this time**? Write this in the box labeled **2: What happened that triggered…**
Step C: How did you get from the **trigger in circle #2** to the **target behavior**? Fill circles **3-9** with the thoughts, emotions, sensations, urges, behaviors, or actions that happened in between the trigger **(Circle 2)** and the behavior you're trying to understand better **(Circle 1)**.
Step D: What happened to your *internal experiences* immediately after the **target behavior**? Write this in **Box 10**. (Rate the intensity from 0-100)
What happened to your *external experiences* immediately after the **target behavior**? Write this in **Box 11**. (Rate the intensity from 0-100)
Step E: What are the long-term impacts of the **target behavior**? Write these in **Box 12.**
Step F: Review the ACT-MI skills listed below and list places in the chain where they could be used to interrupt the pattern of moral injury.

ACT-MI SKILL	DESCRIPTION
**Being Present**	Slowing down and noticing what is happening in the present moment. Connecting with the here and now and all that the present contains.
**Accepting (Dropping the rope)**	Opening up to and allowing emotions, thoughts, or other internal experiences to be in this moment, just as they are. This does not mean enjoying the experiences or condone the events they are connected to. Rather, making a choice to acknowledge and stop fighting against the reality of the events and the experiences connected to them.
**Seeing thoughts as thoughts**	Disentangling from thoughts and seeing them as just some of the many other experiences you may be having in a given moment. When you see your thoughts as thoughts, you neither believe or disbelieve them, but simply notice that you can be aware of what your mind may be telling you.
**Observer Self (Holding pain)**	Shifting your perspective to notice emotions and thoughts as things that we experience, but don't define us. Looking from the observer self allows us to connect to a part of ourselves that holds all our experiences, including moral pain.
**Connecting to what matters**	Connecting to what matters most to you in a particular situation and noticing what value(s) your pain might be pointing to (values/pain two sided paper exercise). This might include awareness of multiple values that might all be showing up in a particular moment.
**Living Values**	Taking deliberate action to live in a meaningful way. This can include concrete behaviors in the direction of values through physical actions or practicing different ways of relating to your internal experience.

Session 8 Practice Exercises

The You Across Time

Being able to connect with and look from the observer-self is a skill that takes time and practice to develop. As you become more familiar with your observer-self, you can notice that as you look back across your life experiences, whether they happened one second, one month or one decade ago, that in those moments too you were present as an observer. This was and is true even if you weren't aware at the time that your observer-self existed or if you didn't have the language to describe it. In the here and now, you can practice connecting with your observer self that extends from the current moment all the way into your past. As one way to do this, spend time this week reviewing pictures of yourself from different periods of your life. If you don't have any pictures available, you can reflect on memories from different points across your life or hold a physical object that has a connection to your past. While maintaining your awareness of yourself in the here and now, notice how even though many things may have changed in your life across time, there is a You that has be present for all of them. A conscious space that has held and continues to hold all of those constantly changing and flowing experiences. This space is the observer-self and can be connected to anytime you notice that you are the one doing the noticing. With time and practice, you will more quickly and effectively connect with the observer-self and use the freedom this perspective provides to live with meaning and purpose in the here and now.

Living Values

You're invited to continue taking steps in the service of living your values. What steps you take and when you take them is up to you but for change to happen, you will need to act.

Some opportunities to live our values are unplanned but others can be identified and planned out ahead of time. Planning out steps to live our values helps us practice ACT-MI skills and interrupt our patterns of avoidance. You're encouraged to plan out and take at least one step towards living your chosen values each day between ACT-MI sessions.

Using the worksheet on the following page can help you to plan and reflect back on the steps you took this week. The worksheet will ask you these questions that you can answer in boxes in the table:

- What step did you take to live your chosen values?
- Was your step planned or unplanned?
- What showed up inside you when you took the step?
- How did you choose to respond?
- What did you learn from the experience?

At the next group you'll be given an opportunity to share what steps you chose to take and your experience of living your values.

Use this table to plan and record taking steps to live a more meaningful and purposeful life before your next ACT-MI session.

	What step did you take to live your values?	Was it planned or unplanned?	What showed up inside? (Thoughts, emotions, sensations, urges)	How did you choose to respond?	What did you learn?
DAY 1					
DAY 2					
DAY 3					
DAY 4					
DAY 5					
DAY 6					

Session 9 Practice Exercises

You Are More than Your Moral Injury

This coming week, you're invited to set aside time to intentionally revisit the memory or memories of your morally injurious experiences. The purpose of this exercise is to practice connecting to your observer-self so you can remain free to choose even while experiencing painful memories and the thoughts and emotions that accompany them. When you find yourself getting caught in the memory of your morally injurious event(s) this coming week, practice pausing and noticing that it was <u>you</u> that had become wrapped up in the memory. That Observer-You that became caught in the memory for that moment always has and always will hold the memory, and so is separate from and bigger than the memory itself.

Here are some questions you can ask yourself now or in the future when you find yourself getting stuck in a memory or other painful experience:

- Notice, who is the one having this thought, emotion or memory?
- After you notice a thought in your mind, ask yourself "Who just said that?"
- Imagine a loved one is telling you they are having this painful thought, how would you want to hold their experience? Try to hold your own experience the same care and compassion.
- Ask yourself, what will it cost to myself or others I care about if I keep struggling in this way with these thoughts, emotions or memories?

Living Values

You're invited to continue taking steps in the service of living your values. What steps you take and when you take them is up to you but for change to happen, you will need to act.

Some opportunities to live our values are unplanned but others can be identified and planned out ahead of time. Planning out steps to live our values helps us practice ACT-MI skills and interrupt our patterns of avoidance. You're encouraged to plan out and take at least one step towards living your chosen values each day between ACT-MI sessions.

Using the worksheet on the following page can help you to plan and reflect back on the steps you took this week. The worksheet will ask you these questions that you can answer in boxes in the table:

- What step did you take to live your chosen values?
- Was your step planned or unplanned?
- What showed up inside you when you took the step?
- How did you choose to respond?
- What did you learn from the experience?

At the next group you'll be given an opportunity to share what steps you chose to take and your experience of living your values.

Use this table to plan and reflect back on taking steps to live a more meaningful and purposeful life before your next ACT-MI session.

	What steps did you take to live your values?	Was it planned or unplanned?	What showed up inside? (Thoughts, emotions, sensations, urges)	What did you learn?	What will you try next?
DAY 1					
DAY 2					
DAY 3					
DAY 4					
DAY 5					
DAY 6					

SESSION 10: SWEET SPOTS

You have all kinds of thoughts, emotions, urges and memories, some memories you may wish you didn't have (like the morally injurious event), and some that are sweet and cherished. While it's important to learn how to hold our painful experiences, it's also important to make space from our pleasant experiences as well and for what they might signal matters to you. We can do this in the same way we've learned to hold our moral pain without becoming that pain. By making room for all your experiences that have happened throughout your lifetime we can notice that we are not defined by any one event, and that all these experiences have something important to tell us. Our cherished memories and the pleasant moral emotions we feel in connection to them, can tell us when in our lives a value of ours was lived well and give us a guide for what might matter to us going forward. All these memories hold different "messages" for you, and given that none can be undone, the way forward is in choosing which messages you will act on. Remember, you are more than any memory, past action, thought, feeling, sensation. You are the one who carries all your memories, and with each step you take moving forward, you make a choice what you want your life to be about.

Session 10 Practice Exercises

Living Values
You're invited to continue taking steps in the service of living your values. What steps you take and when you take them is up to you but for change to happen, you will need to act.

Some opportunities to live our values are unplanned but others can be identified and planned out ahead of time. Planning out steps to live our values helps us practice ACT-MI skills and interrupt our patterns of avoidance. You're encouraged to plan out and take at least one step towards living your chosen values each day between ACT-MI sessions.

Using the worksheet on the following page can help you to plan and reflect back on the steps you took this week. The worksheet will ask you these questions that you can answer in boxes in the table:

- What step did you take to live your chosen values?
- Was your step planned or unplanned?
- What showed up inside you when you took the step?
- How did you choose to respond?
- What did you learn from the experience?

At the next group you'll be given an opportunity to share what steps you chose to take and your experience of living your values.

Use this table to plan and reflect back on taking steps to live a more meaningful and purposeful life before your next ACT-MI session.

	What steps did you take to live your values?	Was it planned or unplanned?	What showed up inside? (Thoughts, emotions, sensations, urges)	What did you learn?	What will you try next?
DAY 1					
DAY 2					
DAY 3					
DAY 4					
DAY 5					
DAY 6					

SESSION 11: EMBRACING CHOICE

Each of us are making choices every moment of every day. We choose how to spend our time, what words to say, what to pay attention to, where we go and how we act when we get there. Unfortunately, we make many of these choices without realizing that we are the ones doing the choosing. The choice might be made so quickly or without awareness that it feels we don't have control over it. If it's a choice we've made many, many times in the past, we might even call the choice a habit. If we are experiencing strong feelings or urges, our mind might tell us that we don't have a choice and that we must act in a certain way to get out of the feeling. In all these situations, we are still making choices, but our freedom to choose among the full range of our options is restricted because we are unwilling to stay in the moment. When we run away from the present, we also run away from our ability to choose differently than we have in the past. Being willing to stay in contact with the present moment is crucial to embracing our freedom to choose because choice only exists in the here and now. We cannot make new choices in the past, and we can't make future choices until those future moments arrives for us in the present. The only place we have any ability to choose is in the here and now.

If we slow down and open ourselves up to the present moment, we will find that each moment contains many different experiences, both pleasant and painful. We then have a choice to make about sitting with and gently holding all of those experiences as an observer. When we practice these skills, we become able to choose for ourselves instead of our minds choosing for us. In short, when we stop trying to be free of moral pain, we then become immediately free to live meaningfully and purposefully in the pursuit of what matters most to us.

Session 11 Practice Exercises (cont.)

Living Values

You're invited to continue taking steps in the service of living your values. What steps you take and when you take them is up to you but for change to happen, you will need to act.

Some opportunities to live our values are unplanned but others can be identified and planned out ahead of time. Planning out steps to live our values helps us practice ACT-MI skills and interrupt our patterns of avoidance. You're encouraged to plan out and take at least one step towards living your chosen values each day between ACT-MI sessions.

Using the worksheet on the following page can help you to plan and reflect back on the steps you took this week. The worksheet will ask you these questions that you can answer in boxes in the table:

- What step did you take to live your chosen values?
- Was your step planned or unplanned?
- What showed up inside you when you took the step?
- How did you choose to respond?
- What did you learn from the experience?

At the next group you'll be given an opportunity to share what steps you chose to take and your experience of living your values.

Use this table to plan and reflect back on steps to live a more meaningful and purposeful life before your next ACT-MI session.

	What steps did you take to live your values?	Was it planned or unplanned?	What showed up inside? (Thoughts, emotions, sensations, urges)	What did you learn?	What will you try next?
DAY 1					
DAY 2					
DAY 3					
DAY 4					
DAY 5					
DAY 6					

SESSION 12: LOOKING FORWARD

Much of the work in ACT-MI this far has been about learning how to hold our past by looking from the perspective of the observer self. Your observer self has existed across time. It was there when you were born, and it was there as you grew up. It's the part of you that's been there observing all your pleasant and painful experiences as they occurred across time, including your moral injury. Your observer self has been a constant and steady presence across all those events and experiences.

Just as we can look from the observer self across our past, we can also use the perspective of the observer self to look into our possible futures. The goal of doing this isn't to perfectly predict what's going to happen since none of us can know that. Instead, the goal is to hold in our awareness the possibilities available to you going forward and what actions you might take to start moving toward the possible futures that matter to you. It's also important to be aware that there's one common reality that we'll all face, and that is that our time in this life is limited. Your time is precious, and that's why it's important to get in touch with how you want to spend the time you have left – so you can choose how you live and what you leave for the people who matter to you and the world.

Session 12 Practice Exercises

Timeline Exercise - Part II

The Timeline Exercise in session 6 focused on our lives up until the present moment. This second part of the timeline exercise focuses on our lives from the present moment going forward. None of us knows how much longer we have left in our lives or exactly what events will occur during that time, but we do know that while we are alive, we will be having experiences that may include pleasant or painful feelings and that we will make choices about how to respond to those experiences.

For part II of the Timeline Exercise look at the figure on the following page and reflect on the following questions:

- What thoughts and feelings come up for you as you look at the time that lies ahead?
- What choices will make this time meaningful for you and those you care about?
- What future events (pleasant or painful) can you reasonably foresee? (add dots for these on the next page)
- How do you plan to respond to the morally painful events that will come your way?
- How do you plan to respond to the morally pleasant events that will happen?
- What words do you want to describe some of the future meaningful acts you will make? What values will guide those actions?

After you've had a moment to reflect, write down in the box below some of the answers that feel meaningful to you.

Use this page to complete part II of the Timeline Exercise

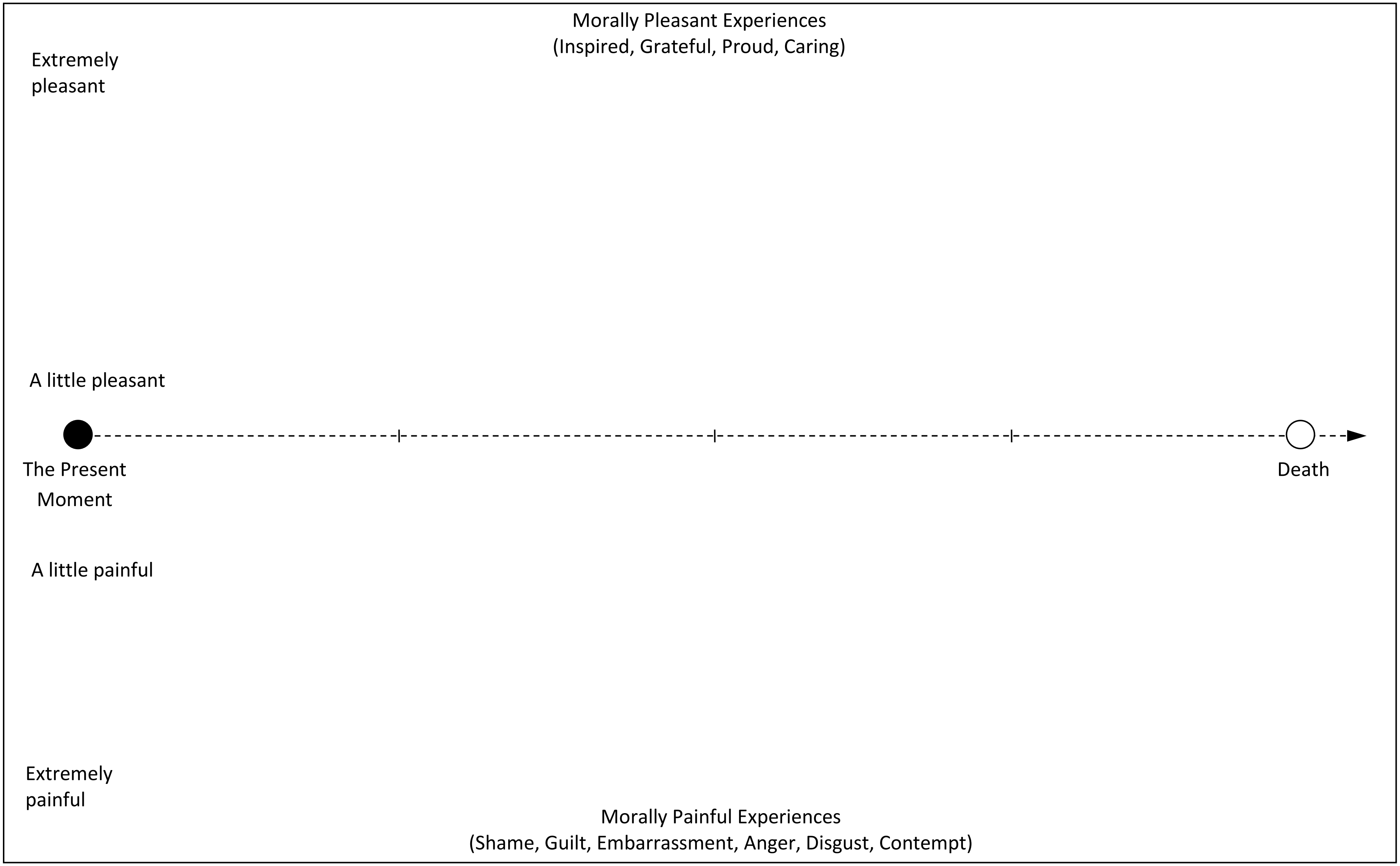

Session 12 Practice Exercises (Cont.)

Living Values

You're invited to continue taking steps in the service of living your values. What steps you take and when you take them is up to you but for change to happen, you will need to act.

Some opportunities to live our values are unplanned but others can be identified and planned out ahead of time. Planning out steps to live our values helps us practice ACT-MI skills and interrupt our patterns of avoidance. You're encouraged to plan out and take at least one step towards living your chosen values each day between ACT-MI sessions.

Using the worksheet on the following page can help you to plan and reflect back on the steps you took this week. The worksheet will ask you these questions that you can answer in boxes in the table:

- What step did you take to live your chosen values?
- Was your step planned or unplanned?
- What showed up inside you when you took the step?
- How did you choose to respond?
- What did you learn from the experience?

At the next group you'll be given an opportunity to share what steps you chose to take and your experience of living your values.

Use this table to plan and reflect back on taking steps to live a more meaningful and purposeful life before your next ACT-MI session.

	What steps did you take to live your values?	Was it planned or unplanned?	What showed up inside? (Thoughts, emotions, sensations, urges)	What did you learn?	What will you try next?
DAY 1					
DAY 2					
DAY 3					
DAY 4					
DAY 5					
DAY 6					

SESSION 13: THE SKILL OF RETURNING

One of the most important skills in ACT-MI is that of choosing to return. Returning means making the choice to come back to the present moment, to connect with the observer self that can see and not be one's experiences. It means returning to the values we care about and exploring new ways of connecting to meaning regardless of how we lived the day (or minute) before. As humans we all slip into old familiar patterns of behavior, or we may have been moving so quickly we missed an opportunity to pause and choose to act in a meaningful way. When we find ourselves veering away from our chosen values, we can return to the skills we have learned in ACT-MI and make a choice in the here and now to re-align our actions with those things that bring us fulfillment and purpose. In addition to reviewing the previous sections in this workbook, below are some questions and prompts that may help you practice returning to these skills.

Being Present

- Slow down and let yourself connect with the present moment. Become aware of what is happening within and around you without trying to change any of it.
- When you find yourself carried away in a thought, feeling or memory, gently acknowledge it and return to the present moment. Sometimes it can be helpful to pick a cue in your environment to help bring you back to the here and now (e.g., the sound of your loved one's voice).

Choosing Values

- Beginning ACT-MI (or returning to and reading this workbook) was itself an important choice. What was important enough to you that you chose to return?
- What feels like an important direction for you now?
- Are there any areas of life that matter to you, but you avoid? See if you can remind yourself of why they are important (making room for the pain that might emerge even just in thinking about them).

Accepting and Holding Experiences

- How are you holding your pain in this moment? Is there a way you could be more gentle or more accepting with it?
- Is there a rope that you're holding onto now that you can choose to drop?

Observer Self

- Whatever you might be feeling, thinking or sensing in this moment, pause and notice that there is a you that is having these thoughts and experiences. Recognize this part of you as the observer-self that always has been, always is and always will be the holder of your experiences.
- If your mind starts to argue with you, ask yourself, "Who is having this thought right now?"

Living Values

- Take a step, no matter how seemingly small, towards something that matters to you.
- Imagine some different possibilities of meaningful acts that you could do. What is one that seems available to you in this moment?
- Imagine a new way to live your values. Is there something you could try or someone that you could reach out to? Is there an area of your life that you haven't spent as much time in? Make some plans for how to expand and explore into this new frontier.

Session 13 Practice Exercises

Living Values

You're invited to continue taking steps in the service of living your values. What steps you take and when you take them is up to you but for change to happen, you will need to act.

Some opportunities to live our values are unplanned but others can be identified and planned out ahead of time. Planning out steps to live our values helps us practice ACT-MI skills and interrupt our patterns of avoidance. You're encouraged to plan out and take at least one step towards living your chosen values each day between ACT-MI sessions.

Using the worksheet on the following page can help you to plan and reflect back on the steps you took this week. The worksheet will ask you these questions that you can answer in boxes in the table:

- What step did you take to live your chosen values?
- Was your step planned or unplanned?
- What showed up inside you when you took the step?
- How did you choose to respond?
- What did you learn from the experience?

At the next group you'll be given an opportunity to share what steps you chose to take and your experience of living your values.

Use this table to plan and reflect back on taking steps to live a more meaningful and purposeful life before your next ACT-MI session.

	What steps did you take to live your values?	Was it planned or unplanned?	What showed up inside? (Thoughts, emotions, sensations, urges)	What did you learn?	What will you try next?
DAY 1					
DAY 2					
DAY 3					
DAY 4					
DAY 5					
DAY 6					

SESSION 14: CONTINUING FORWARD

Your journey with moral pain did not begin when your started ACT-MI. Your journey with moral pain also won't end when your involvement in this episode ACT-MI is over. The goal of your work in ACT-MI has been to help you learn new ways of relating to your moral pain so that you aren't distracted or pulled away from living a meaningful life, both in the here and now and as you go forward.

At the same time, it is common during times of change to feel disoriented or uneasy. We may wonder what the next chapters of our lives hold for us and how we will respond. These feelings are natural and normal. They signal that we care about ourselves, that we care about others and that we care about what's to come. The feelings that come with change also provide an opportunity to use the skills you learned in ACT-MI. Although as humans we can't know the future, when we feel uncertain or confused, we can always make the choice to return to the skills that have learned to be present and gently hold our current experience, knowing that it does not define us. Then, from a place of awareness, we can make choices about how to live our values in the here and now so that we are pointed toward the possible futures that reflect what matters most to us. Returning to these skills, as often and as many times as needed, allows us to continue moving forward as we strive to live lives of meaning and purpose for ourselves and those we care for.

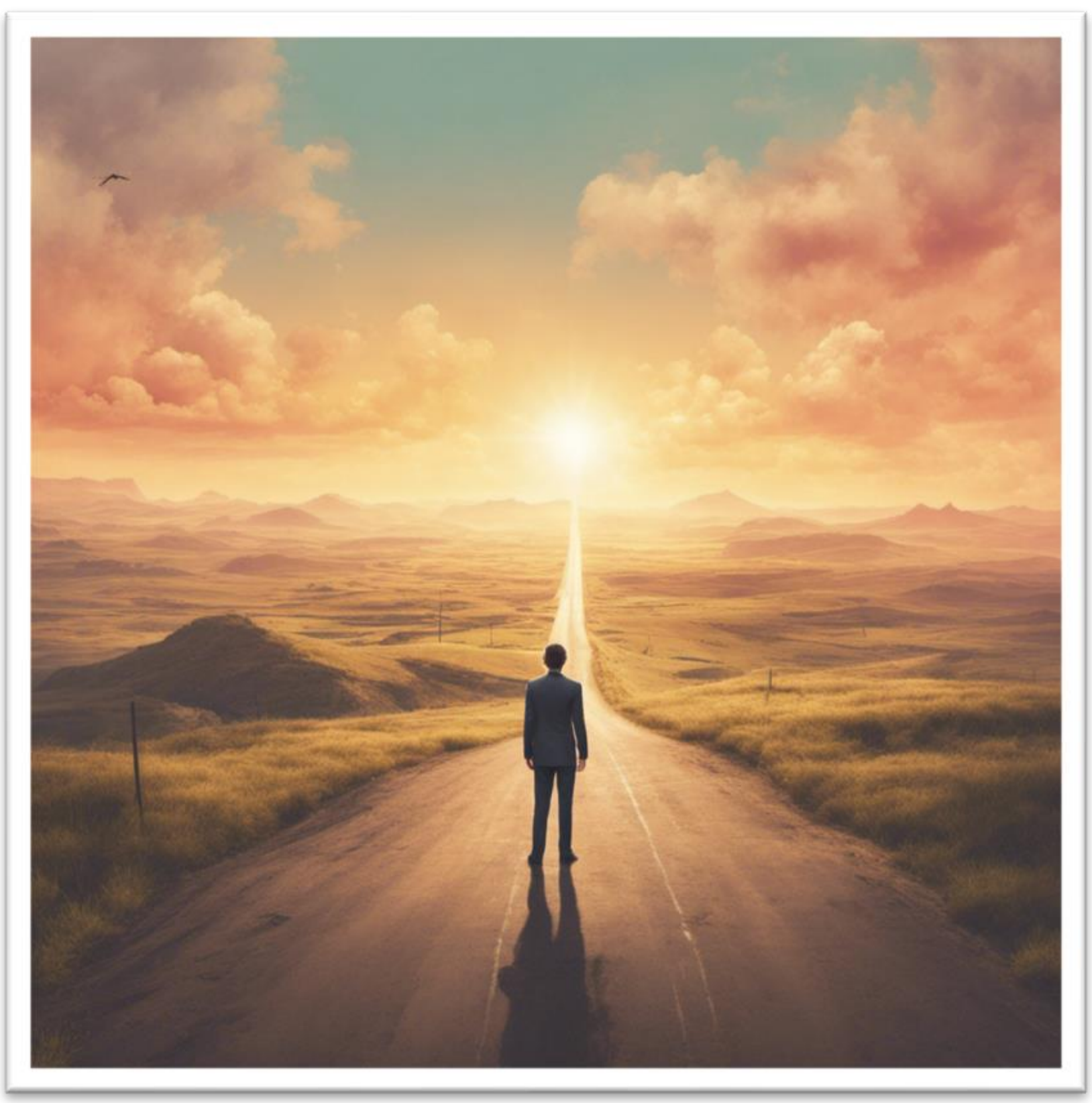

Session 14 Exercises

As you survey the many different moments across your life, you're invited to choose and reflect on some moments when you felt deeply connected to something important – moments you felt deeply grateful, or proud, or caring or inspired.

CONNECTING TO PLEASANT MORAL EMOTIONS FROM MY PAST

Think of a time before this group when you felt deeply connected to something important or something bigger than yourself. What was the event and why it was important to you?

CONNECTING TO PLEASANT MORAL EMOTIONS FROM ACT-MI

Next, think of a moment from your time in ACT-MI that was meaningful for you. What was the moment and what about it was meaningful to you?

CREATING NEW OPPORTUNITIES GOING FORWARD

Look over what mattered to you about the moments you chose above. How do you want to try and bring those same qualities into other parts of your life? What might you try to help this happen?

SESSION 15: CONTINUING TO LIVE OUR VALUES

There will always be opportunities to use chain analysis to practice the core skills of ACT-MI. This can include learning how to break patterns that lead us away from our values, but it can also include better understand what has helped us successfully live our values in the past. By understanding both patterns of behavior we become better able to live meaningfully in different areas of our lives.

As reminder a chain analysis involves…

1) Picking a behavior that we want to understand better (we call this the "target behavior").
2) Asking ourselves, "What triggered the target behavior this time?"
3) Exploring the steps between the trigger and the target behavior. Some examples of steps might include the things that were happening in the situation, the choices we made in response, and the thoughts and emotions we were experiencing around the time of the target behavior. Each step becomes a link in the chain that connects the triggering event to the target behavior.
4) Noticing if after the target behavior there was a change in our experience or how others responded
5) Reflecting on whether our target behavior had any long-term impacts on living our values. For example, did it help us live our values more fully or did it turn us away from them?
6) Look back over the links in the chain and explore places where the skills of ACT-MI might have been particularly important.

ACT-MI SKILL	QUESTION
**Being Present**	Slowing down and noticing what is happening in the present moment. Staying grounded in the here and now and all that the present contains.
**Accepting (Dropping the rope)**	Opening up to and allowing emotions, thoughts, or other internal experiences to be in this moment, just as they are. We might not enjoy or condone the events of experiences. Rather, we acknowledge and stop fighting against the reality of the events and the experiences connected to them.
**Seeing thoughts as thoughts**	Disentangling ourselves from thoughts and seeing them as just some of the many other experiences we may be having in each moment. When we see our thoughts as thoughts, we neither believe or disbelieve them but instead simply notice that we can be aware of what our minds may be telling us.
**Observer Self (Holding pain)**	Shift perspective to notice emotions and thoughts as things that we experience, but don't define us. Looking from the observer self allows us to connect to a part of ourselves that holds all our experiences, including moral pain.
**Connecting to what matters**	Connecting to what matters most to us in a particular situation and noticing what value(s) our pain might be alerting us to. This might include awareness of multiple values that might all be showing up in a particular moment.
**Living Values**	Taking deliberate action to live in a meaningful way. This can include taking concrete steps through our physical actions or practicing different ways of relating to our internal experience.

As before, you're invited to practice doing chain analyses on your own by following the instructions on the blank Chain Analysis Worksheet included in this section of the workbook. You can pick any behavior you want to better understand and do a chain analysis on it.

Breaking Patterns of Moral Injury Worksheet

Step A: Write down in **Circle 1** something you did in response to feeling shame, guilt, anger, disgust, or contempt that would be helpful to understand.
Step B: What triggered the target behavior **this time**? Write this in the circle labeled **2: What triggered the target behavior this time?**
Step C: How did you get from the **trigger in Circle 2** to the **target behavior in Circle 1**? Fill circles **3-9** with the thoughts, emotions, sensations, urges, behaviors, or actions that happened in between the trigger **(Circle 2)** and the behavior you're trying to understand better **(Circle 1)**.
Step D: What happened to your *internal experiences* immediately after the **target behavior**? Write this in **Box 10**. (Rate the intensity from 0-100)
What happened to your *external experiences* immediately after the **target behavior**? Write this in **Box 11**. (Rate the intensity from 0-100)
Step E: What are the long-term impacts of the **target behavior**? Write these in **Box 12.**

2: What **triggered** the target behavior *this time*?

10: What happened to **my** thoughts, emotions, sensations, urges, and memories right after? *(internal experiences)*

1: Target Behavior

11: How did **others** respond, or my surroundings change, right after? *(external experiences)*

3.

What happened next?

12: What are the **long-term impacts** of the target behavior in Circle 1?
(e.g., Relationships, spirituality, Impacts on mental and physical health)

9. (Just before the behavior)

4.

5.

6.

7.

8.

APPENDIX

Chain Analysis tips

- Pick a specific and recent action of your own as the target behavior. For example, choose "spent five hours playing video games in the basement" instead of "isolating". Do not choose someone else's actions as the target behavior.
- Choose a triggering event that is within hours or minutes of when the target behavior happened, instead of being in the more distant past (not days or years).
- If you have trouble identifying links in the chain, remember the triggering event and ask yourself "What happened next?" See if you can play back the situation in slow motion and catch additional parts of the event or your experience you may have not noticed before.
- It's OK if you get stuck or confused. Chain analysis is itself a skill that takes practice. You might want to consider reaching out your ACT-MI facilitators so they can help guide you.

Additional ACT resources

ACT and Related Self-Help Books (Print And Audio)

- https://portlandpsychotherapy.com
    - Go to: Resources → ACT exercises and audio files
- Get Out of Your Mind and Into Your Life by Steven Hayes, Ph.D.
- The Mindful Way Through Anxiety by Susan Orsillo, Ph.D. and Lizabeth Roemer, Ph.D.
- The Wisdom to Know the Difference: An ACT Workbook for Overcoming Substance Abuse by Kelly Wilson, Ph.D.
- ACT on Life not on Anger by Georg Eifert, Ph.D., Matthew McKay, Ph.D., and John Forsyth, Ph.D.
- Finding Life Beyond Trauma: Using ACT to Heal from Posttraumatic Stress Disorder and Related Problems by Victoria Follette, Ph.D. and Jacqueline Pistorello, Ph.D.
- Out of the Fire: Healing black trauma caused by systemic racism using Acceptance and Commitment Therapy by Jennifer Shepard Payne, Ph.D.

ACT and Related Mobile Phone Applications

- ACT Coach (VA App)- Designed for Veterans who are in Acceptance and Commitment Therapy with a mental health professional. Features six mindfulness exercises, tools to help identify personal values and take concrete actions to live by them, and logs to keep track of useful coping strategies. Available for iPhone and android. Cost: free https://itunes.apple.com/us/app/act-coach/id804247934?mt=8

- Mindfulness Coach (VA App)- Created to teach the concept of mindfully focusing attention on the present moment nonjudgmentally and serve as a way to practice this skill. Features nine guided exercises to practice the core concepts of mindfulness, education about mindfulness, and logs for keeping track of mindfulness practice. Available for iPhone. Cost: free https://itunes.apple.com/us/app/mindfulness-coach/id804284729?mt=8
