# Supplementary material for: Acceptance and commitment therapy for moral injury: conceptualizing and intervening on moral injury through the lens of contextual behavioral science
Source: Front Psychiatry. 2026 Sep 9;17:1923699. doi: 10.3389/fpsyt.2026.1923699 (PMC13597758; doi:10.3389/fpsyt.2026.1923699)
Supplement: Supplementary file 2 [file Image1.pdf]

# Acceptance and Commitment Therapy for Moral Injury (ACT- MI)

## Clinician Outlines

Borges*, L. M., Farnsworth*, J. K., Drescher, K. D., Barnes, S. M. & Walser, R. D. (2026). Acceptance and Commitment Therapy for Moral Injury (ACT-MI) Clinician Outlines.
*Both authors contributed equally to this manual

## SESSION 1

## CASE CONCEPTUALIZATION AND FUNCTIONAL ANALYSIS

### Purpose of Session

The primary purpose of this session is to begin to understand the behaviors that are maintaining the client's moral injury. A second purpose is to help the client contact the personal costs of these morally injurious behaviors considering how it is interfering with living their values. Because the majority of this intervention occurs in a group context, these individually focused sessions offer the opportunity to better understand the processes maintaining moral injury at the level of the individual Veteran. Once the specific behaviors maintaining a morally injurious behavior are identified and the function of these behaviors are revealed, the patterns of behavior that maintain moral injury can be addressed. New behaviors can be introduced that facilitate engagement with healthier alternative patterns of behavior that instantiate meaning and purpose. Furthermore, the content of these individual case conceptualization sessions can be used to help focus and tailor discussions on the factors most salient to each group member's moral injuries in subsequent group session.

### Materials Needed

- ACT-MI Chain Analysis Worksheets

### Pre-Session Clinician Preparation

- 1-minute mindful check-in with self (noticing own contact with the present vs. conceptualized pasts and futures, potentially including those about group members)

### SESSION CONTENT

### Welcome and Introduction

The clinician welcomes the client and introduces themselves.

### Introduce Case Conceptualization/Functional Analysis

*This meeting's purpose is to better understand how moral injury shows up in your life. I'll be asking you some questions to guide our discussion, but I want you to feel free to share anything that feels relevant to better understanding your experience. Then, in our group sessions, we will focus on what we have learned, inviting you to consider techniques and strategies useful in helping to interrupt any problematic patterns we discover today and that are keeping moral injury alive. As part of these group discussions, we as Clinicians may reference some of the behaviors you and I discuss today so if there is anything you don't want us to share in group, please let us know.*

| Example Clinician Language | Notes for Clinician |
|---|---|
| **Clinician Note:** Although chain analysis can be a clarifying and valuable tool for helping clients understand their behaviors, it is important to also track the client's emotional experience of the exercise. For example, some clients may find chain analysis to be a validating experience in that it helps them see that their suffering stems from understandable, if unhelpful, motivations to escape pain. Yet other clients may experience the chain analysis as emotionally evocative as they contact emotions and memories they otherwise attempting to avoid. | |

| | |
|---|---|
| **Identify Morally Injurious Event** | |
| *What morally injurious event would you say you get most stuck in or struggle with the most?*<br><br>*How has this event come up for you recently? What kinds of things make you remember it?* | Identify the morally injurious event and an example or two of situations that tend to trigger it. |
| **Identify Target Behavior** | |
| *When you think about why you signed up for this group, what would you most like to see change? What behaviors interfere with living well and doing what matters to you? W*hat do you struggle with the most?<br><br>[If unclear how behavior is connected to moral injury] *What do you do in response to guilt, shame, anger, contempt, and/or disgust when it comes up for you?*<br><br>*Why does changing these behavior matter to you? What do you hope for when you think about your future and how you want to live your life?* | Identify target behavior for chain. This<br><br>Connect client to their felt sense of why changing this behavior matters to them |
| **Identify Triggering Event and Vulnerability Factors** | |
| *What kicked this chain of events off for you…what triggered xx behavior?*<br><br>*Did anything make it more likely you would respond to [prompting event] in the way you did? (e.g., prior alcohol use, poor night's sleep)* | Identify triggering event for this specific instance<br><br>Explore potential vulnerability factors for target behavior |
| **Fill in steps between target behavior and prompting event** | |
| *What happened after you did [X]?*<br><br>*How did you get from here* [prompting event] *to here* [target behavior associated with moral injury]*?* | If needed, invite the client to slow down in their recall so that significant elements of the chain aren't missed or overlooked. |
| **Clinician Note:** The chain/functional analysis is meant to tell a story of the reinforcer(s) maintaining the Veteran's moral injury. If up the exercise starts to feel confusing or scattered, bring yourself back to the purpose of the exercise—to learn which behaviors are providing them relief or reward, and in so doing keeping their moral injury alive. The other primary purpose is to understand what the costs of these behaviors have been for the client. | |
| **Introduce Short-Term Consequences** (Why we keep doing what we do) | |
| *People keep doing these behaviors because of the effect it has on us or our surroundings. If a behavior reduces something we don't like to feel, we keep doing it. If we are anxious and feel relief by isolating, for instance. If it increases our contact with something that feels good, we keep doing it. If you feel anger* | Morally injurious behaviors, by definition, lead clients to depart from values or cause themselves or others added suffering. |

| | |
|---|---|
| *and you drink, and you feel more lose, for instance. It makes sense. Feeling better drives a lot of behavior. It's important that we know how this behavior impacts you and your environment—including other people around you—in the long run, so that we can identify strategies and tools you can use to give you more choice about what you do in response to moral pain.* | Providing education about how short-term consequences give immediate reinforcement can help give client's clarity as to why they have engaged in behaviors that otherwise appear costly and destructive to themselves and others. |
| **Identify Internal Short-Term Consequences** | |
| *What do you notice immediately happened to your experience during or after [target behavior]?*<br><br>*What happened to your emotions…Your thoughts…Your sensations…Your urges…your memories during and/or immediately after engaging in the target behavior?*<br><br>*Did anything shift about the intensity of these experiences?*<br><br>*What did you notice about how this behavior affected you in the moment?*<br><br>*Did this behavior take away or reduce anything you didn't want to feel? (Did it give relief from something painful? Turn down the volume on the pain?)*<br><br>*Did it increase your ability to feel something you would rather feel instead? (Did it lead to something enjoyable?)* | Invite the client to slow down and focus on the experience during or immediately after their target behavior.<br><br>Clients may feel embarrassed to acknowledge that their unwanted target behavior gave them relief or comfort. If needed, reiterate that before a behavior can be changed, it can be helpful to know how it is making our lives easier or more comfortable in the moment, even if it is costly in the long-term.<br><br>Examples: avoiding guilt and shame, reduced anger, distracted from painful memory<br><br>Examples: sense power/control over situations, atonement, connection to people who have passed away |
| **Clinician note:** During this session the clinician can begin modeling ACT-consistent language and concepts (e.g., so you're noticing your mind saying). Take opportunities to connect the links in the chain to values. | |
| **Identify Long-term Consequences** | |
| *How does this behavior affect your relationships, spirituality, mental health, physical health, work?*<br><br>*What are the long-term impacts of the target behavior on you?* | Probe how the target behavior affects a range of functional domains (social relationships, employment, physical health, finances, religion/spirituality) |
| **End Chain Analysis** | |
| *Thanks for working with me to better understand how these patterns of moral injury show up in your life. I want to check in with you and see what you've take away from this session.* | Clinician listens, validates, and responds supportively |

| | |
|---|---|
| *What stands out to you?* | |

**Closing of Session**

- Check for understanding about the assignment and invite any further questions from the group members and discuss any issues that are brought up.
- Thank group member for their attendance and state your anticipation to see them the following week.

*In this session we've begun identifying some of the patterns of behavior that are keeping moral injury alive for you. We'll have the opportunity to individually check-in again in the future to update and explore this more. In the meantime, I want you to take what we've started here and notice when these patterns are happening in your day-to-day life.*

*When you come to group next week, we'll be helping you learn some techniques and tools for breaking these patterns so you can be free to live your life with more meaning and purpose.*

**Post-Session Clinician Review**

- Check-in internally with experience of session and reflect on personal or clients shifts that were noticed.
- Identify potential patterns of behavior observed to be aware of in future sessions.
- Compassionately identify any self-care needs that became evident during the session and, if willing, make plan to address them as soon as workably possible.

## Breaking Patterns of Moral Injury Chain Analysis Worksheet

### Breaking Patterns of Moral Injury

**Step A:** Write down in **Circle 1** something you did in response to feeling shame, guilt, anger, disgust, or contempt that would be helpful to understand.
**Step B:** What triggered the target behavior **this time**? Write this in the box labeled **2: What happened that triggered…**
**Step C:** How did you get from the **trigger in circle #2** to the **target behavior**? Fill circles **3-9** with the thoughts, emotions, sensations, urges, behaviors, or actions that happened in between the trigger **(Circle 2)** and the behavior you're trying to understand better **(Circle 1)**.
**Step D:** What happened to your *internal experiences* immediately after the **target behavior**? Write this in **Box 10**. (Rate the intensity from 0-100)
What happened to your *external experiences* immediately after the **target behavior**? Write this in **Box 11**. (Rate the intensity from 0-100)
**Step E:** What are the long-term impacts of the **target behavior**? Write these in **Box 12.**

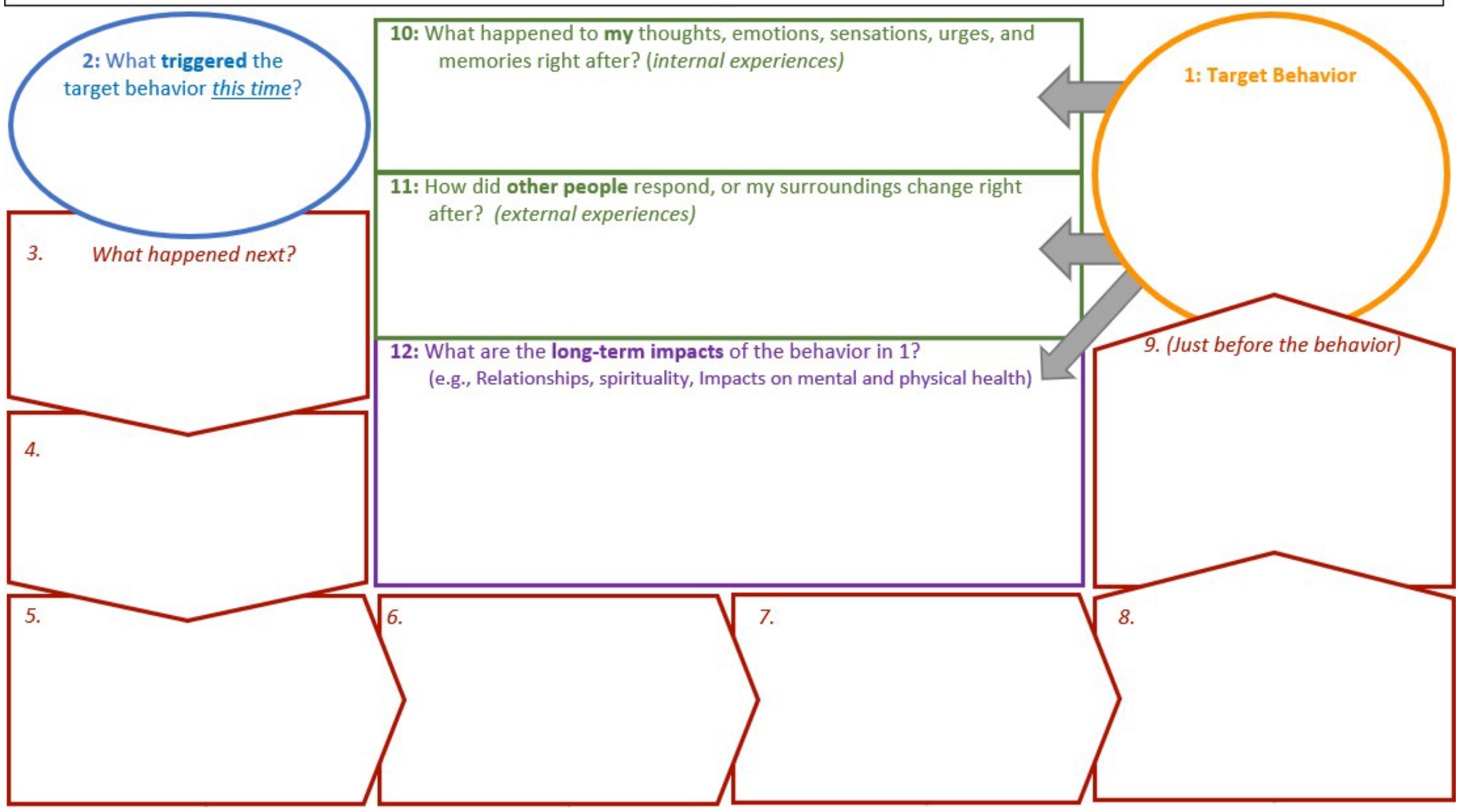

**Common Chain Analysis Challenges and Possible Clinician Responses**

| Target Behavior Challenges | Possible Clinician Response |
|---|---|
| Client gives a broad, generalized target behavior (e.g., drinking) | Elicit further specific details (e.g., Tell me a time when you were drinking. Where were you? How much?) |
| Client identifies another person's behavior | Validate the event and invite them to share what they did in the situation that might be keeping them stuck. |
| Client gives a covert behavior (mental actions) | Clinician may need to provide more labeling (i.e., labeling the behavior 'rumination') |
| Client gives an example of NOT doing a valued behavior | Help the client identify what they were doing instead of the valued behavior |
| **Prompting Event Challenges** | **Possible Clinician Response** |
| Client has trouble identifying what the prompting event is because it happened so quickly | Work backwards from target behavior to get the full episode of the behavior ("*What happened right before*…") |
| Client has trouble identifying what the prompting event is because it feels so routine | Explore if there was something distinct about the timeframe/circumstances of this particular episode (Tell me what was unusual about this particular day)<br><br>Explore events/situations that may serve as vulnerability factors (e.g., anniversaries, alcohol use) |
| **Link Challenges** | **Possible Clinician Response** |
| Clinician or client feels unclear about the timeframe of the chain | Identify the time span between the prompting event and target behavior happened (e.g., two hours) |
| Chain feels unclear or doesn't tell the story of the behavior | Be direct and curious with the client about this and invite the client to collaborate with you in helping to understand potential missing links |
| Client experiences shame and self-critical thoughts in response to chain analysis | Use frequent validation techniques such as, validating their experience, normalize the experience as a product of their prior experiences. Eventually validate by recognizing short-term consequences that reinforce the behavior. |
| Clinician experiences discomfort in reaction to client's moral pain | Use ACT-consistent skills to lean into and validate both the client's and clinicians own experience |
| **Short-term Consequence Challenges** | **Possible Clinician Response** |
| Client is focused on long-term consequences of the target behavior and so can't validate that their own experience makes sense. | Reorient the client to the immediate consequences of the target behavior (e.g., during the target behavior or seconds or minutes after). "*What happens in that moment as you are* [insert target behavior]?" |
| Client doesn't report their target behavior reduces painful emotions and so can't be reinforcing in the short-term. | Explore whether the target behavior is ***positively*** reinforcing, such as increasing a sense of control or by creating a more preferred emotion (e.g., feeling guilt rather than shame). |
| **Long-term Consequence Challenges** | **Possible Clinician Response** |
| Client doesn't report their target behavior has long-term negative consequences for them. | With curiosity and openness, inquire what the costs of this behavior have been in the past. If the client cannot identify any costs, explore further what has brought them into treatment and see if any long-term consequences of |

| | |
|---|---|
| | morally injurious behaviors can be identified. If none can be identified, consider checking with the client's referral source and clarifying purpose of ACT-MI as needed. |

# SESSION 2

# THE COSTS OF CONTROL

## CLINICIAN ORIENTATION AND PREPARATION

### Purpose of Session
This session's purpose is to introduce ACT-MI's framework in a way that simultaneously validates and promotes cohesion among group members. This session will begin to create a social community among group members that can be used to help practice connecting to others even in the presence of moral pain by contacting the costs of avoidance.

### Materials Needed
- ACT-MI Workbook

### Pre-Session Clinician Preparation
As a Clinician, pause before the group to mindfully check in with yourself and how you are orienting to this first session. Notice if you feel rushed or overwhelmed, tired, or irritated. Allow yourself a moment to pause and notice your experiences in the present moment, making space for them, acknowledging that these experiences are an important part of being human. In doing so, you may also notice how any experiences of pain and discomfort relates to the pain and discomfort of the group members you are about to interact with, and the sense of shared humanity this creates. Consider creating a pattern of slowing down and dropping into the present moment for a few minutes prior to the start of each group. Consider how this practice might map onto the qualities you would like to bring to a group focused on moral injury as a therapist.

## SESSION CONTENT

### Bridge from Previous Session
*It's nice to see everyone today. Today for our first group session of ACT for Moral Injury, we're going to spend some time getting to know one another and creating a shared understanding of what we want this group to be like. We're then going to spend most of our time exploring what we mean by moral injury and understanding how it has shown up in your lives. We'd like to start by having everyone here introduce themselves. [Clinicians and group members introduce themselves]*

### Set Group Norms and Expectations
*In this group, we want to create a community that has enough trust and safety so that it's possible to be honest and try new things both inside and outside of session. What has been helpful in therapy groups or group scenarios in the past in building trust and safety?*

Clinicians invite group members to share expectations that may assist group in functioning well. These may include confidentiality, not interrupting others, consistent attendance, gauging appropriate level of details. If not mentioned, Clinicians should also address at this point the following topics:
- Don't come to group while intoxicated or having recently used
- The limits of confidentiality/mandated reporting
- How to handle crisis situations (Vet's crisis line, 911, ED)

For Telehealth groups, additional strategies may need to be set, such as:

- To preserve confidentiality, participants should call in from a private room where they are alone
- Participants cannot be driving or riding in a car during group (they may call in from a parked vehicle if they are alone)
- Please be fully dressed and in an upright position
- Do not eat, smoke, or use substances during session
- Cell phones should be turned off or on airplane mode
- Do not text, use email, use the internet, or engage in any other activities
- No recording of virtual sessions is allowed
- Please be sure to have devices plugged in or fully charged prior to the group
- Place device in a position where the camera can consistently see your face

**Connect Group Norms to Purpose of ACT-MI**
*The reason for these group norms is so we can create a community where we can try out different skills together. They'll also help us stay focused on the purpose of this group, which is helping each group member connect to greater meaning and purpose in their lives. We know from experience that talking about moral injury can be uncomfortable, and that might mean you start to repeat some of the patterns that have kept you stuck in the past. Our job as Clinicians is to help you catch these patterns as they're happening in the moment and give you new ways to respond to them.*

**Bridge to Previous Content**
*Before this session, we met individually with everyone here to learn a little bit about how their moral injury is showing up in their lives. We did that so we could understand you all better but also because it's these patterns of moral injury that we are going to be focusing on and working with throughout the course of this group. We'd like to spend some time exploring how the ways you've coped with your moral pain so far have worked for you.*

**Introduce New Content**
Briefly/Introduce mindfulness/Contacting the present moment using an exercise that draws on the group context—experiencing these things together in the presence of each other)

*We'd like to start by introducing and practicing the most basic and foundational skill that we will use throughout this group. That skill is learning to be in the present moment. Being present means we slow down and simply notice what's happening here and now. It can be difficult to keep our attention on the present moment. Our mind's like to wander or get caught up in thoughts and feelings. To start practicing we're going to guide you to focus on some particular parts of your experience that are relevant to why we're here.*

*We'd invite you to close your eyes… [Provide basic instructions for starting mindfulness] We invite you to recall the struggles that you discussed in your individual session with us. Observe the thoughts and emotions that you have struggled most with… Notice the things you've tried in the past to make these go away or resolve them… Reflect back on the toll it is taking on you and what you care about.*

*Also notice that everyone here has their own version of that struggle… and like you they've come here to this group seeking a better way… "*

**Bridge to New Content**

*We'd now like to invite the group to share together how their morally injurious events have impacted them. [Get out* Moral Pain and Moral Injury figure]

**Experiential Exercise: Creative Hopelessness Exercise using Moral Pain and Moral Injury figure**

- Conduct experiential and process elements
- Respond to challenges in comprehension or application of exercise
- Generalize skills to outside of session

<table>
<tr><th>Example Clinician Language</th><th>Notes for Clinician</th></tr>
<tr><td colspan="2">Identify moral pain stemming from MIEs</td></tr>
<tr><td>After your morals were violated, what was different about your thoughts, emotions, and behaviors? How did these events change you? When did you notice these changes?<br><br>How did these events change how you interact with others? How did it change how you interact with yourself? [List examples in center of the board and circle/label these "moral pain"]</td><td>Clinicians should slow down and allow group members space to connect to their moral pain and the costs of decades spent avoiding or trying to control that pain. Continue to move slowly and support group in contacting the futility of avoiding/controlling that pain- in and out of the exercise.</td></tr>
<tr><td colspan="2">Identify group member's avoidance/control strategies</td></tr>
<tr><td>In our sessions last week, we explored ways that each of you have learned to avoid, control, and escape. Oftentimes when we feel pain, we look for an escape to stop experiencing it…to numb it. We learn early on to pull back from pain. To get rid of it, to throw it out…to do everything we can to try to control it.<br><br>What have you done in response to your moral pain? What have you tried to do with it?"<br><br>In ACT-MI, it's this additional pain that we define as "moral injury". It's the new suffering created by trying to control or avoid our moral pain.</td><td>List control strategies outside of the moral pain circle.<br><br>Watch for avoidance/control behaviors during the exercise (ex., humor, distraction). Consider gently noting these behaviors.<br><br>When done, label this larger circle "Moral Injury" in the figure</td></tr>
<tr><td colspan="2">Clinician Note:<br>Examples of control strategies can include (but aren't limited to) isolation, suicidal ideation and/or behavior, self-injury, restricting pleasure, contacting preferred emotions, numbing, controlling relationships or interactions, aggression, substance use, sex/pornography, food, risk-taking, investing into stories about us/others, psychotherapy, medications, trying to convince self that MIE was justified, self-punishment, compensating by doing good deeds, and making amends.</td></tr>
<tr><td>How often do you use these strategies? How many years have you been living this way? Let's add up how many years you have collectively tried these strategies in the group? [After totaling] So it's been [##]collective years trying to control. Let's pause for a moment and look at this image. Here are all the ways everyone in this room has tried to avoid, control, get</td><td>Help group members identify the cost of strategies in terms of lost time and additional suffering to themselves and others.</td></tr>
</table>

| | |
|---|---|
| *rid of moral pain. See beyond the words themselves and try to connect to the struggle and pain they represent.* | Allow group members space and silence to experientially contact this cost. |
| *What do you feel as you look at this? Can you locate those feelings inside of you?  What's the weight of that feeling? As you sit with this pain, what do you notice? What strikes you in your gut as you look at this?* | Allow them time to identify the experience in their bodies.<br>Listen for attempts to solve or escape through insight, analysis, declarations or problem-solving. |
| *What do you notice about being here with other Veterans who are also sharing about their pain? What is similar about pain across group members?* | Look for recognition of commonality. If any express feeling different, ask others if they have also felt separate or disconnected in the past. |
| **Processing** | |
| *Looking at the past [##] years of avoidance and control…. [list strategies]…what has this pattern of avoiding and controlling moral pain cost you? From what have these behaviors taken you away? How have these behaviors blocked… narrowed… controlled your life?* | Probe areas that might evoke moral pain…Family? Spirituality? Self-care? Other relationships? |
| *Has this avoidance and control gotten you closer to the life you hope to live? Has it been working?*<br>*[If needed] How have these behaviors impacted your moral pain in the short and long-term?* | Members might note that avoiding:<br>- isn't resolving the underlying pain<br>- expanded to dominate their life<br>- feels like all they know and are |
| *As we reflect on the time you've spent avoiding and controlling this pain, what do you feel? See if you can notice what your mind does….what thoughts are you experiencing? What urges do you have in response to this?* | Notice and name moral pain and the urges to avoid/control it |
| To close the exercise, Clinicians emphasize to group members:<br>- Avoidance behaviors, especially costly ones, take effort, suggesting that a part of them still cares<br>- All this work isn't working in the long term (i.e., moral pain isn't gone) and past efforts to control it have instead often created more pain and suffering<br>- That this process is one that is happening for each member of the group (so it's not something that is unique to just one person). | |

**Bridge**

*The purpose of this group is to understand the behaviors that keep you stuck in a cycle of moral injury and to put you in touch with choice…. to learn to hold your moral pain differently so that you have the option to do something other than trying to get rid of/suppress/control it, and to explore behaviors that bring you closer to what matters to you and expand your life. You can continue to avoid and control your moral pain, but you can also lean into it and see what it has to say about what matters most to you. We'll do this together through our work in ACT-MI.*

**Acceptance and Commitment Therapy for MI Core Skills**
*This group will be focused on practicing new ways of relating to your moral pain—not to solve the pain, but to build a meaningful life even in the presence of painful thoughts and emotions. We will also practice identifying what matters to you in your life and practice skills strengthening your ability to make choices based on what is meaningful to you rather than allowing your moral pain to drive your behavior. Through this process we will support one another in beginning to build lives based on what you find most meaningful. Some skills for breaking free from moral injury include:*

| ACT-MI SKILL | DESCRIPTION |
|---|---|
| Being Present | Slowing down and noticing what is happening in the here and now |
| Accepting | Opening up to and allowing emotions, thoughts, or other internal experiences to be in this moment, just as they are. |
| Seeing Thoughts as Thoughts | Disentangling ourselves from thoughts and seeing them as just some of the many other experiences we may be having in a given moment. |
| Observer Self | Shifting our perspective to notice emotions and thoughts as things that we experience, but don't define us. |
| Connecting to Values | Connect to what matters most to us in a particular situation. |
| Living Values | Taking deliberate action to live our values. |

**Explore Client Concerns, Questions and Motivations**
*In contacting some of the costs of avoiding and controlling moral pain and learning more about what this our focus will be, you might notice your mind having reactions. We would be interested in knowing what they are. What questions might you be experiencing? What questions come to your mind? If you notice any barriers, what are they? What barrier might there be to fully connecting with the group?*

Common reactions might be Fear of discomfort, fear of reprisal/punishment, return to former coping behaviors, fear of judgment. If concerns are expressed about clinical documentation, Clinicians can point to the sample session note in the back of the Patient Workbook if concern over documentation is expressed.

**Explore Client Goals and Motivations for Treatment**
Given that participating in the group poses risk of significant discomfort, invite group members to share what makes it worth taking such a risk. Clinicians may also share their motivations for leading a group on such a difficult topic and what they find important about this topic.

*If this were a successful therapy, how would things be different for you? What are you hoping to gain from it?*

*What similarities do you notice as far as what brought you here?*
Clinicians start to link these similarities to potential shared valued domains across group members.

Emphasize goal of building a supportive social community in this group so that you can practice relating differently to your pain in the presence of other people with similar histories and support each other in exploring new opportunities in life.

**Bridge to Skills Practice**
*We will be practicing skills each week to build a life guided by your values even with moral pain. The practice exercises are how you can take what you learn in session and apply it to your lives. In this first week we will be starting the work of exploring your values…what you care most about and how you want to live in these areas of your life.*

*In hearing about what you want from treatment…what matters to each of you individually and as a group…building a social community together…there are some clues about what you might focus on as your values...what drives you to keep building a life even with moral pain. This practice exercise will be focused on identifying what matters to you in different areas of your life. We will build on this throughout group.*

**Skills Practice**
[Review instructions provided in the ACT-MI patient workbook about how to complete the values sort exercise.]

**Closing of session**
- Check for understanding about the assignment, invite any further questions from the group members, and discuss any issues that are brought up.
- Thank group members for their attendance and state your anticipation to see them the following week.

**Post-Session Clinician Review**
- Check-in internally with experience of session and reflect on personal or clients shifts that were noticed.
- Identify potential patterns of behavior observed to be aware of in future sessions.
- Compassionately identify any self-care needs that became evident during the session and, if willing, make plan to address them as soon as workably possible.
- Depending on your level of knowledge about the members in the group, you may also wish to bring each one to mind in turn. Briefly bring their face to your mind and reflect on what you already know about their history, their goals for treatment, and their concerns about coming to group. With their faces held in awareness, contact the part of you that hopes for their recovery and can envision them actively pursuing their personal values in their day-to-day lives.
    - As Clinicians, frequently look for opportunities to use the group member's experience to articulate the points in the outline below, rather than teaching, instructing or listing these points.

- After the group members have left, take a moment to turn your attention inward and notice your own experience. What emotions, thoughts, sensations or urges are you experiencing? What, if anything, are you caught up inside? Who or what are these experiences directed towards? With awareness of your own internal experience, also reflect on the behaviors and statements made by the group members in today's session. Did there appear to be a sense of cohesion? Did there appear to be apprehension or ambivalence by group members toward participation in ACT-MI. While lightly holding both your experience of yourself and your experiences of the group members, make a few observations or notes about processes or patterns that might be clinically meaningful to pay special attention to in subsequent group sessions.

# SESSION 3

# DROPPING THE ROPE

## CLINICIAN ORIENTATION AND PREPARATION

### Purpose of Session

This session's purpose is to connect to motivation within the group member for interacting differently with moral pain. One source of this motivation comes through experientially contacting the costs of avoiding and controlling moral pain. Clinicians guide group members in examining whether excessive and misapplied control, especially toward internal experiences, is perpetuating suffering. Letting go of this type of control, in exchange for a life guided by values, is at the core of the ACT work done in this session. Clinicians offer values as a guide for Veterans to live more meaningful lives rather than remaining stuck in a costly battle against moral pain.

### Materials Needed

- ACT-MI Workbook

### Pre-Session Clinician Preparation

- 1-minute mindful check-in with self (noticing own contact with the present vs. conceptualized pasts and futures, potentially including those about group members)

## SESSION CONTENT

### Review and Process Practice Exercise from Previous Session

Revisit the creative hopelessness diagram from previous session. Note that today's session will transition into exploring how this pattern shows up for them personally.

*We'd like to revisit the place we ended last week. We listed the forms your moral pain takes (read a few examples)… and the strategies that you've all tried to control it (read a few examples)… this behavior has created these [# of] years of suffering. We'd ask you to close your eyes and as you consider those [# of] years of suffering collectively… We would ask you to notice what shows up inside of you. What emotions, thoughts, sensations, and urges arise? See if you can spend a few moments observing… watching… the rise and fall of your own experiences as you stop to sit with to all of this suffering.*

*What emotions do you notice?*

*What urges do you notice? What do you want to do right now? How do you typically respond to that urge when it shows up in your life? Is that something you've done before? How has that worked for you?*

### Introduce New Content

*As we just connected with, efforts to avoid or control our painful experiences often don't work in the long-run, and they create real costs for our ability to live our values. We want to dive in deeper to understanding how you respond to these moral emotions…and the costs of these patterns of responding on your life.*

*We're going to invite you to imagine some things in order to help you explore how your efforts to manage your moral pain thus far have impacted your ability to live your values. We'll be inviting you to create some images in your mind and then ask you questions about them. We may redirect you at times to help the conversation stay focused.*

**Experiential Exercise**

- Conduct experiential and process elements
- Respond to challenges in comprehension or application of exercise
- Generalize skills to outside of session

**Introduction**

*We invite you to join us in an exercise where we will ask you to practice observing your experiences surrounding moral pain and values. In this exercise, we want you to practice noticing the part of yourself that gets hooked by moral pain…how being stuck fighting guilt, trying to control shame and disgust or getting caught up in anger and contempt can prevent you from living your values and connecting to what matters most to you.*

*For the next little while in this session, we will ask you to go back and forth between imagining a scene and coming out of that scene and back to the room to share what you noticed. During the parts when you have your eyes closed, notice your experiences as they arise inside of you without commenting on them to the group. When you have your eyes open, we will ask you to share parts of your experiences with each other. We'll be guiding you through each step of the exercise. You might have urges to discontinue this exercise. We'd ask that you do your best to notice those urges and stay here in this practice with us in spite of any urges to avoid or control your pain…this is for a purpose…it's related to those values you identified as reasons to participate in this treatment.*

| Example Clinician Language | Notes for Clinician |
|---|---|
| **Clinician Note:** Conducting creative hopelessness exercises always needs to be done in a way that is sensitive to the well-being of the client. Many group members already struggle to believe that recovery is possible, and some may be at increased risk for suicide. Therefore, Clinicians must make it very clear that it is not the client or their values that are hopeless, but rather it is the strategy of avoidance and attempting to control one's experiences at all costs that can become unworkable or limiting to their growth.<br><br>People will be caught in efforts to control their pain throughout the group (we as Clinicians also get hooked by efforts to control our pain daily). Creative hopelessness is a process that should be ongoing over the course of ACT-MI both in a planned way (e.g., drop the rope exercise) but also as opportunities arise where group members might benefit from revisiting creative hopelessness experientially. | |
| **Experiential** | |
| *First, we'd invite you to close your eyes and begin imagining a scene together. Imagine a scene where you violated your morals in war…one that triggers moral pain. The scene should bring to mind the emotions you have…shame…the guilt…the anger…the disgust…the contempt you may feel. The thoughts... stories… and memories you have about yourself and others from when your morals were violated. We're going to ask you to imagine that moral pain as some kind of form or object or shape. We'd like you to imagine you have a way to pull the moral pain out so that you can see it and sense it* | |

*and picture it in this room…maybe that shape takes the form of a living thing…maybe your pain is a monster or maybe it's an object like a rock. Pick something that visually symbolizes the anger, shame, guilt, contempt, disgust that you struggle with the most.*

*What does that shape look like? What color is it? How big is it? Does it move or make sound? <PAUSE> When you have it, briefly raise your hand to let us know*

**Processing**

| | |
|---|---|
| *Now holding that shape in your mind…we'd ask you to open your eyes…*<br><br>*What did you see as you imagined your moral pain taking a form? [tailor the exercise going forward to include the clients' descriptions of their moral pain]*<br><br>*What urges do you experience in relation to seeing the pain in this form? What does it make you want to do?* | If a member has difficulty deciding on an image, Clinicians can guide them in choosing one for the purposes of the exercise<br><br>Invite group to simply be aware of their urges and stay in the present with the group. |

**Experiential**

*Now close your eyes again… We'd ask you to imagine an image of yourself facing that [image/shape/monster] representing your moral pain. This is the You that has struggled for years, maybe even decades, with that moral pain… the You that tries to control and avoid the shame…anger…guilt…all of it. It's the You that is in this room right now that struggles with this moral pain. Imagine yourself—maybe dressed as you are today…looking at the image you just called to mind.*

*Imagine you can see it clearly…it's close to you…across from you. You are staring at that pain.*

*And now, imagine between you and the image is a deep dark pit.*

*And in your hands, you're holding a rope that is stretched across the dark pit. On the other side of the pit is that image…your moral pain…either pulling the other side of the rope or attached to it in some way. You are grabbing one end of the rope and your moral pain is grabbing the other end, with the pit between you.*

*The rope is made up of all the strategies used to avoid or control the pain. Each thread is another strategy or time you tried to avoid/control.*

*Now imagine that you are in a tug of war with this monster or form. You are pulling hard…using all your force... to try and win the war…to prevent yourself from being pulled into the pit.*

*As you clench your hands around the rope in this battle notice how tight your grip is. Notice what the rope feels like. Maybe it's burning in your hand. Notice your body pulling and straining to win this war, your teeth gritted, using all your force to try to pull it into the pit…. to try to get rid of your moral pain.*

*Notice how long you have been in this tug of war.*

*And notice that if the monster or form wins, you lose, you fall in the pit.*

*So just notice how it has been your job to grip the rope tighter, pulling ever more forcefully ……feeling your nails digging into your palms as you battle that pain in the tug of war.*

*And you are still pulling …even today …and now notice, no matter how hard or long you pull…that monster or form…that pain isn't falling into the pit…the monster is still across from you…you keep pulling to try and win this war.*

*Now imagine that you could rise up out of this You that is in the war with the monster and begin to observe the war, watching a version of yourself fighting in the tug of war against the monster or form…against the moral pain. turning your attention and watching yourself in the struggle.*

*Look at the You who is fighting this tug of war… What do you see behind their eyes as they fight this battle? What are they feeling? What sensations are they experiencing? What are they thinking? What memories arise? What urges come up?*

*As you watch this version of you fighting your moral pain in the tug of war, notice how long they have been in this tug of war….notice that no matter how hard they pull…the pain isn't going anywhere… Also notice as you look at them in this battle that they are not free, they are consumed by winning – the threat of being pulled into the pit is too big – they must fight. The battle against the pain takes all their attention…consumes their life.*

*Now come back behind your eyes, be in the battle.*

*Notice what you aren't able to do while gripping this rope… trapped in this tug of war. Notice that you are unable to connect with family, you are unable to participate in your spirituality? Notice if you can take care of yourself physically…emotionally? While you are in this fight against your moral pain, notice how your work is affected…maybe you are unable to work…or you are overworking at the expense of other things that matter to you. Is it possible for you to live your values while in this tug of war…expending all your effort to fight your moral pain?*

| **Processing** | |
|---|---|
| *Now pause and open your eyes…*<br><br>*How long have you been in this tug of war?*<br><br>*What has this tug of war cost you in terms of what you care about?*<br><br>*How has your ability to live your values been impacted by fighting your moral pain?* | Draw out the negative impacts to domains such as family, other relationships, health, religious and spiritual involvement, legal involvement, finances, etc.<br><br>Consider lost time and lost opportunities as costs. |

**Experiential**

*Now close your eyes… You're back into the tug of war… Get behind the eyes of that you who is pulling against your moral pain again…your hands grasping around the rope…fists clenched around it…your nails pressing into your palms…pulling against the image…the monster…trying to pull it into the deep pit.*

<table>
<tr><td colspan="2">But notice now, that you have a choice…you can keep fighting the moral pain…keep pulling the rope, or you can drop the rope.<br><br>Now imagine you letting go of the rope. Opening up your palms. Laying them gently in your lap or in front of you as you imagine yourself dropping the rope. Opening up your body posture…relaxing your jaw. Letting go of the fight against your moral pain in this moment.<br><br>In the moment that you drop the rope…what emotions do you feel…what sensations in your body…what urges do you have, what thoughts do you notice…what is your mind telling you about the tug of war? See if you can make room for these experiences as you notice them…letting them exist without pulling against them…<br><br>(Notice if you have urges to pick up the rope again and see if you can let that urge exist inside of you without acting on it)…<br><br>With your hands laying gently not pulling, take a moment to notice your surroundings…that there's more than the deep dark pit…the rope…and the moral pain you are fighting.<br><br>Notice dropping the rope didn't make the monster/form go away…but something important is happening here.<br><br>Notice that your hands are in your lap. They are free from the rope. What are you free to do with free hands? What are you free to do when you aren't busy pulling on the rope?<br><br>With free hands, is it possible to begin to live your values? You are free to connect to what matters to you… family… friends… work… spirituality… this group? Notice that your moral pain will still be present – it's still on the other side of the pit…it's an experience beyond the pit, that you will always carry inside of you…one that symbolizes your humanness…that you feel moral pain for the violation of your morals…what you stand for…one that will exist as you live your values…one that will call out to you to pick up the rope. It will be there…but you no longer have to pick up the rope. You no longer have to be defined by this battle. It no longer must define how you live your life. The rope…the urges to fight with the pain…to control it…to avoid it will be there too… but you are now aware that you can choose to drop the rope when you notice yourself grabbing it… you can choose to walk with your moral pain toward what matters most to you. Imagine yourself walking toward the things you care most about.</td></tr>
<tr><td colspan="2">Processing</td></tr>
<tr><td>Now pause and open your eyes…<br><br>What did you notice in dropping the rope? What moral pain, if any, showed up? After you dropped the rope, what directions were you free to walk towards? What values guided you? Did you notice yourself picking up the rope again? What happened when you picked it up again?</td><td>If needed clarify that "dropping the rope" doesn't make the pain go away. It's instead about how we choose to relate to the pain.<br><br>Highlight that picking up or dropping the rope is a choice we all make each moment</td></tr>
<tr><td colspan="2">Experiential</td></tr>
<tr><td colspan="2">Now one last time, close your eyes… As you reflect on this scene you've witnessed of the you in the tug of war and them dropping the rope… As you stand back and look at that version of you caught in the</td></tr>
</table>

| | |
|---|---|
| *tug of war… that you that has pulled on the rope for a long time and will want to pull on the rope again in the future… is there anything you would say to that you? Is there anything you want to do for this you that gets stuck in battling their own moral pain? If you could show this you stuck in the tug of war anything, what would you show them? …Imagine now that you are saying, doing, showing them whatever it is that you would like to share with them…making space in your heart for the you that still gets caught in this struggle…noticing that this struggling you is a part...but not all of you.* | |
| **Processing** | |
| *Now pause and open your eyes…*<br><br>*What would you say to that you in the tug of war? What would you do for the you battling their moral pain if you could?*<br><br>*What did you notice when asked to make space in your heart for the you that is struggling with your moral pain?* | Begin eliciting self-compassion. If members respond with more punitive responses, acknowledge they're having these experiences and invite them to hold them in their awareness. |
| **Generalize** | |
| *What does the tug of war look like for you in your life outside of this session? What are you doing to fight and control moral pain? What might it look like to drop the rope? How might you remind yourself to drop the rope?* | Help clients translate the metaphor into their day to day lives. |

**Bridge to Discussing Values and Practice Assignment**
Using group's awareness of the function of behaviors consistent with moral injury and costs of avoidance, pivot towards committed action as an alternative approach to moral pain

*Together…we'd like to spend some time exploring what kinds of things would you be doing instead of the tug of war.*

*In dropping the rope, it frees us up to practice living our values…even while also experiencing moral pain. A big part of the way we understand recovery from moral injury is acting on your values even when you're experiencing painful emotions and thoughts. Throughout this group we will be inviting you to take steps that move you toward those areas of life that matter most to you (even while you are experiencing pieces of moral pain-- your thoughts, feelings, urges, and sensations). This is how we turn a value into an action.*

**Review and Process Practice Exercise from Previous Session**
*Last week we practiced a values card sort. That exercise is designed to help each of you begin to consider what is important to you and how you can work to live that throughout this group and life. You can think of values as a kind of compass heading that gets your feet pointed in a meaningful direction. But if our focus is on avoiding moral pain, we can do things that move us away from our values and keep us stuck in patterns of suffering and disconnected from relationships, from spirituality, from work, our care for ourselves. We want to connect you to meaning again moving toward what matters to you individually and as a group together.*

| **Processing** | |
|---|---|
| *As you participated in the values card sort what did you notice mattered the most to you?*<br><br>*In what areas of your life do these values show up?*<br><br>*Was that expected? Did anything about the values card sort surprise you?*<br><br>*What thoughts and emotions did you notice while doing the values card sort? How did you respond to these experiences?* | Ask about relationships, spirituality, wellbeing, employment<br><br>Attend to any responses consistent with moral pain presenting while engaging values. Identifying that pain can arise in the context of valued action (even just by thinking about valued domains) AND reinforce engaging in that value in the presence of that pain. This can help to lay the foundation for the values pain connection. |

**Bridge to Skills Practice and Review Moral Emotions Handout**
*As you saw in this exercise, emotions can arise from considering what matters most to us. We will spend a lot of time together exploring values and the emotions and other experiences connected with them. This could include making room for pleasant moral emotions, even if we find them uncomfortable. Because these emotions are so common in moral injury, we want to take a moment to create some shared language, so we better understand each other's experiences.* [Direct group's attention to Pleasant and Painful Moral Emotions figure in Workbook]

*We can note that these moral emotions can be pleasant or painful.  When we encounter things that depart from our values, we might feel guilt or anger. When we encounter things that support our values, we might feel caring or inspired. Moral emotions can also be emotions we feel towards ourselves (like shame or pride) or ones we feel towards others (like contempt or gratitude).*

*We're inviting you the make room for all of these experiences and both inside and outside the group and we will be practicing noticing them and learning how to relate to them in ways that allows you to live your values.*

**Skills Practice**
*This week we'd ask you to pay attention to when you find yourself in a tug of war with moral pain. Consider how being in this tug of war, affects how you're able to live your values. If you find yourself stuck, see if you can pivot toward your values while making space for any emotions, thoughts, urges, sensations, and memories that arise- allowing them to be present. Please come to group next week with an example of this to share.*

*This coming week, we'd also invite you to start exploring possible ways to live your values. We want to begin shifting away from strategies that haven't worked and try something different. Before next session, we're asking you to make a list of activities that you think would be meaningful to you. This will just be a start and you can add to it as we go along. Reference your values card sort for examples of areas you care about and how you want to try living these values* [Review with group how to fill out Seeing Possibilities worksheet in the workbook].

**Post-Session Clinician Review**

- Check-in internally with experience of session and reflect on personal or clients shifts that were noticed.
- Identify potential patterns of behavior observed to be aware of in future sessions.
- Compassionately identify any self-care needs that became evident during the session and, if willing, make a plan to address them as soon as workably possible.

# SESSION 4

# THE MORAL PAIN-VALUES CONNECTION

## CLINICIAN ORIENTATION AND PREPARATION

### Purpose of Session

This session's purpose is to continue generating motivation for interacting differently with moral pain. Instead of trying to control the moral pain in ways that lead to paradoxical and costly effects, this session encourages clients to look at moral pain as a guide for living well. The session helps clients establish the link between values and pain so that the client is able to see that they not only "hurt where they care" but that awareness of this connection also begins to place them in contact with what they are missing (what matters to them) and why they are missing it (attempts to avoid and control pain). Values work is held out as what guides the Veterans to live more meaning lives rather than remaining stuck in the battle with moral pain. Values are a source of positive reinforcement rather than the negative reinforcement of avoiding discomfort.

### Materials Needed

- ACT-MI Workbook
- Piece of paper (roughly 3 x 3 inches, the size of a sticky note)
- Pen or pencil

### Pre-Session Clinician Preparation

- 1-minute mindful check-in with self (noticing own contact with the present vs. conceptualized pasts and futures, potentially including those about group members)

## SESSION CONTENT

### Bridge from Previous Sessions

*Last week through the tug of war exercise we explored more how trying to control or fix moral pain actually just keeps us stuck. We'll build on that understanding today by exploring how dropping the rope and making room for the moral pain can free you to live with more purpose meaning.*

*To begin, we're going to recreate the tug of war and notice what it's like to drop the rope, but instead of doing it imaginally, we're going to keep eyes open and have you to take one of your hands and grip your other wrist.* (If providing group virtually invite group members to unmute so they can respond in the moment to Clinician questions). *The arm that's doing the gripping represents you. The arm that's being gripped is the rope. I'll be giving some direction about what each arm is going to be doing. You're going to be doing some pulling on your own arm to represent the tug of war but please be aware of your own limits and don't injure yourself.*

| Example Clinician Language | Notes for Clinician |
| --- | --- |
| **Experiential** | |
| *Start by having one hand grip the other wrist and start to pull gently on the other arm…the "rope"… As you pull on the rope,* | |

| | |
|---|---|
| *the rope itself stays still so even though you're pulling, nothing actually moves… Now with the rope remaining still, pull a bit harder… … notice what's happened to the sensation… what it feels like….* | The goal of this exercise is to help the group experience a physical analog for the psychological act of avoidance/control. |
| **Processing** | |
| *Stay in the tug of war but say out loud what this is like…*<br><br>*Now drop the rope…. What immediately changes in your experience?… Sit here for a moment… How is it different to be letting go rather than pulling?...* | Emphasize experiences of strain, effort, and discomfort.<br><br>Highlight the contrast (reduced struggle, more freedom). |
| **Experiential** | |
| *OK, pick up the rope again and start pulling just like before… pull hard enough that you start to feel some strain… what are you experiencing now as you're right back in the tug-of-war?...*<br><br>*Notice the energy being put into the struggle… How long have you been pulling?... How much longer do you want to keep pull?… Now let go and stay let go… notice the change again … notice what <u>hasn't</u> changed? The "rope" is still around, still there to pull on if you chose to, but if you leave it where it is, notice how your other arm and hand are open and free. Not "free of" the rope (it didn't go away), but "free to" move and act differently…*<br><br>*Pause here for a moment and let's imagine together what you could do with that freedom that could bring more meaning and significance into your life…* | Emphasize comments of familiarity or irritation at being back where they started. Amplify comments that may indicate that pulling is unhelpful.<br><br>Transition to review of "Seeing Possibilities" practice exercise |
| **Clinician Note:** If necessary, quickly correct clients' understanding of the Tug of War metaphor to reflect that it symbolizes being stuck in trying to control or avoid moral pain, even when it prevents you from living your values.<br><br>Other points that should be clarified if needed include:<br>- The rope represents any and all costly strategies (alcohol, suicidal ideation, isolation, aggression, overworking) us to avoid/control our pain (guilt, betrayal, anger)<br>- Dropping the rope symbolizes learning to relate differently to the moral pain, so we aren't controlled by it anymore (i.e., allowing the pain to exist and not immediately reacting to it)<br>- Reinforce the connection between letting go of control and the freedom this creates for the client to pursue values-consistent activities | |

**Review Seeing Possibilities exercise**

*This freedom to pursue meaning and purpose in our lives is what dropping the rope is all about. Last week we asked you to being thinking about what that might look like in practice by completing the Seeing Possibilities exercise.*

| **Processing** | |
|---|---|
| What possibilities for living values did you identify?<br><br>What values were they connected to?<br><br><br><br><br>What did you notice happening inside yourself as you looked at these possible ways to live your values? | Clients often struggle to identify new ways to live their values and feel pressure to pick the "right" behaviors. Encourage instead an attitude of curiosity and exploration.<br><br>If needed can acknowledge that considering values may have also brought up pain |

**Introduce New Content**
*Today, we are going to dive a bit deeper into what dropping the rope can free you up to do. We will explore both the moral pain you will encounter if you choose to stop fighting, avoiding, controlling, trying to change your experiences in the tug of war and we will also explore the directions you will be free to head if your energy isn't consumed by the battle.*

*We'd like to ask you to get a piece of paper the size of a sticky note. It could be a post-it, an index card, a scrap of paper, and something to write with.* (If conducting group in person, Clinicians should bring these materials)

| Example Clinician Language | Notes for Clinician |
|---|---|
| **Experiential** | |
| *We'd invite you to close your eyes and for a moment remember the tug of war scene we connected with a few moments ago. Bring to mind the rope and the monster…the moral pain that you were pulling against. What emotions…thoughts…sensations…urges are represented by that that monster for you? What emotions…thoughts…urges does* [name objects from initial tug of war exercise] *…the memory of your experience of the moral violation hold? What are parts of your moral pain that you struggle against the most in the tug of war? What do you pull on the most?*<br><br>*Open your eyes for a moment and write down a few words that symbolize the moral pain you find the hardest to hold or experience… the pain that makes it the most likely that you will pick up the rope and start fighting again. Maybe it's an emotion, a thought, an urge, a sensation… maybe it's all of these experiences at once. Choose a phrase or a few words that seem to fit this experience and write them on one side of the paper. Raise your hand briefly when you've finished writing.* | Naming the objects that clients chose to symbolize their moral pain helps the exercise be more personal and evocative.<br><br>Clients should only write a few words at most. If clients appear to be writing sentences, interrupt and redirect them. |

| | |
|---|---|
| *Once you've written down a few words that capture this pain, see if you can become aware of this pain…where in your body do you tend to experience it? How do you know when it's there? See if you can locate its shape…temperature. What does your mind do in response to it? What do you feel pulled to do with this moral pain?* | |
| **Processing** | |
| *And now we'd invite you to open your eyes and bring your focus back to the room.*<br><br>*What words did you use to describe your experience of moral pain?*<br><br>*What do you notice now about your moral pain? What does it look like? How has it shown up now?* | Goal is to help clients notice and interact with their moral pain rather than trying to avoid or control it. |
| **Experiential** | |
| *Holding this piece of paper in your hand, we'd invite you to close your eyes again. As you notice the pain that you have written down, notice for a moment that there's another side to this piece of paper. We'd like for you to consider if this pain you've noticed might also be connected to something you care about. Ask yourself, why does this pain matter to me?... Why is it important?...  This pain signals that there was a violation of some part of your morals…What is the pain trying to fix?... What does your pain want?... Maybe there are values or morals that were violated that this pain is pointing out?... What matters about this pain?... What words would you give those values?*<br><br>*Now keeping your focus on the exercise… We'd ask you to open your eyes and write down a few words that describe the value or values linked to this moral pain on the blank side of the paper…*<br><br>*Once you've done this, hold the paper in your hands. What does it signal about your moral pain that it is connected to your values? What does the presence of this pain tell you? What's its purpose?*<br><br>*As you reflect on these values, what do the values point your toward? How do they help show you what matters to you in your relationships, spiritual practice, ability to take care of yourself, in your profession? Imagine that you were free to live them, what might you start doing?* | Goal is to help clients identify the values implicit in their moral pain<br><br>Slow down during this portion of the experiential in order to allow clients a chance to experientially sense what these implicit values might be.<br><br>Again, client should only write a few words. If clients seem to be struggling to identify words, gently explore what important value was violated by the morally injurious event(s) but be careful to not delay the exercise. It is better for the client to write down an approximation and continue moving forward. |

<table>
<tr><td colspan="2">Processing</td></tr>
<tr><td>And now we'd invite you to open your eyes and bring your focus back to the room.<br><br>What words seemed to signal the values connected to your moral pain? What did you write down on the other side?<br><br>What did those values point you toward? What would someone acting in a [value label] way, be doing?<br><br>You are the one who is holding this pain. And you are the one holding the values on the other side. Because you hold it you have a choice about how to live with both sides of the paper.</td><td>Clients may express sadness or disappointment at the seeing the connection between their moral pain and values. If needed, clarify that this connection relates to their moral pain about the event, and not the event itself. In other words, the pain only tells them that they care, NOT that the event had to happen or it was or wasn't justified in happening.</td></tr>
<tr><td colspan="2">Experiential</td></tr>
<tr><td>Given the connection between pain and what you care about I'd like to ask you all…Can you get rid of one side of the paper? Is it possible to wipe out the pain and still care…and still have the value?... Can you care about the value without the pain? That you have pain means you care…and to care means a willingness to have pain. But when we are stuck in a battle with these emotions…we are often disconnected from how we want to show up in our relationships and other valued areas.<br><br>Notice how we can't separate these from each other. If we try to cut the paper it would just destroy both. If we stop hurting, we stop caring. How does this relate to any of your past relationships? Have you ever been able to care about someone without experiencing the possibility of pain? Every relationship comes with pain…If you care about connection…it means being willing to have pain.<br><br>In order to live your values, you need to also make a choice about how to respond to the pain you open yourself up to you are caring about other people. This group is about being able to live your values which could mean making space for the pain, so that you have the freedom to live meaningfully. In this group, we aren't asking you to practice noticing pain for the sake of it…we are practicing noticing moral pain together to create more opportunities for you to choose how to live your lives…in this room, but also outside of it.</td><td>Acknowledge the inseparability of pain and values and that this exercise isn't about prioritizing one over the other. If needed explicitly use the two-sided paper to make this explicit. The pain might be "offended" or "indignation" or "swept under the rug" and the associated values "accountability" or "integrity" or "responsibility"<br><br>Emphasize and deepen the experience of creative hopelessness as needed. (How has trying to separate your pain and your values worked for you in the past? What has it cost you?)<br><br>The goal is to enable clients to honor their pain in ways that allow them to pursue multiple values. For example, rather than trying to honor pain with more pain (i.e., self-punishment), explore what the costs are to that approach and if there might be other, less costly ways to honor moral pain (e.g., living the value connected to the pain).</td></tr>
<tr><td colspan="2">Processing</td></tr>
<tr><td>You all just held your pain together… and contacted what matters to you together…. as a community. What did you</td><td>Guide group in recognizing the aspects of their experience that</td></tr>
</table>

| | |
|---|---|
| *notice about each other's pain? What did you notice about each other's values?*<br><br>*What are the costs of holding your pain alone?*<br><br>*Notice how our ability value and care for one another helps us each hold our pain together. It also helps us connect with what matters together.* | allow for empathy and a sense of common humanity with each other.<br><br>Explicitly identify how social communities facilitate connection to values and provide support for holding moral pain. |
| **Experiential** | |
| *We'd invite you to hold the paper in your hand…looking down at it….pain side up. You literally are holding your pain in your hand in this moment. You have the pain in your hand. Because you are the one holding it…it doesn't have you. Notice that you are making the choice to hold your pain. It's an experience that you can choose to be aware of and notice, just like any other experience. It may be uncomfortable, but there is a You here now than can hold your pain even while experiencing discomfort.* | Use two-sided paper exercise to implicitly introduce the observer-self (self-as context). |
| *See if you can notice the pain right now…or the last time you felt it…what do you notice about it? Where is it located? Notice if you have the urge to pull on the rope... if there's an urge to avoid it or control it or distract from it…and bring your attention back to observing your moral pain. See if you can notice the rise and fall of that pain…and how you are holding that pain…how you are holding the paper…if you are willing, try and open up your palm to it…open up your body to that pain…holding it gently for what it is…as an experience that exists inside of you and that is part but not all of you.* | Revisit and reinforce creative hopelessness and help them move into a posture of willingness and acceptance |
| *Now take a moment to consider again what that pain signals you care about…what's its meaning?* | Reinforce pain-values connection |
| *Turn the paper over to the other side…look down at it and then see if you can locate that value inside of you too…maybe a memory of a time you felt like you were truly living that value. Maybe it was long ago…or recently. As you consider living that value what emotions…thoughts…sensations…urges…do you notice? Notice how you are holding this experience of your value too…opening up your palm and body to the possibility of living a life in this moment that is connected to meaning.* | Turn client toward committed action by connecting the value to concrete actions |
| **Processing** | |
| *Consider how you might take a step toward this value. See if for a moment, you can picture yourself moving in the direction of this value.* | Elicit examples of possible committed actions from clients |

<table>
<tr><td>What is a small step you can take on a path of living this value? What does it look like to act on it? What are you doing?<br><br>What pain do you expect to show up as you live this value? What thoughts? Emotions? Sensations? Urges?<br><br>How might you respond to this pain while pursuing living your value?</td><td>It is very common that clients have trouble imagining new ways of living. If needed, ask them to think about times they’ve seen others act out these values or what a “fly on the wall” would hear or see if this value was being acted out.</td></tr>
<tr><td colspan="2">Clinician Note: Effective committed actions at this stage are generally small and do-able. The goal is for the clients to drop the rope for one moment and take a step in the direction of their values.<br><br>Pay attention to the apparent function of committed actions. It is <u>not</u> our goal to resolve or expunge the moral pain or repair or the morally injurious event. Rather, the action must be done for the sake of the value, which has inherent worth to the client for its own sake, regardless of the impact that action has on their moral pain. For example, a client may choose to attend a religious ceremony such as Catholic confession. This might be done to live the value of integrity or humility, not for the purpose of stopping the emotions of guilt or shame from being felt.</td></tr>
</table>

**Bridge to Practice Assignment**

*From now until the next time we meet, carry this paper around with you. Carry it in your pocket…your shoe… take it out…each day. Making space for each side of the paper. Set aside a few moments each day (can use alarms on your phone) to intentionally turn your attention to the paper. Notice how you are carrying it. Take it out of your bag or pocket or shoe. Look at one side…then the other. Notice how the sides are connected…one side can’t exist without the other. Notice any urges to avoid content on the paper, and bring your attention to that content, notice how it feels in your hand…how you are holding it…noticing that the experiences on this paper are part of your experience…but not all of you if you can be the one holding it….opening your hand to both sides of it…allowing it to be there with you.*

As you carry this paper, we would also like you to continue exploring new possibilities in your life… areas of meaning that you might be able to experience or expand. You might consider for example, if there are ways you can put the value on your two-sided paper into action.

**Post-Session Clinician Review**

- Check-in internally with experience of session and reflect on personal or clients shifts that were noticed.
- Identify potential patterns of behavior observed to be aware of in future sessions.
- Compassionately identify any self-care needs that became evident during the session and, if willing, make plan to address them as soon as workably possible.

# SESSION 5

# SEEING AS AN OBSERVER

## CLINICIAN ORIENTATION AND PREPARATION

### Purpose of Session

This session's purpose is to present acceptance as the alternative to control. This is done by teaching clients how to be an observer of moral pain that has the choice to hold pain without being consumed by it. Clients are further encouraged to make room for these experiences by holding them with gentleness. When this is established, Clinician's introduce that clients can choose to open up oneself up to painful experience in order to have the opportunity to live values in the here and now.

### Materials Needed

- ACT-MI Workbook

### Pre-Session Clinician Preparation

- 1-minute mindful check-in with self (noticing own contact with the present vs. conceptualized pasts and futures, potentially including those about group members)

## SESSION CONTENT

### Bridge from Previous Sessions

*This past week, we asked you to practice dropping the rope and intentionally carrying your moral pain (represented by the piece of paper) and finding ways to live the values it signals. To start today, we're going to practice noticing and holding pain and values as an observer who has the choice to notice these experiences. In a moment, we're going to ask you to remember the two-sided paper you made last week and the thoughts and emotions you experience connected to it. Rather than trying to do something with it, we're instead going to ask you to practice the skills of noticing and observing.*

| Example Clinician Language | Notes for Clinician |
|---|---|
| **Experiential** | |
| *We'd invite you to get in touch with what you wrote down on the paper. If you have it, you can look at it for a moment. If not just remembering it. Close your eyes and visualize the pain and the values that the piece of paper represents.*<br><br>*Recall the words you gave the pain you wrote down on one side?... What did that pain want? What did it signal mattered to you…what values did you write on the other side?... Just hold the image of the paper values on one side…pain on the other…in your mind. Notice it. Now following our session last week, call to your mind what you chose to do with the two-sided paper. Where did you physically put it? Did you leave it in the same place? Did you hold it or handle it during the week? How did you interact with it?… It might be that remembering the two-sided paper is itself bringing up some moral pain in* | Even if clients effectively connected to this experiential exercise during the previous session, they will likely need time again to reconnect with it now again in the present. Be careful not to rush. |

| | |
|---|---|
| *this moment. Just note what that pain is... and how it shows up for you... Allowing your experience to be as it is…making room for it...* | |
| **Processing** | |
| *Now staying in touch with the scene you've been observing, gently open your eyes and rejoin the group…*<br><br>*Can you notice your pain here now? What do you observe?*<br><br>*Who is observing that emotion…thought…urge…sensation?*<br><br>*Can you notice your values here now? What do you observe? Who has care for that value? Who does it matter to?*<br><br>*Who holds your pain, values, and your memories?*<br><br>*If you can hold the memory, the paper, the pain, the values, do these experiences completely define you? Are other group members defined by their paper? (Is [Name] his paper? Is he his moral pain??*<br><br>*If not…then are you your paper? Are you, your moral pain?* | By including questions that foreshadow future ACT-MI skills (in this case the observer self) clinician's can begin to indirectly develop psychological flexibility and lay the groundwork for future sessions.<br><br>These questions help prepare clients for the more evocative, and potentially more challenging, experiential exercise later in the group. |

*We're going to practice connecting with the part of you that can hold all experiences…no matter how painful…or how pleasant. In today's group we will practice connecting with the part of you that has the ability to observe these experiences so that you have the choice to drop the rope and live your values even when pain shows up. If you can observe and notice, you have space to choose your behavior.*

**Introduce New Content**

*We want to help you get to a place where you can choose to live the values that your moral pain points to more deliberately and fully. Part of that work will be how to hold all your experiences rather than grabbing and pulling on the rope…what do I do….what does that actually look like? We are going to do some work with that.*

**Experiential Exercise**

- Conduct experiential and process elements
- Respond to challenges in comprehension or application of exercise
- Generalize skills to outside of session

| Example Clinician Language | Notes for Clinician |
|---|---|
| **Experiential** | |
| *To start this exercise, we'll have you close your eyes. We're going to have each of you build an image. After we're done* | In this section, we set the context for teaching the observer self by first reviewing why efforts to |

| | |
|---|---|
| *creating this image, we'll have a chance to talk about it together.*<br><br>*Imagine that all of your internal experiences are like pieces on a chessboard— on one side are all of your painful thoughts, emotions, and memories …and on the other side are all of your pleasant thoughts, emotions, and memories (if you don't play chess, you can imagine a sport, with two teams playing on a field). And just like in chess, we can become really invested in one side or the other winning. The pleasant experiences trying to outthink, overpower or eliminate the painful experiences… somehow trying to win this internal game of chess. But notice how our minds are always generating more thoughts, emotions, and memories… so even as we push out a painful experience in one moment, our mind creates a new one the next moment. Sometimes even focusing on happy experiences can remind us of times we've felt sadness. Because our mind constantly creates new experiences, it makes it impossible for the pleasant to completely and permanently eliminate the painful… and so this battle never ends.* | directly control moral pain often backfires and paradoxically maintains or intensifies suffering.<br><br>By emphasizing the unworkability of direct control over internal experiences, clients are likely to be more open to considering alternatives (such as the observer self). |
| **Processing** | |
| *[eyes open]*<br>*What are the painful and pleasant pieces on your board? Notice if they take certain shapes or forms for you.*<br><br>*How long have they been there? What have tried to get rid of them? How has that worked for you?*<br><br>*Where are you in the scene? What are you doing*?<br><br>*Keep your eyes open but try to stay in contact with those pieces as we ask you some more questions. We know that each of you have these pieces, but what else is here? What else do you have? [elicit and shape responses to .*<br><br>*If group members identify the board, ask follow-up questions to expand such as,*<br><br>*How is the board different from the pieces?*<br><br>*What is the board doing that is different from what the pieces are doing?*<br><br>*How would you acting more like the board be different than how you've interacted with your pain in the past?* | Watch for control-oriented responses and guide the client back to creative hopelessness as needed.<br><br>In this section of the exercise, you are trying to help the client(s) shift from an active struggle against the moral pain, to the broader, transcending perspective of the observer self. Try to imbue your language with a spirit of curiosity and experimentation, so that client(s) will follow. |

| | |
|---|---|
| **Clinician Note:** If the board isn't identified by group members, identify it for them by saying something similar to, "*We sometimes take for granted that there's a board that olds the pieces… it makes space for them…in all directions…but it's not involved in the battle at all. The board can observe the pieces…"* | |
| **Experiential** | |
| *What if, instead of being locked in this eternal battle between pleasant and painful pieces…, we could become aware that, just like the board, we also hold everything? Just like the board holds all the pieces, there's a You that holds all your experiences…that You has always existed and has held all your experiences across time… This is the part of you that is the holder of all of the pieces … making space for them… We call this the Observer-Self because it's the place that we see from. It other words, the observer-self is not a thought, emotion or memory. Rather, it's the place where all of your thoughts, emotions, memories, urges, sensations occur.* | Be careful to use language that captures the essence of the observer self… the part of ourselves that can be aware of our own experiences but because it can see and notice them is also not defined by them. |
| **Processing** | |
| *What experience did people notice showing up on their chessboard?*<br><br>*Is [Name] her chess piece. Is she only that. What else is [Name]?*<br><br>*[Name] are you only your chess piece?*<br><br>[Practice and reinforce the observer self with such questions and prompts as…]<br><br>- *So, there's a You there that's aware of your [experience]…*<br>- *Notice who is having that experience…notice who is holding that experience?*<br>- *Who knows that there's a part of you is feeling… hearing… and seeing yourself… just like it feels, sees, hears, and see's other things…*<br>- *The observer-self doesn't make the pain go away, but it holds the pain*<br>- *These IS NO experience without a BOARD to hold them (no piece without a place for it to be* | It is very common that clients will initially struggle to understand and connect with the observer self. They will often try to look AT it, as if it were an object or an experience outside of themselves. Instead, the observer self is the place we, as conscious beings, look FROM. For that reason, we can never truly see it, like we would a thought or an emotion. Rather, we can only become aware of the process of us noticing our own experience in any given moment. This ongoing awareness of ourselves as observers of our own experience is the observer self. Because this is a new way of approaching their experience, clients will likely need repeated reminders, from various angles, across multiple sessions to grasp and intentionally use the skill of connecting with the observer self. |

**Bridge to Practice Assignment**

We'd invite you to practice this same skill in your relationships and social interactions before the next group session.

**Skills Practice**
*We invite you to take a few minutes before an expected interaction (with a spouse, partner, child, or coworker). Call that person to your mind and imagine what thoughts and emotions they might be having in that moment. Ask yourself is that all of who they are? Are they completely defined by those particular thoughts and emotions, or are they something more? Now notice for yourself, what thoughts and emotions are you having in this moment? Are you completely defined by those particular thoughts and emotions or are you something more? Whatever your mind says in response those questions, notice who is having those experiences.*

*Then move forward with that expected interaction trying to stay in contact with the sense that both you and this other person are more than their thoughts and their emotions. Notice if that perspective changes how you interact with this other person. How might it provide you with new or different ways to relate to them? Notice also if it changes how you relate to yourself and the options you see for how you might act.*

**Post-Session Clinician Review**

- Check-in internally with experience of session and reflect on personal or clients shifts that were noticed.
- Identify potential patterns of behavior observed to be aware of in future sessions.
- Compassionately identify any self-care needs that became evident during the session and, if willing, make plan to address them as soon as workably possible.

# SESSION 6

# HOLDING MORAL PAIN

## CLINICIAN ORIENTATION AND PREPARATION

### Purpose of Session

This session's purpose is to help clients deepen their awareness of the observer self and begin to use this skill to hold their moral pain more flexibly. To help support the development of this skill, an experiential exercise is used where each group member holds a physical representation of their moral pain. Through engagement with this physical object and the use of guided perspective taking, clients are shown through their own experience how they are not defined by their experiences, including their moral pain, thereby creating greater opportunities for the flexible living of values.

### Materials Needed

- ACT-MI Workbook
- Writing utensil and paper (If possible, have client collect before starting session)

### Pre-Session Clinician Preparation

- 1-minute mindful check-in with self (noticing own contact with the present vs. conceptualized pasts and futures, potentially including those about group members)

## SESSION CONTENT

### Bridge from Previous Sessions

*First, we would like to check in about moments that were really meaningful this week. If you noticed times that you were living your values, what did you do to connect with meaning? What skills did you use to do it?*

### Review Practice Assignment from Previous Session

*Last week for practice we invited you to spend some time before a social interaction intentionally noticing how the other person and yourself were both holders of their experiences, like two chessboards, each holding their own pieces. If you didn't complete the exercise, we'd invite you to practice it right now as we discuss together. Notice that everyone here and now in this group has their own chessboard, with pieces appearing and moving around on them.*

| Example Clinician Language | Notes for Clinician |
|---|---|
| **Processing** | |
| *What did you notice when you tried to be aware of other people as having their own chessboards and pieces this past week?*<br><br>*What did you notice for yourself?*<br><br>*Where there any pieces that you got stuck on*? | Help clients recognize that <u>all</u> internal experiences (including those occurring right then in session) are examples of "chess pieces" |

<table>
<tr><td>[If needed] "Let's slow this way down and take it frame by frame… that's a piece… that's a piece..."<br>Isn't it interesting that each of you have millions of other experiences each day? Sights… sounds… thoughts… memories… and we choose to let many just be… as they are…some we don't even really notice. Some others… the particularly painful ones… we can focus on… and this can end up consuming our time and energy.<br><br>How is it that among these millions of different thoughts… emotions… sensations… memories… experiences we have each day… that we become so attached to some of these experiences but not others?</td><td>Group members may give reasons why it's harder to not control moral pain, such as:<br>- This pain is bigger/more powerful than the other pieces<br>- If I give up fighting… (it will defeat me/I'll betray my values)<br>- If I let the guilt just be there… (I'm condoning what happened/it's unfair to not suffer)<br><br>Clinicians can help the clients recognize that even these reasons are themselves "pieces" on their "board"</td></tr>
<tr><td colspan="2">Clinician Note: Practice <u>in session</u> by helping group members recognize that all their experiences, (including experiences that are focused on controlling/fixing their other experiences) are all "pieces on their board." If clients present arguments or stories, simply ask them, "Who is noticing that thought right now?" to demonstrate from their own experience that the observer self is always present and implicit in their own experience.</td></tr>
<tr><td colspan="2">Experiential</td></tr>
<tr><td>Notice how even these thoughts and urges are themselves chess pieces, in other words they are just more experiences. What if, instead of reaching out and grabbing these thoughts as if they were the only thing that mattered, you could let the thought just be a thought?... No more… no less. Let's briefly revisit the chessboard scene that you each created last week and include these new experiences as well. Our goal is to be able to notice both our moral pain AND the thoughts, emotions, and urges we have <u>in response to that pain</u> and allow them to be without acting on them.<br><br>Being aware of our experiences from the perspective of an observer allows the opportunity to hold our painful thoughts, emotions and memories without being drawn into and consumed by them. It also opens up new ways of relating to ourselves and others around us. Today we want to practice doing this in a very concrete way. In this next exercise we'll be specifically focusing on some of the emotions and thoughts you have about your moral injury and how you hold these experiences. Our goal will be to see those thoughts and emotions as thoughts and emotions, as chess pieces, that we can notice and hold without having to act on them.</td><td>Urges that clients might have in response to their painful chess pieces might include<br><br>- I want to knock the pieces off the board<br>- I want to blow the board up<br>- I want to shoot the board into space<br>- I have to keep sacrificing pawns to not lose<br><br>Clinicians can again help clients see that these urges are themselves also pieces of their experience that can be noticed and observed, rather than acted on.<br><br>The very act of noticing a thought, emotions, urge or sensation implies a noticer who is to some degree distinct and separate from the thing being noticed.</td></tr>
</table>

<table>
<tr><th colspan="2">Experiential</th></tr>
<tr><td>[Provide basic instructions to help clients enter a mindful mental posture] Recreate in your mind the chess board we constructed last week. See it extending in all directions… Holding all the pieces… but itself not being changed by them…On one side see the pieces you had created to represent the morally painful thoughts, emotions, sensations and urges. On the other side see thoughts set up to try and control or battle the pain. Now imagine these new pieces we’ve just discussed… the reasons your mind gives you about why you need to control or avoid your pain… see each of those reasons, those urges to control… as pieces coming down, landing, and being added to the game that’s already underway.<br><br>Now, if you haven’t already done so, reconnect with the perspective of the board… your observer-self. The you here now that is the holder of the experiences but is not the experiences themselves. Just as the board is not the pieces… You are not your pain… you are not your thoughts about your pain… you are more than any one emotion, thought, memory or sensation… Your observer self is the part of you that observes all of those things yet is none of them…a continuous observing you that has existed across your entire life.</td><td>Clinicians can make specific mention of some of the reasons and urges mentioned previously and weave them into the language of the exercise</td></tr>
<tr><th colspan="2">Processing</th></tr>
<tr><td>As you connected with the observer-you, what pieces did you notice coming down onto your board?<br><br>Have these pieces been there before? Are the familiar?<br><br>What’s different about experiencing them from the perspective of the observer you?<br><br>[If fusion/self-as-content is happening, use questions such as]<br><br>- Who knows that there’s a part of you that is thinking…feeling… wanting…?<br>- So, there’s a You there that’s aware of …<br>- Notice who is having that experience…notice who is holding that experience…<br>- Notice the same You is having this experience that had [ _ ] experience. The same You is talking now that noticed [ _ ].</td><td>When discussing the observer-self, stay attuned to the degree clients appear to maintain awareness of their internal experiences as being distinct from themselves.<br><br>This may manifest as the client saying something in an off-hand manner (as if it were obviously true) or feeling weighed down or constrained by a thought (as if it had direct power over their choices). In contrast, connecting with the observer-self often has a quality of stillness and freedom, even though internal experiences themselves may not decrease.</td></tr>
</table>

**Introduce New Content**

*Being aware of our experiences from the perspective of an observer allows the opportunity to hold our painful thoughts, emotions and memories without being drawn into and consumed by them. It also opens up new ways of relating to ourselves and others around us. Today we want to practice doing this in a very concrete way. In this next exercise we'll be specifically focusing on some of the emotions and thoughts you have about your moral injury and how you hold these experiences. Our goal will be to see those thoughts and emotions as thoughts and emotions, as chess pieces, that we can notice and hold without having to act on them.*

*If you haven't already, we'd ask you to get a piece of paper and a pen for this exercise.*

**Experiential Exercise**

- Conduct experiential and process elements
- Respond to challenges in comprehension or application of exercise
- Generalize skills to outside of session

**Introduction**

*We would like to do some work today not only noticing who holds your moral pain, but doing some work with how you actually hold it. You might notice urges to disengage from or control elements of this exercise, to even leave…we'd encourage you to notice those urges and bring your attention back to the exercise you are connecting to together.*

| Example Clinician Language | Notes for Clinician |
|---|---|
| **Experiential** | |
| *On your sheet of paper, write a sentence about something that you did or something that was done to you (maybe a betrayal) that violated your morals...Something for which you feel the most shame, guilt, disgust, anger, contempt. What you have written should get at the heart of your moral pain.*<br><br>*Now crumple the paper into a ball with the writing on the inside. We'd invite you to place the ball….your experience of your moral violation in your hand. We would ask you to practice noticing your experience of holding the thing you did or that happened to you that violated your morals. Observe your responses to it. Just noticing to yourself…what does your pain feel like in your hand in this moment? What is its weight? Take a moment to get in touch with this.* | Clinicians should pay attention from the start of this exercise how the clients relate to the piece of paper. They may treat it with contempt or show signs of disgust on their face. These may be indications of their degree of willingness to interact with their own moral pain (as represented by the paper ball). This exercise is meant to help them become aware of those patterns they've developed and contrast them with a more open, general, acceptance stance towards their own and other's pain. |
| **Processing** | |
| *Now we'd ask you to notice how you would normally relate to this pain. Before you started this group, what response would you have had to the pain you're holding now in hour hand? What would you have done with it? [Elicit responses]*<br><br>[Elicit and amplify themes of control, creative hopelessness and self-as-context] | Help clients experientially connect to the costs of control |

| | |
|---|---|
| - *How has that approach been working for you? What is it costing? If needed: Is it allowing you to connect with what matters to you?*<br>- *Who is noticing that urge/thought/emotion/piece?* | Find opportunities to help clients notice and see from the observer self, even while emphasizing other ACT-processes |
| **Experiential** | |
| *Continuing to hold the paper in your hand… notice any urges….[reflect responses from above] to get rid of the pain…the memory…to make it as small as you can in your fist…to destroy it...or hide it. For a moment, lets experience what it's like to try to get rid of the shame…the anger, see if you can squeeze the paper as hard as you can…doing everything you can to make it disappear into your palm. Squeeze harder. As you squeeze where does the pain go?* | Clients may describe increased discomfort and futility. They may also note increases in physical pain and focus and that give relief from some of their moral pain or helplessness. If the latter occurs, clinicians can revisit creative hopelessness in the next processing section. |
| **Processing** | |
| *Does squeezing solve the pain? Does it make it go away for good?*<br>*- Who holds the pain?*<br>*- Who knows you are holding it?*<br>*- If you have the ability to see it… to hold it… is it all of you?* | Elicit and amplify themes of costly control, creative hopelessness and self-as-context |
| **Experiential** | |
| *Now let's try holding the pain with an open palm. We'd invite you to open your palm and allow the ball representing your moral pain, to rest on it. Holding your moral pain in your awareness, gently opening to it. Make space for any experiences you observe. Notice that it's you that's holding the experience…the anger… disgust… guilt… shame… contempt that is in your hand right now. You may also notice that you find yourself wanting to do something with it… See if you can just notice any urges to act and bring these and any other experiences arising inside you into your awareness as you hold this pain with an open palm…letting the urges be…without acting on them* | Monitor client's engagement in the exercise and as needed offer gentle reminders or invitations to open up and allow their experiences to be, even if just for a moment. |
| **Processing** | |
| *…Now staying in touch with whatever emotions, thoughts, sensations, urges may be going on, open your eyes and come back the group.*<br><br>*What did you notice as you held your pain with an open palm? How is this different for you? What does it free you to do?* | Discuss thoughts, emotions, urges, sensations, especially those connected to unworkability and cost…<br><br>Be sensitive to signs and comments that signal the presence of pain/values and deepen these (ex. |

| | |
|---|---|
| *What happened when you squeezed as hard as you could? How well did that work? What does your experience tell you about that strategy?*<br><br>*In your life, how has trying to squeeze it away impacted the living of your values? What happens when your energy is focused on getting rid of your pain…of the memory?*<br><br>*What did it feel like to hold your moral violation? Where did you notice it*<br><br>*What did you want to do with your pain during the exercise?* | Client: *I'm afraid someone will see what's inside the ball*… Clinician: So maybe it's not just pain, but also fear….or shame<br><br>Encourage observing these experiences as experiences group members <u>have</u> rather than experiences they <u>are</u>. |
| **Experiential** | |
| [Pair all group members] *Now, we'll ask [Client name] to work with [Client name],*<br><br>*We're going to practice noticing your experiences when you hold your partner's pain, what you experience when you give them your pain, and how holding their pain is similar or different from how you experience holding your own pain. We're not asking you to share what is written on your paper with your partner or to give away your pain to this person indefinitely. We're asking you to allow them to hold your pain… shame… anger… disgust for a few moments.*<br><br>*We'd invite you to close your eyes and ask you to imagine you have given your pain to your partner. Picture yourself placing this paper with the moral violation written on it in their hand. Place it on a table or seat near you. Reflect on this to yourself for a moment…we will connect as a group in a moment so please hold your responses until then. What do you notice as you give your partner your moral pain to hold?*<br><br>*Now we'd invite you to imagine that you are holding your partner's pain. Pick the paper up again, close your eyes, and imagine this time that it represents the monster they've fought against…the hardship they've endured for their part of the suffering in this room. Notice again the heaviness of the pain that was given to you – this pain that this other person allowed you to hold. See if you can notice what comes up for you… You are holding someone else's deeply painful experience…*<br><br>[Sit in silence for a short period of time]<br><br>*…As you hold the pain of your fellow human being, notice what their pain is asking from you. Noticing what this part of* | In assigning partners, consider pairing groups members who are connecting well to material with group members who are connecting less well.<br><br>If ACT-MI is being completed virtually, the exchanging of paper balls is done imaginally. If<br><br>If touched on in previous session can name the years (e.g., *the 142 years of suffering in this room")* |

| | |
|---|---|
| *their humanity needs from you. How will you hold their shame... embarrassment… disgust… anger… betrayal… fear… contempt ? What do you want your partner to know about how you will carry their pain?* | |
| **Processing** | |
| *…Now staying in touch with the experience of holding of this other person's pain, open your eyes. We'd like to go around to each pair and have you share some of your experiences with each other and the group.* [Call on dyads one at a time and ask them the questions below]<br><br>*What do you want your partner to know about how you carried their pain? How did you want to treat their pain? What emotions, urges, thoughts, did you notice as you held their pain? How did you respond to these experiences?*<br><br>*As you gave your partner your pain to hold, what experiences did you notice? How did you respond to these experiences? What has this kind of responding cost you in your life?*<br><br>*How does the experience of holding your partner's pain compare to how you held your own pain? Is there a different quality about how you are holding it? Is there more openness…? As you held their pain did you see it as defining them, is it all of them?*<br><br>*They are more than their pain…is [Name] his moral violation?*<br><br>*Are you more than yours? Does this same perspective extend to you?* | Clients will often express a willingness to hold other's pain with respect, and even reverence, while at the same time, believing that others will experience holding their pain as a burden. This inconsistency can create a powerful opportunity for clients to begin valuing their own experiences, including moral pain, as part of their humanity.<br><br>If needed, clinicians may need to clarify that allowing another individual to hold your moral pain is not the same as giving it away. Each person retains ownership over their moral pain and there is no expectation that they will be relieved of it forever. Rather, in this exercise, sharing the paper ball represents our ability as humans to hold pain together in the context of our social relationships. |
| **Experiential** | |
| *Now we invite you to give back your partner's moral pain. You are now holding your own moral pain your own moral violation again, in the palm of your hand. We'd invite you to close your eyes. Reconnect with the experience of holding your own pain. Notice the rise and fall of your emotions and thoughts.*<br><br>[Pause and allow time for silence]<br><br>*Now see if you can extend the stance you had toward your partner's pain to your own pain... See if you can connect to the stance that you are more than your pain, you are more than your moral injury, just as you hold them as more than their pain…as more than their moral injury. We aren't asking you to* | Ideally, the previous elements of the exercise will have prepared the clients to relate to their own moral pain in a more flexible, accepting manner. It is not unexpected however that clients may express hesitancy or dread at holding their own pain again. This is a natural human tendency to avoid pain. Clinician's can compassionately acknowledge this and encourage the client to continue with the exercise. |

| | |
|---|---|
| *condone what happened, or deny responsibility, or agree with it…but we are inviting you to make room for it...that it has undeniably impacted you...and that you still have a choice about how you carry that pain. See if you can extend your behavior toward your partner to yourself…choosing to hold your pain…your experience of a moral violation…with the same care, compassion and connection that you did when you held your partners pain [silence].*<br><br>*Observe the rise and fall of your pain again…and practice making the choice, over and over again to open your palm to it…physically opening your palm to your pain…and opening up to it internally…welcoming the significance of the experience…as an experience you carry…rather than an experience that you are. As you hold your pain lightly, what are you free to do?* | This part of the exercise leverages the clients' tendency to recognize the humanity of their group members and feel pleasant moral emotions towards them. It invites them to in turn grant the same humanity and dignity to themselves, as they did previously to their partner.<br><br>Pivoting to connect a stance of openness and flexibility to the client's stated values |
| **Processing** | |
| *…Now staying in touch with the experience of holding your own pain again in this new way, we'd ask you to open your eyes.*<br><br>*What did you notice this time as you held your moral pain? What was different?*<br><br>*What are you free to do when you hold your pain like this with the group members in this room? (as opposed to when you're consumed with trying to make it smaller)—you could listen to their experiences…connect with their pain…be present for them and show up…while still having your pain.*<br><br>*What would you be free to do in your life if you held your moral pain in this way?*<br><br>*Did you notice any different experiences when you held your own moral pain differently?*<br><br>*Did you notice any urges about what to do with your partner's moral pain? How did you respond to holding their pain?*<br><br>*How did you know when you were dropping the rope? What does that mean for how you hold…how you carry your moral pain with you?*<br><br>*How might you practice holding your pain going forward? How will you carry it?* | If clients don't express self-as-context, you may ask, "*Who holds the pain?"*<br><br>Amplify when clients identify these possibilities *(e.g., When we talk about living values this is what we're talking about!)*<br><br>*Holding pain with compassion, acceptance for others vs. holding pain with openness for self.*<br><br>If stuck*: What does it do for you to hold your pain in this way…tight…what happens as you squeeze?* Let's follow this out a bit…what happened right after that…right before it? |

**Clinician Note:** This exercise, like others that involve directly contacting and working with moral pain, often involve opportunities to deepen experiential contact with uncomfortable emotions. Clinicians can stay attuned to these opportunities and take advantage of them where possible. For example, if a client says of their paper, "*I am worried it will be found and read*," the clinician may respond, "*It sounds like it's not just guilt, but also fear… or shame… What has holding onto this secret cost you?... Would you be willing to hold all these experiences and still move toward your* [values, family, purpose, etc.]*?*"

**Bridge to Skills Practice**
*We would like to continue to practice holding your moral pain with openness, with freedom to move…while living your values over the next week. Can people identify ways they might do this? Think about how you might connect with what matters in your life while you do this. As you picture yourself…living this value…acting in this way that matters to you. What pain might come up…maybe it's showing up for you now? For a moment we would like each of you to imagine yourself taking this action while slowing down and making room for those thoughts and emotions, just as we did today.*

**Skills Practice**
*Until we meet again, we want you to continue taking steps to live your values in your day-to-day life. As part of these steps, we would invite you to practice holding your moral pain just like we practiced in today's session. Try to notice when you're feeling moral pain and bring your awareness to how you're holding it. Then see if you can hold your own pain with the same respect and care that you held your partner's pain today. Also notice if you're feeling urges to avoid or fight the pain. If these or other urges come up, try to be a neutral observer of them rather than reacting to or avoiding them. Also, if there's an opportunity this week to hold someone else's pain or allow them to hold yours, consider using the skills we've practiced today to just sit with and hold that pain together with them.*

**Timeline Exercise**
*We also want to look ahead to some of the work we'll be doing in the latter half of the group. So far in ACT-MI we've been learning about new skills to help us hold moral pain and do things that matter in our lives. In the latter half of the group, we'll to build on those skills by learning more about how they apply to your morally injurious experiences as well as other parts of your lives.*

*To help prepare you for that, we'd like to give you an exercise to complete before our next group session. In your ACT-MI workbook for this session you'll find a worksheet called the Timeline Exercise. On this sheet is a line that represents the time you've been alive up until this point. It starts on the left with a circle that represents when you were born and continues to a circle on the right that represents this moment right now. The space above the line represents experiences that have been morally pleasant. This includes times in your life so far where you've felt inspired, grateful, proud, or caring. The space below the line represents experiences in your life that have been morally painful… times when you felt shame, guilt, embarrassment, anger, disgust, or contempt. The distance from the line represents how intense the experience was (e.g., closer to the line means the experience was less intense, farther away indicates it was more intense).*

*Before our next group session, we'd ask you to place dots on the page to mark the different events in your life that were either morally painful or morally pleasant. These could be events from before, during, or after your military service. When listing your morally painful experiences, include your morally*

*injurious event, as well as other morally painful experiences that have been significant in your life thus far. Morally pleasant experiences could include times when*

*… someone did something that changed the course of your life for the better,*
*… you were able to help someone else who needed you,*
*… you achieved something meaningful that had felt hard or out of reach,*
*… you witnessed something that made you want to live your values more fully.*

*Place the dot either close to or farther away from the line to indicate how intense it was at the time (farther above or farther below the line means more intense). Label each dot and write the approximate age you were when that event happened. There is no right amount or kinds of events to include but try to capture those that have seemed most impactful in shaping how you've lived up to this point in your life.*

*After you've listed the morally painful and pleasant experiences in your life by drawing dots and labeling them on the page, go back and look across the entire page. As you're reviewing the timeline you've created, try to answer the following questions:*

- *Right after each of these different events happened, how did you respond? How are you still responding to them now in the present?*
- *At the time of the event or shortly after, what messages or conclusions did you make that you're still holding onto? How has holding onto those messages or conclusions affected your ability to live your life?*
- *Notice if there are any patterns in the dots. Do they tend to fall on the left or the right? Above or below the line?*
- *Notice the space between the dots. What do the dots you've made leave out or ignore?*

**Post-Session Clinician Review**

- Check-in internally with experience of session and reflect on personal or clients shifts that were noticed.
- Identify potential patterns of behavior observed to be aware of in future sessions.
- Compassionately identify any self-care needs that became evident during the session and, if willing, make plan to address them as soon as workably possible.

# SESSION 7

## CASE CONCEPTUALIZATION AND FUNCTIONAL ANALYSIS

**Purpose of Session**
The purpose of this session is to complete a second individual chain analysis for each member of the group. This second chain analysis both reviews and builds upon the case conceptualization developed after in session 1 and throughout the subsequent group sessions. In this session, the clinician uses the second chain analysis to refine the client's individual case conceptualization and review with the client the short and long-term impacts of their morally injurious behaviors. This session also adds the step of identifying instances where ACT-MI skills can be used to interrupt patterns of moral injury and facilitate healthier alternative patterns of behavior that instantiate meaning and purpose in the client's life.

As with the chain analysis in session 1, the content of these individual case conceptualization sessions can be used to help focus and tailor discussions on the factors most salient to each group member's moral injuries in subsequent group session.

**Materials Needed**
- ACT-MI Chain Analysis Worksheets

**Pre-Session Clinician Preparation**
- 1-minute mindful check-in with self (noticing own contact with the present vs. conceptualized pasts and futures, potentially including those about group members)

**SESSION CONTENT**

**Review case conceptualization/functional analysis**
*This meeting's purpose is similar to the first individual meeting you attended at the start of beginning ACT-MI. Like that session, we'll go through an example of a recent time that moral injury showed up in your life and better understand what triggered your behavior and what is keeping that pattern alive. We'll also spend some time exploring ways we can break that pattern by using the skills we've learned so far in group.*

| Example Clinician Language | Notes for Clinician |
|---|---|
| **Clinician Note:** Although chain analysis can be a clarifying and valuable tool for helping clients understand their behaviors, it is important to also track the client's emotional experience of the exercise. For example, some clients may find chain analysis to be a validating experience in that it helps them see that their suffering stems from understandable, if unhelpful, motivations to escape pain. Yet other clients may experience the chain analysis as emotionally evocative as they contact emotions and memories they otherwise attempting to avoid. | |
| **Identify target behavior** | |
| *Tell me about a recent behavior of yours that you'd like to understand better. For example, it could be behavior that you'd like to change so you can better do what matters to you.* | |
| **Identify Triggering Event and Vulnerability Factors** | |

| | |
|---|---|
| *What kicked this chain of events off for you…what triggered xx behavior?*<br><br>*Did anything make it more likely you would respond to [prompting event] in the way you did? (e.g., prior alcohol use, poor night's sleep)* | |
| **Fill in steps between target behavior and prompting event** | |
| *How did you get from here* (prompting event) *to here* (target behavior)?<br><br>[For each link in the chain] *What happened next after* [ _ ]*?* | |
| **Clinician Note:** The chain/functional analysis is meant to tell a story of the reinforcer(s) maintaining the Veteran's moral injury. If up the exercise starts to feel confusing or scattered, bring yourself back to the purpose of the exercise—to learn which behaviors are providing them relief or reward, and in so doing keeping their moral injury alive. The other purpose is to understand what the costs of these behaviors have been for the client. | |
| **Introduce Short-Term Consequences** (Why we keep doing what we do) | |
| *As we've shared before, people keep doing these behaviors because of the effect it has on us or our surroundings. If a behavior reduces something we don't like to feel, we keep doing it. If we are anxious and feel relief by isolating, for instance. If it increases our contact with something that feels good, we keep doing it. If you feel anger and you drink, and you feel more lose, for instance. It makes sense. Feeling better drives a lot of behavior. It's important that we know how this behavior impacts you and your environment—including other people around you—in the long run, so that we can identify strategies and tools you can use to give you more choice about what you do in response to moral pain.* | Morally injurious behaviors, by definition, lead clients to depart from values or cause themselves or others added suffering.<br><br>Providing education about how short-term consequences give immediate reinforcement can help give clarity about to why the client has engaged in these behaviors that otherwise appear costly and destructive. |
| **Internal Short-Term Consequences** | |
| *What did you notice immediately happened to your experience during or after* [target behavior]*?*<br><br>*What happened to your emotions…Your thoughts…Your sensations…Your urges…your memories during and/or immediately after engaging in* [target behavior]*?*<br><br>*Did anything shift about the intensity of these experiences?*<br><br>*What did you notice about how this behavior affected you in the moment?* | |

| | |
|---|---|
| *Did this behavior take away or reduce anything you didn't want to feel? (Did it give <u>relief</u> from something painful? Turn down the volume on the pain?)*<br><br>*Did it increase your ability to feel something you would rather feel instead? (Did it lead to something enjoyable?)* | Examples: avoiding guilt and shame, reduced anger, distracted from painful memory<br><br>Examples: sense power/control, connection to people who have passed away, vindication |
| **Long-term Consequences** | |
| *How does* [target behavior] *affect your relationships, spirituality, mental health, physical health, work?*<br><br>*What are the long-term impacts of* [target behavior] *on you? …On those you care about?* | Probe how the target behavior affects a range of functional domains (social relationships, employment, physical health, finances, religion/spirituality) |
| **Breaking Pattern of Moral Injury** | |
| *Now let's look over these links in the chain to see if we can find places where some of the skills and tools we've been learning might have been helpful. What do you notice?*<br><br>*Where might it have been helpful to slow down?*<br><br>*Was there a thought you might have gotten hooked on or some pain you were pushing away?*<br><br>*How could you have gotten in touch with your Observer-Self or with your values?*<br><br>*What physical action could you have taken that might have demonstrated what you care about?* | Guide the client in identifying where ACT-MI skills might have helped interrupt morally injurious patterns of behavior. As needed, make suggestions to the client,<br><br>Even small actions, particularly early on in the chain's sequence of events, can help clients change the outcome of the situation. |
| **End Chain Analysis** | |
| *Thanks for working with me to better understand how these patterns of moral injury show up in your life. I want to check in with you and see what you've take away from this session.*<br><br>*What stands out to you?* | Clinician listens, validates, and responds supportively |

**Closing of session**

- Check for understanding about the assignment and invite any further questions from the group members and discuss any issues that are brought up.

*In this session we've begun identifying some of the patterns of behavior that are keeping moral injury alive for you. We'll have the opportunity to individually check-in again in the future to update and explore this more. In the meantime, I want you to take what we've started here and notice when these patterns are happening in your day-to-day life.*

*When you come to group next week, we'll be helping you learn some techniques and tools for breaking these patterns so you can be free to live your life with more meaning and purpose.*

**Post-Session Clinician Review**

- Check-in internally with experience of session and reflect on personal or clients shifts that were noticed.
- Identify potential patterns of behavior observed to be aware of in future sessions.
- Compassionately identify any self-care needs that became evident during the session and, if willing, make plan to address them as soon as workably possible.

**Chain Analysis Common Challenges and Possible Clinician Responses**

| Target Behavior Challenges | Possible Clinician Response |
|---|---|
| Client gives a broad, generalized target behavior (e.g., drinking) | Elicit further specific details (e.g., Tell me a time when you were drinking. Where were you? How much?) |
| Client identifies another person's behavior | Validate the event and invite them to share what they did in the situation that might be keeping them stuck. |
| Client gives a covert behavior (mental actions) | Clinician may need to provide more labeling (i.e., labeling the behavior 'rumination') |
| Client gives an example of NOT doing a valued behavior | Help the client identify what they were doing instead of the valued behavior |
| **Prompting Event Challenges** | **Possible Clinician Response** |
| Client has trouble identifying what the prompting event is because it happened so quickly | Work backwards from target behavior to get the full episode of the behavior ("*What happened right before*…") |
| Client has trouble identifying what the prompting event is because it feels so routine | Explore if there was something distinct about the timeframe/circumstances of this particular episode (Tell me what was unusual about this particular day)<br><br>Explore events/situations that may serve as vulnerability factors (e.g., anniversaries, alcohol use) |
| **Link Challenges** | **Possible Clinician Response** |
| Clinician or client feels unclear about the timeframe of the chain | Identify the time span between the prompting event and target behavior happened (e.g., two hours) |
| Chain feels unclear or doesn't tell the story of the behavior | Be direct and curious with the client about this and invite the client to collaborate with you in helping to understand potential missing links |
| Client experiences shame and self-critical thoughts in response to chain analysis | Use frequent validation techniques such as, validating their experience, normalize the experience as a product of their prior experiences. Eventually validate by recognizing short-term consequences that reinforce the behavior. |
| Clinician experiences discomfort in reaction to client's moral pain | Use ACT-consistent skills to lean into and validate both the client's and clinicians own experience |
| **Short-term Consequence Challenges** | **Possible Clinician Response** |
| Client is focused on long-term consequences of the target behavior and so can't validate that their own experience makes sense. | Reorient the client to the immediate consequences of the target behavior (e.g., during the target behavior or seconds or minutes after). "*What happens in that moment as you are* [insert target behavior]?" |
| Client doesn't report their target behavior reduces painful emotions and so can't be reinforcing in the short-term. | Explore whether the target behavior is ***positively*** reinforcing, such as increasing a sense of control or by creating a more preferred emotion (e.g., feeling guilt rather than shame). |
| **Long-term Consequence Challenges** | **Possible Clinician Response** |
| Client doesn't report their target behavior has long-term negative consequences for them. | With curiosity and openness, inquire what the costs of this behavior have been in the past. If the client cannot identify any costs, explore further what has brought them into treatment and see if any long-term consequences of |

| | |
|---|---|
| | morally injurious behaviors can be identified. If none can be identified, consider checking with the client's referral source and clarifying purpose of ACT-MI as needed. |

# SESSION 8

# HOLDING MEMORIES AS AN OBSERVER

## CLINICIAN ORIENTATION AND PREPARATION

### Purpose of Session

This session's purpose is to expand upon the concept of the observer self that was introduced in a previous session. In this session, clients are guided to understand that the observer self exists not only in the present moment but has existed in all their lived moments in the past. In widening the application of the observer self to the past as well as the present, clients are guided in recognizing that in addition to not being defined by their present internal experiences, they are also not defined by their past experiences. Furthermore, by considering the variety of experiences and events they have experienced thus far in life, clients are brought into direct contact with the limiting nature of their stories about themselves, particularly those related to their moral injury and morally injurious events.

### Materials needed

- ACT-MI Workbook

### Pre-Session Clinician Preparation

- 1-minute mindful check-in with self (noticing own contact with the present vs. conceptualized pasts and futures, potentially including those about group members)

## SESSION CONTENT

### Bridge from Previous Sessions

*We would like to spend some time interacting with the timeline exercise you did as a practice exercise. First, we'd like to check to see many people did the practice exercise.* [Clinicians provide additional instructions as needed to help clients recreate the timeline as needed such as "*If anyone didn't do the exercise, please take a moment to get a piece of paper and a pen and quickly recreate the timeline by drawing a horizontal line through the middle of the paper with a circle at each end.*]

### Introduce New Content

*As a reminder, the purpose of this exercise was to create a landscape of some of the key experiences you've encountered across your lifetime with morally pleasant events marked as dots above the line, and morally painful experiences marked as dots below the line. We're doing this so we can practice holding these experiences as events you have lived… as an observer who can hold these memories… just like you held the pain ball… and because you can hold these memories means you are bigger than them. This will shape some of our work together over the next few weeks. We'll ask you to notice things about your timeline and then discuss what you noticed as a group.*

### Experiential Exercise

- Conduct experiential and process elements
- Respond to challenges in comprehension or application of exercise
- Generalize skills to outside of session

| **Example Clinician Language** | **Notes for Clinician** |
|---|---|
| **Experiential** | |
| *First, we would like you to look at the timeline… Notice significant events in your life thus far and observe where they fall on the page… Notice how far above or below the line you marked them... Notice what periods of your life they occurred in…* | Give enough time for clients to emotionally connect with their timelines. |
| **Processing** | |
| *We're interested in hearing about what you noticed. Did you list more events earlier in your life or more recently?...*<br><br>*Did you list more painful events or more pleasant events?...*<br><br>*Where there any significant gaps in your timeline where you didn't list any events?...* | Observe the degree to which clients appear invested or tied-up in the stories they have about their pasts and how those stories impact their sense of freedom in this present moment. This will be an explicit focus in later parts of this exercise. |
| **Experiential** | |
| *Spend some time connecting with a painful event… making room for it and observing the emotions… thoughts… urges… sensations it evokes. Noticing the sense of weight that accompanies this event… slow down and allow the experience in… noticing that this event is one event you have lived across the course of your lifetime… This one event occurred at a very specific moment in time, is an incredibly significant event, and yet still is just one event that you have encountered across your lifetime. Notice that you are the one that has had this painful experience… but because you are outside of the event noticing it, that means that it doesn't completely define you. You experienced the event when it happened back then, and you here now are observing the memory of it. Like the paper that holds the writing, you are an experiencer… the holder of this memory.* | |
| **Processing** | |
| *How have you held this event in the past? What was the impact of responding to the pain with* [substance use, aggression]*?)*<br><br>*How would you hold the painful memory of [Client]? Can you practice holding your own painful memory, in the same way you would hold theirs?*<br><br>[If client's attempt to justify or explain any of their past or present behavior, re-visit the observer self with questions like…]<br>- *Is that another piece showing up on the board?* | *Revisit creative hopelessness*<br><br>Draw on previous sessions involving holding one another's pain.<br><br>Revisit and apply the observer self to moral pain |

| | |
|---|---|
| - *[Client], who's aware of having that piece?*<br><br>*Can you put down [your humor/words] and just be with this in this moment?*<br><br>*Can you practice just holding that experience in this moment?* | Invite and practice acceptance of moral pain |
| **Experiential** | |
| *Now spending some time connecting to a pleasant event… opening up to it… observing the emotions… thoughts… sensations… urges it evokes. Allowing these experiences… whatever shows up... to come and go… observing them… holding them. Noticing the significance of this pleasant event. It too happened at a specific place and time… Taking a moment to notice that no matter how profound this pleasant event was… that it too is just one experience on the infinitely expanding page that is the observer-you. That you hold this event in your awareness in the same manner as the painful event, and therefore you aren't be defined by either of them….* | |
| **Processing** | |
| *What did you notice while holding the pleasant experience?*<br><br>*Who's noticing the pleasantness?*<br>*Even though it may be pleasant, it's still not all of you.*<br><br>*Can we take a moment to just acknowledge and sit with the pleasant memory, and anything else that may come with it?* | Practice similar skills of seeing from the observer self and acceptance<br><br>Because of the values-pain connection, it is possible that eliciting pleasant moral emotions will also elicit painful emotions. Make room for all of these. |
| **Experiential** | |
| *Now shifting from the events that you've listed…spending some time to notice the spaces between the events on the page where nothing is written. Did life stop happening in these gaps? Slow down and see if you can notice what arises. Notice again that you were living and experiencing during these times too, even if they are not as memorable. And the You that is here now was still there then during these times as well, holding the experiences of these encounters too.* | By directing attention to how little our stories contain about our total prior experience, we undermine the power of these stories to limit future possibilities. |
| **Processing** | |
| Did life stop happening during the gaps? What might it mean that life didn't stop happening in between the significant events you remembered?<br><br>What might it mean that you can't remember? | We draw attention to the continuous existence of the observer self, who can actively choose new directions<br><br>Clients may return to stories and explain why change isn't possible or |

| | |
|---|---|
| If you had to estimate, what percentage of moments in your life can you remember? What might that mean about how we try to define ourselves by our memories?<br><br>Were there things happening in these gaps that still mattered? Like what? | why they are defined by their past. If this happens, apply the observer self to these stories/explanations (e.g., *Who's having that thought right now?*) |
| **Experiential** | |
| *Now take this physical piece of paper that represents the timeline of your life so far in your hands… holding it like the chessboard. The page can hold all the events… memories… experiences… without becoming or being defined by any of them. Like the page holds the writing, you are the person that holds these memories…that has experienced each of these events… yet isn't fully defined by any of them. You went through a morally injurious event… and you also entered relationships… maybe had a child or [reflect on other notable experiences that are positive events on timeline]. There's a You here now that has experienced all these events yet isn't any single event in isolation. This You has always existed and always will exist… and has been present for every moment you have encountered. As time keeps moving and you keep moving… there's a You that will always hold your experiences… no matter how pleasant or painful they are.* | Emphasize primary message that observer self is larger than any set of events or experiences and has been available throughout client's lifetime, including the present moment. Furthermore, the observer self is available for clients to connect with, even when it is not recognized. |
| **Processing** | |
| *We'd invite you now to stay in touch with this observer you that has always existed and is able to hold all your memories and share with us.*<br><br>*What would it look like to attend, not only to the big pieces on the page, but also all the little pieces that are happening day in and day out?*<br><br>*Where could you practice showing up to these small moments? (ex., when you get home from work, when you pick up your child)*<br><br>*What might you do to show up more to these moments?* | While processing with clients, continue to attend to indications that they are maintaining contact with their observer self (a sense of freedom and openness, unconstrained by the past).<br><br>Explore how the observer self might impact their ability to live their values.<br><br>Emphasize small, repeatable actions over large, resource-intensive changes. |

**Bridge to Skills Practice**

*Thank you for sharing with us. We've emphasized today that each of us are more than any one event or experience. Today we stood back and looked over our whole lives up until this point. Over the next several weeks, we'll be diving deeper into some of these events as a way to practice these skills.*

**Skills Practice**

*As we did last time we met, we want you to continue taking steps to live your values in your day-to-day life. You may want to notice where there are "gaps" like the ones we noticed today where it doesn't seem like anything particularly significant or meaningful is happening. We'd like to suggest that every moment can be meaningful if we choose to approach it with a purpose. We encourage you to step into these gaps in your coming days and make a conscious decision to do something… even if it's small… even if it doesn't dramatically change the situation… but do something anyway that tries to moves you in the direction of what you care about.*

*As you practice living your values moment by moment, practice awareness that your observer self is there watching and holding all your experiences, including both the pleasant and painful, and that it's this observer self that gives you the freedom to choose how you'll live your life.*

**Post-Session Clinician Review**

- Check-in internally with experience of session and reflect on personal or clients shifts that were noticed.
- Identify potential patterns of behavior observed to be aware of in future sessions.
- Compassionately identify any self-care needs that became evident during the session and, if willing, make plan to address them as soon as workably possible.

# SESSION 9

## OBSERVING PAINFUL MEMORIES

## CLINICIAN ORIENTATION AND PREPARATION

### Purpose of Session
This session's purpose is to practice connecting with the continuous "observer you" in relation to painful moral events in the past. The clinician is working to assist clients in connecting to a felt sense of self that is the context for their experiences. Self-as-context should be the main orienting process with interventions during the session linking to this overall goal. The function of this work is to help clients contact self as experiencer, rather than the experienced, thereby lessening the sense of threat from any painful emotion, memory, or thought. There is freedom in this perspective as it supports choice guided by values. The client, able to recognize self as larger than any moral injury, can choose to pursue their values rather than engage in emotion-driven avoidance-based actions.

### Materials Needed
- ACT-MI Workbook

### Pre-Session Clinician Preparation
- 1-minute mindful check-in with self (noticing own contact with the present vs. conceptualized pasts and futures, potentially including those about group members)

## SESSION CONTENT

### Bridge from Previous Sessions
*Last session we spent some time exploring how the observer you has been there across your entire life including pleasant and painful events, and even during the moments you can't remember. We're going to continue that work today by inviting you to engage in an exercise that picks up where we left off last week.*

*If you recall, last week we explored how you've had many experiences, both morally pleasant and painful across your lifetime while also noticing that there is an observer, or noticer of these experiences. We contacted a sense of you that is more than any individual experience, positive or negative. You are the "space" – the chessboard, the piece of paper, that holds these experiences, you are not the experiences themselves. In this next exercise, we will continue to explore this sense of you, growing your contact with it.*

<table>
<tr><th>Example Clinician Language</th><th>Notes for Clinician</th></tr>
<tr><td colspan="2">Clinician Note: This is a more extended practice exercise designed to further reinforce the distinction between the self as an experiencer (i.e., the observer self) and the thoughts, emotions, sensations and urges that are experienced. The purpose of this extended practice is to help prepare the clients to contact and hold the pain associated with the memory of the morally injurious event later on in the session.</td></tr>
<tr><td colspan="2">Experiential</td></tr>
</table>

| | |
|---|---|
| *So, to practice connecting with the you that can notice experiences, the observer you, I'll have you start by getting settled in your chair. You can leave your eyes open or closed. There will be a point during this exercise where we will invite you to look around the room. And so when we do that, just feel free to open your eyes if you choose to close them. The other thing that's important to recognize about this exercise is that when we finish the process of coming into contact with the flow of experience, I'm going to ask you a set of questions about those experiences. Those questions are going to repeat themselves, but hang in there with me, we're headed somewhere with the exercise. Also, when I ask the questions, you can simply answer them in your head to yourself, we will explore your experience when we finish the exercise.*<br><br>*First, take a couple of breaths and place your feet squarely on the floor. Now I'm going to invite you to shift your attention gently to your feet. Just become aware of the sensations that you experience of your feet on the floor... What do you notice at your feet? Perhaps you notice tension, pressure, maybe even pain, just rest your attention on the experience at your feet…*<br><br>*Now gently releasing your attention from what you notice at your feet. I'm going to invite you to move your attention, your awareness to sound… Noticing sound, begin to be aware of all the sounds that you can hear in the room. There may be louder sounds or more subtle sounds. Maybe close sounds or distant sounds, spend some time letting yourself wander around sound for a moment, noticing the different sounds that you're aware. And now I'm going to invite you to tune into a single sound. Pick one sound that you're listening to and rest your attention on noticing that sound and label it. Name the sound in your head…*<br><br>*Now releasing your attention from sound I'll invite you to open your eyes and just gently look around the room and just notice all of the different objects, colors, textures, and shapes, as you look around the room… Simply observing, focusing fully on the experience of seeing... Then find one object in the room and rest your eyes on that object. Looking at one object. Labeling or naming that object in your mind… And then releasing your attention from looking at this object we'll invite you to return your attention to the group and questions I'm about to ask about your experience. You can just answer these questions to yourself, there's no need to speak yet. These questions repeat themselves so be prepared for the same questions. I know that* | You may encounter times when clients will give responses like, "I am those experiences" or "They are me," or even, "I did become my feet," somehow indicating that experiences are "theirs" and therefore are them. If this happens consider continuing the exercise out loud, engaging in a brief dialogue or set of questions that might help the client to contact the observer self. For instance, you might ask, *"Who knows (or sees) that was you hearing, seeing, and sensing?*" and perhaps follow it with "*This is about the 'knower'.*"<br><br>You can also reflect back on the Timeline Exercise from the previous session, reminding them that they are the space where these experiences occur.<br><br>You can also note that the experiences are embodied, they don't exist outside of the person. Even then, the experiences rise and fall, come and go, and there is an observer self that remains aware of, and distinct from these changes. Contacting that sense of self-as-observer (the self who is aware of their experiences) is the focus of this exercise.<br><br>You might also have clients who misinterpret the exercise and get lost in terms of is purpose. For instance, a client engaged in the exercise once reported that he noticed the carpet, plant, and temperature of the room while focusing on his feet. In a case like this, you can, instead of commenting on losing the exercise, simply ask, "Did you become the |

| | |
|---|---|
| *you're going to be answering way ahead of me once we get started, but no worries, just stay with us.*<br><br>*I started by asking you to pay attention to the sensations at your feet, and when you did that, did you become your feet? Did you become the sensations you noticed there? Or was there a You there noticing the experiences you were having at your feet, a You that is more than the experiences at your feet? A You that was observing those experiences?*<br><br>*Then I asked you to pay attention to sounds, and you noticed the sounds in the room, and then you were invited to notice one sound and you labeled it. Recall that sound you labeled. Did you become sound? Did you become that sound because you heard it? Or was there a You there hearing, noticing that sound, experiencing that sound? A You that was more than the sound. Also, notice if it was the same You who was observing or noticing the experiences you had at your feet. This sense of You was there for both experiences, feet, and sound. A You that stretched across those two different places and was there for both.*<br><br>*Hang in there with me and now let's go to what you saw and recall the object you selected and labeled- the one item. Did you become seeing? Did you become that object because you saw it? Or was there a You there, seeing, an observer experiencing what you saw? This observer you was there for what you saw, what you heard, and felt at your feet. The same You were there for each experience. This part of You stretches across time and was there for each experience and was not those experiences themselves.*<br><br>*And if it is the case, that you didn't become your feet, you didn't become what you heard, and you didn't become what you saw, could it also be the case that you also don't become your thoughts, feelings, or memories? That these are also experiences that you have. Just like you have experiences with your feet, ears, and eyes, also you have experiences called 'thoughts', that You can notice. You have experiences called emotions and you can notice those emotions. You have memories that you can recall, yet you are not those memories either. You are <u>more</u> than these. And you are not your moral pain. There is a You, here, now that is the experiencer of all these events, and that You is more than them. You are a place that these experiences flow through… you contact them… you sense them through your body… but there is a constant and steady sense of you, that has always been and always will be.* | carpet, plant, or cold because you noticed it?" thereby, bringing the client back to the work at hand. |

| **Processing** | |
|---|---|
| *What did you discover in answering the questions from the exercise?*<br><br>*Who's the one noticing that experience?*<br><br>*Do you notice any ropes you're holding onto right now? Would you be willing to drop them, allowing the pain to just be here in this moment for now?* | Practice self-as-context and acceptance with clients responses with the goal of connecting them up to these skills prior to the next exercise where they will connect to the memory of the morally injurious event |

**Brief Check-in on Skills Practice**
Due to the focus on visiting the memory of the morally injurious event this session, review of practice exercises is kept to a minimum. Clinician's may consider asking a few groups members questions similar to the following:

- *How have you been practicing living your values?*
- *What actions did you take?*
- *If you didn't take action this week, what was the barrier to doing it?*

**Introduce New Content**
*We'd now like to take a closer look at and continue to grow how we can understand this larger sense of you and your moral injury. You may already be getting a sense or understanding of what we are trying to do and continuing this process will only help to solidify it. The work here may be challenging, but it is also incredibly important. It is one of the main ways that you can find some freedom, letting go of the rope, and moving in a values-based direction. We will largely do this work through an experiential exercise followed by exploration of what it means."*

**Experiential Exercise**

- Conduct experiential and process elements
- Respond to challenges in comprehension or application of exercise
- Generalize skills to outside of session

**Introduction**
*Okay, let's continue practicing connecting with the observer you, the You that can notice your experiences without them defining you or becoming your fate. Your experiences are not the whole of you, just like your moral injury is not the whole of you either. There's an observer you who can hold all of your experiences without becoming any single experience.*

*We are going to practice connecting with the memory of you experiencing a moral code violation while the You, here, now, who is experiencing that memory, can observe it without becoming captured by it. Again, we know this will be challenging, but remember, there is a You that has the memory instead of being the memory. If there's a You that can observe your memories, a You that has always existed, then there is a You that is more than any memory and more than your moral violation.*

| **Example Clinician Language** | **Notes for Clinician** |
|---|---|
| **Experiential** | |
| *I'll invite you now to close your eyes and take a moment to settle into your chair and sense of your body. If you notice yourself bracing against or withdrawing in any way, see if you can soften as we begin this work… You are now invited to connect with the you that experienced a significant moral violation in war or elsewhere, take your time, you can approach it slowly bring the memory to mind… As you contact the memory, begin to notice what you're doing… Notice other aspects as well. What are you wearing? Are you with others? Spend some time with this memory… Notice any urges to disengage from it, to push it down, to run. Let those urges be there, simply noticing them as they rise and fall… Alongside those urges, you are invited to notice whatever emotions are also present. Gently observe these emotions, even if they are powerful, and continue to stay present simply observing your experience of emotion. Perhaps you can observe if these emotions seem to be located in a certain part of your body, notice where they are located… Take a moment to make room for the possibility that your pain signals important values for you, you feel pain because you care.*<br><br>*As you continue to observe the memory and the urges and emotions that come with it, see if you can notice that this moral violation, this experience, in this moment, is a memory. It's something that happened in the past, and you are here now observing it… It happened at one point in time and had a big impact, but here in this moment, it is not happening, you are noticing the memory of it… Notice a kind of continuous you that knows that it was you there then and knows that this is you here now, observing the memory - a continuous you that was there then and is here now.*<br><br>*Finally, let's notice what your thoughts tell you about this memory. What do your thoughts say about your role in the situation? What does your mind tell you about other peoples' roles?… Notice if your mind is judging, or criticizing, notice if they are calling inhuman or evil or that you don't deserve the life you have. Maybe the thoughts have been about penance whatever the thoughts, observe these thoughts as they rise and fall, come and go… Notice if you're having the urge to jump in a fix these thoughts, to argue with them or to make them go away… Notice that you have thoughts, that you can observe thinking… As you continue to notice thinking also notice that there is a You here now that is aware of these thoughts about your memory. This sense of you, the noticer, is more than your mind and memories including the memory of* | As the client(s) will be experientially "facing" the morally injurious event during this session, it will be important for the clinician to be fully present and to consider pacing. Remember to slow down, especially in moments where emotions might become intense. It will be important to refrain from rushing the session or becoming too explanatory or overly didactic. Contacting the sense of self is not a verbal process. Explaining too much or overly worded descriptions will miss the point of the experiential exercise.<br><br>As you will be inviting the client(s) to contact the memory of a moral injury, remember to be patient and respectful. It will not be unusual for the client(s) to want to resist or avoid the exercise. Gentle encouragement might be needed. You may need to remind them that reducing avoidance is part of the healing process, and although difficult is a vital part of recovery.<br><br>There may be moments during this session where slowing down and inviting openness are needed. You may need to prompt the client to remain present or to notice the urge to "run" or the urge to fix or escape. Invite them to ride the wave of the urge and to defuse from mind, noticing any thoughts but letting them pass while remaining engaged.<br><br>Given that this exercise is a longer one, you may want to make sure the client(s*)* are still following your prompts and engaged in the exercise. Consider asking them to raise a finger from time to time to |

| | |
|---|---|
| *your moral injury. Be aware of a larger sense of you, who holds these experiences, thoughts, urges, emotions, and memories, yet is none of them… Just as you were not your feet, or what you heard or saw in the earlier exercise, you are not your thoughts. You are not your memories You are not defined by any one experience. You are the experiencer…*<br><br>*Not as a matter of dismissing or discounting the morally injurious memory and its associated emotions, urges, and thoughts, but as a matter of freedom to begin living the life your morals tell you that you want to live. Imagine that you could hold your experiences and let yourself be guided by your values as a matter of choice in moving forward in your life.*<br><br>*You are now invited to notice the here and now by focusing in on your body sitting in the chair… attending to sound… and then gently open your eyes.* | indicate that they are still participating. |
| **Processing** | |
| *What did you notice while participating in the exercise? What are you noticing now?*<br><br>*What did your thoughts say about who you were and how you had to live after the moral injury?*<br><br>*How weighty did the thoughts/emotions/urges seem?*<br><br>*How much have these experiences seemed to push you around or define you in the past?*<br><br>*If you could observe the You that your mind says you have to be in response to your moral injury, rather than attaching to it, what possibilities might present themselves in your life?*<br><br>*What choices might you have if you were guided by your values instead of a critical thought? What might look different?*<br><br>*How could you show up in your relationships and in your life?* | One of the most difficult parts of self-as-context work is the client getting caught or lost in the content of the moral injury. This can happen with respect to the actual memory or in response to questions and discussion. For instance, a client might reflect and say that equating an experience of the foot is very different than the moral injury. Here you can reflect that the goal isn't to equate the two experiences, but rather to notice that both are experiences the client has had. Work to bring what is happening back to the goal of the exercise, by stepping back from the content being shared and instead focusing on process... You might invite them to notice if they can in this moment *"Who is having that thought right now? And who knows that you are thinking a thought? There's a part of you here that can notice that your mind telling you something. Can you notice that part of you right now?"* |
| **Clinician Note:** This exercise is not designed to diminish the moral injury in anyway or its powerful consequences. This encounter with the memory may be painful, but we are not asking clients to | |

experience pain for pain's sake. Our purpose is to help them see themselves as more than the event and more than the moral injury.

Other questions/responses that might be useful when clients get caught in content include:
- *Who's doing the remembering? Who's the knower?*
- Client: "*I don't deserve to be the observer,*" Clinician: "*Who just said that?*"
- *You're more than the commentator who is talking right now.*
- *We see that this are very heavy experiences. Hang in here with us… there's something very important for how you will carry this experience. We just want to bring in a part of you that can observe and choose.*

You can also reflect back to earlier exercises (e.g., you are not your feet, hearing, seeing and you are not your memories; chessboard; the Timeline). If the client says something like, "*Yeah, but this is different*," gently remind the client in a soft way, that even the thought "*Yeah, but this is different*," can be held and you can validate that it feels different and powerful, and yet, there is still a larger sense of self, here now, having that thought. All of these gentle challenges should be done in a way that validates both that the client is having real experiences but also softly pointing to the observer self that is the one experiencing them.

**Bridge to Skills Practice**
*The observer self is the same self that carries all of the events described in the session in the Timeline Exercise, is the same you that experienced and remembers a moral injury and is the same you that sits here today. There's a You here now that stretches across time and holds all of these memories yet isn't defined by them experience.*

**Skills Practice**
*As always, our invitation you this week is to use what you are learning here in session to more fully live your values in your day-to-day life. We believe that you'll be more free to do this if you can remind yourself that you are not your moral pain or any other thought, emotion, sensation or urge. Notice when you are hooked by your mind telling you who you are or who you need to be in response to the moral injury and notice that there's a You that can observe that thought and make a choice about how you will live with meaning and purpose in that moment.*

**Post-Session Clinician Review**
- Check-in internally with experience of session and reflect on personal or clients shifts that were noticed.
- Identify potential patterns of behavior observed to be aware of in future sessions.
- Compassionately identify any self-care needs that became evident during the session and, if willing, make plan to address them as soon as workably possible.

# SESSION 10

# OBSERVING PLEASANT MEMORIES

## CLINICIAN ORIENTATION AND PREPARATION

### Purpose of Session

This session's purpose is to create motivation for the living of values through contacting pleasant moral emotions. Just as with previous sessions where clients were guided to become aware of and taught how to hold their moral pain, this session brings these same skills to bear on memories, thoughts, emotions, sensations and urges that affirm clients' values and sources of purpose. As always, the inseparable connection between pain and values is honored, but clients are also given space to reconnect with cherished experiences and events that may have been forgotten or felt distant. These memories are then used to identify valued qualities that clients can choose to pursue in their own lives going forward. As was done with the morally injurious events, the memories of these sweet moments are viewed through the perspective of the observer self to reinforce that no internal experience, including the painful and pleasant, fully defines the client's existence.

### Materials Needed

- ACT-MI Workbook

### Pre-Session Clinician Preparation

- 1-minute mindful check-in with self (noticing own contact with the present vs. conceptualized pasts and futures, potentially including those about group members)

## SESSION CONTENT

### Bridge from Previous Sessions and Review Practice Exercise

*We've been spending the last several sessions exploring how our observer-selves extend across time. Last session we contacted the memories of the morally injurious events and practiced holding them in our awareness without being defined by them. We also talked about intentionally connecting with and finding meaning and purpose in the small moments.*

*What small moments were you able to show up for this past week?*

*What actions did you take? How did you connect with what matters to you?*

*If there were times you struggled, what got in the way? How did you get stuck or unstuck?*

### Introduce New Content

*As we explored in the timeline exercise a few sessions ago, we noticed that we all have both painful and pleasant experiences. Although we have spent a lot of time learning how to hold our painful experiences, it's also important to make space from our pleasant experiences as well. Today we'll practice exploring how you can hold pleasant memories and sensations too without becoming them…making room for all of your experiences…all of the pieces on the board… from throughout your life and for what they might signal matters to you. We'll do that today through another eyes-closed exercise.*

**Experiential Exercise**

- Conduct experiential and process elements
- Respond to challenges in comprehension or application of exercise
- Generalize skills to outside of session

**Introduction**

*This exercise has three parts: In the first part we're going to invite each of you to share a personal experience. We'll help you find the thing you're going to share in a moment.*

*In the second part, we'll ask you to open your eyes. When you open your eyes, please stay silent until I call on you. Simply listen while another group member is talking. When I call on you, please share the experience you brought to mind, but remember, we won't be responding to each other's experiences during this part of the exercise. Only listening. We'll ask you to be silent until we ask to hear from you individually in a later part of the exercise.*

*In the third part of the exercise, you'll have a chance to share more about your experience of the exercise. I'll be watching the time a bit because we want to hear from each of you. There will come a point after a few minutes when I'll give you a signal by holding up my hand and that will mean you have just about 30 seconds left. So, when I hold up my hand, begin to let the words you're sharing come to end and fade into silence again. If you don't finish your memory during that time… don't worry. We'll have more time to talk about it. I'll call on the next person to go after and so on. While you're hearing the story of each person, you're invited to listen deeply and respectfully while remaining silent and connecting with your experiences. Does anyone have any questions? Let's begin…*

| Example Clinician Language | Notes for Clinician |
|---|---|
| **Experiential** | |
| *For this first part of the exercise, close your eyes and take a moment to settle in… Now gently float back in time and flip through the card file…the folders… of your memories. As you look through your past, you're invited to connect to a sweet memory, a cherished memory. This could be from any time in your life but pick one that you're willing to share with the group. Take a few moments to find this sweet memory…* | Don't rush this initial portion of the exercise. Allowing the clients time to connect with and soak in this memory will help create greater connection and content to process later on. |
| *When you have landed on this sweet spot in your life, hold up your hand just briefly so that we can see that you have found one.* | Wait for clients to indicate they have found a memory |
| *Now you're invited to let yourself explore this memory…notice the sights… sounds… colors… textures… notice every part of this sweet experience… wander around the emotions… sensations… and thoughts that it evokes. Let yourself fully experience this cherished moment in time.* [Pause for a minute or two.] | |

| **Processing** | |
|---|---|
| *Now you're invited to open your eyes and connect to the room and people in it as we start the sharing of this memory. For this second part of the exercise, remember to listen intently as you hear other's sweet memories and remember that for now, we'll hold off on responding to each other's memories.*<br><br>*We'd like to ask [Name] to share what their sweet or cherished memory was. If you're listening and not sharing yet, notice what comes up for you as you hear [Name's] memory. Observe any emotions, sensations, thoughts, memories that arise for yourself, but remain silent until it's your turn to share your memory.* | Name each client and allow them to share for 1-2 minutes each. Thank each client after they have shared. |
| **Experiential** | |
| *Now you're invited to close your eyes once again… Take a moment to reflect on what you heard and what you shared... reflect on the experience… noticing the qualities of these sweet, cherished memories and what you felt while listening… their essence… Notice that each memory you heard holds meaning for the group member who shared it.*<br><br>*Now you're invited to return to your personal sweet memory, connecting to it again as you did at the beginning of the exercise. Notice it fully. As you come back to your memory, you're invited to observe what makes it sweet for you. Why is it cherished? See if there's something meaningful going on inside of this memory… something important… what makes it sweet… what values does the memory represent?*<br><br>*You're now invited to open your eyes and rejoin the room.* | |
| **Processing** | |
| *Now first we'd like each of you to share your experience of connecting to your own sweet memory.* [Invite first group member to share]<br><br>*What emotions did you notice as you brought this memory to mind?*<br><br>*What is important about this memory to you? What does it signify you care about?*<br><br>*We'd invite other group members to share what [Name's] memory evoked for them as well.* | Repeat this sequence for all group members.<br><br>Be mindful of how the group members may be reacting to each other's memories including envy or comparisons. If needed, bring them back to their own experiences. You may say something like...<br><br>*Notice the reactions you're having to other people's stories. Those reactions are important, and we* |

<table>
<tr>
<td>*What emotions did you feel in connecting with [Name's] memory?*<br><br>*What did your mind do in response to this experience? Were there thoughts you became attached to? Were you able to notice that you're the holder of these experiences rather than the experiences themselves?*<br><br>[To group member who shared the memory] *By noticing that you're the one that holds this memory…rather than the memory holding you…how does that change your ability to freely act?*<br><br>*How might you live the values that are present in this memory?*<br><br>*What if you can choose to do things that are about building these values into your life? Choice is possible, with these values as your guide.*<br><br>[Repeat for each group member, allowing them to share their experience of connecting to their own sweet spot and then allowing the other group members to share what it evoked for them.]</td>
<td>*want you just to observe them as a piece on your own board. You each have pain and you all have sweet spots as well. For now, we just want you to practice noticing your own reactions and hold them gently.*<br><br>If helpful, practice with particular clients noticing their reactions and thoughts in the moment as "pieces on their board" by saying something like…<br><br>*[Name], can you pause for a second and notice who's having that thought?*</td>
</tr>
<tr>
<td colspan="2">**Clinician Note:** In their language, clinicians should gradually move away from focus on a single morally injurious event and toward morally painful experiences more broadly. This helps to remind clients and clinicians that it was not a single event that is in control of the client's life. Rather, it was a pattern of behaviors in relation to a past event that have limited their functioning. Using the language of morally painful experiences and morally pleasant experiences de-emphasizes the significance of any one single event.</td>
</tr>
</table>

**Bridge to Skills Practice**

*You have all kinds of thoughts, emotions, urges and memories, some memories you may wish you didn't have (like your morally painful experiences), some are like this sweet spot we just explored. All these memories hold different "messages" for you… and given that none can be undone… the way forward is in choosing which messages you will act on… freely choosing… you can let the moral injury dictate how you live moving forward… or you can choose to let your values guide you…allowing these experiences to come with you… the choice is always yours. You're the one who carries all your memories, but with each step you take moving forward, you have a choice… remember, you're more than any memory, past action, thought, feeling, sensation… this is why choice becomes so important… it is freedom in action.*

*We'd like to share a metaphor that we think nicely represents what choice allows…*

*We can think about living a meaningful life like the process of creating a rich soup stock…it takes time…involves adding basic ingredients… things that may seem small and inconsequential…that in combination they are critical to building richer stock…building a richer life. Over the next few weeks, we would like you to continue practicing building a richer stock…continuing to make changes in your life by*

*doing things that are in the service of your values. Sometimes these steps may seem small or inconsequential. They aren't. They are critical to living meaningfully. You always have a choice to act on what's meaningful…to enrich the stock through awareness of your values and the choice to live them.*

**Skills Practice**
*To build a richer stock…a richer life…what will you do over the next week to live the values from your sweet spot memory? What small, deliberate steps will you take to connect to what matters to you? We'd like to hear from each of you what you'll do to live your values before the next session.* [Invite each client to share committed actions they plan to complete in the coming week.]

**Post-Session Clinician Review**
- Check-in internally with experience of session and reflect on personal or clients shifts that were noticed.
- Identify potential patterns of behavior observed to be aware of in future sessions.
- Compassionately identify any self-care needs that became evident during the session and, if willing, make plan to address them as soon as workably possible.

# SESSION 11

# CONNECTING TO THE PRESENT AS AN OBSERVER

## CLINICIAN ORIENTATION AND PREPARATION

### Purpose of Session

This session's purpose is to help clients connect with their capacity for choice. To achieve this purpose, clients are asked to identify a specific action they can choose to take soon and that they believe would contribute toward greater purpose and meaning in their lives. They then develop a series of concrete steps that will result in the completion of this action. During the session, clients are guided to connect the actions they've chosen to their personal values. Skills taught in earlier sessions, such as willingness to hold moral pain and the observer self, are emphasized by the clinicians as tools that clients can use to support their capacity to choose living in ways that honor their values in the moment.

### Materials Needed

- ACT-MI Workbook

### Pre-Session Clinician Preparation

- 1-minute mindful check-in with self (noticing own contact with the present vs. conceptualized pasts and futures, potentially including those about group members)

## SESSION CONTENT

### Review and Process Practice Exercise from Previous Session

*We'd like to first check in about experiences you've had in the last week where you were able to connect with things that felt meaningful or purposeful for you. They could big or small, pleasant or painful.*
[Invite group members to share examples of committed actions from previous week.]

### Bridge from Previous Sessions

*Thank you for all for sharing. For the past few weeks, we've been building awareness of and connecting with your observer self…the part of you that can see the morally injurious events you've experienced…the part who can see the painful memories you've had…the pleasant experiences you've encountered and all the moments in between without being defined by any of those events or memories. The whole purpose of building this awareness of an observer who can see your experiences but not be those experiences is to allow you the space to have your painful and pleasant experiences while also making the choice to live your values.*

*We'd like to do some work exploring choice today.*

### Experiential Exercise

- Conduct experiential and process elements
- Respond to challenges in comprehension or application of exercise
- Generalize skills to outside of session

### Introduction

*This exercise is similar in structure to some we've done before in that it has multiple parts. In the first part we'll ask you to imagine a meaningful action you can take in the next few weeks that's in the direction of your values. In the second part, we'll invite you to share your experiences with the group. When it's not your turn to share, just listen and notice what arises for you when you hear what your group members share. In the third part we will ask you to reconnect with your meaningful action and notice the series of choices you'll need to make as part of that act. Then we will return to the group and share our experiences.*

| Example Clinician Language | Notes for Clinician |
|---|---|
| **Experiential** | |
| *First, we'd invite you to imagine a meaningful action you would like to take in the next few weeks…something that matters to you…and something where you've noticed a barrier to action. This could be a very small or simple step, a larger more challenging action, or maybe action in an area where you haven't really acted yet—something outside of what's comfortable for you—but where there's the potential for real importance. What would you do to live your values meaningfully in the next few weeks?*<br><br>*Maybe it is having a meaningful conversation with a partner or family member, spending time being present with your children, having a difficult conversation at work you've been avoiding, getting more involved with your religious community, starting a healthier diet or exercise routine, picking back up a hobby that you had left behind, trying a new activity that you haven't tried before.*<br>*What are you <u>not</u> doing now that you wish you were doing?*<br><br>*We'd invite you to picture yourself doing whatever that thing is that matters to you. Immerse yourself in the sights, sounds and other parts of doing this activity, making room for any pleasant and painful experiences that might also arise.* [Pause]<br><br>*Now call to mind why this particular action is significant for you… Whatever behavior you've chosen to focus on, connect with the importance it carries for you... Why does it matter?... What about it gives value to life and to living?... Let yourself open up here and now to the meaning it holds for you…* [Pause] *If your mind tells you a reason you can't do the thing that matters to you…notice that you're the one having that thought and make the choice to bring yourself back to the meaning represented by this action you're focusing on. Make room within yourself for the experience of its meaning and the significance it carries for you in your world* [Pause]<br><br>*We invite you to reconnect with the group while staying in touch with this image you've just visited.* | Naming and committing to a values-related action in the presence of others can be a vulnerable and uncomfortable experience for group members. Be prepared to support them in this process by normalizing their experience and helping them name and hold their discomfort. |

| **Processing** | |
|---|---|
| *We'd ask you to share the meaningful action you imagined and why it's meaningful for you. If you're not sharing, just listen and notice your experience. See if you can notice what arises for you as you hear about their meaningful action…be an observer any emotions, memories, thoughts, sensations, urges that arise, but stay quiet until it's your time to share.* [Give each group member a few minutes to share.] | This initial processing should not become extended or overly detailed. There will be opportunity later in the exercise to expand and refine each client's meaningful actions. |
| **Experiential** | |
| *You're invited to close your eyes once again… Take a moment to reflect on what you heard from the others just now...letting yourself connect with what they shared… noticing what kind of qualities came with meaningful actions they described. Notice that each of the behaviors you heard holds importance for the group member who shared it.*<br><br>*Now you're invited to return to your own meaningful act that you would like to take in the near future… Imagine the setting again and connect to it as you did at the beginning of the exercise. Notice it fully in all its detail...*<br><br>*Slow down and now see that in this situation, like in every situation, you're given many choices. The choice to act, the choice to approach painful emotions, the choice to connect to what is meaningful inside of this action. You might also choose to avoid or ignore what matters to you, but we'd ask you to notice that even this is a choice. It's given to you to decide whether to take part in this thing you care about, and all that comes with it.*<br><br>*To fully participate in this meaningful act you've chosen, what pain might you need to make room for?... What demands or expectations might you have to let go?… Might there be stories about [trusting others/who you are/what you deserve] that you've grabbed on to?... That keep you from [being present with yourself or those you love]? What has it cost you and your loved ones to keep holding onto these narratives… How does holding onto them change your ability to act? Allow yourself to have any pain that arises as you imagine acting on what matters to you… hold the pain and observe it, without changing or controlling it. Instead, we invite you, in this moment, to just hold all these experiences as an observer, as pieces on your board, and see each of them as just a part of all that you have and are… And by choosing to hold them notice how you're free to live and move toward that which matters most to you.* | Clients may have learned stories that give themselves reasons for why they can't engage valued living. They may also experience anxiety or moral pain when confronted with the possibility of acting on their values in the immediate future. The clinician's role in the remaining parts of this exercise is to guide the client practice ACT-MI skills in such a way that they can clearly see how to move towards their meaningful act.<br><br>Bring to awareness for clients how choice is an inescapable aspect of the human experience. What is within their control, however, is how they will direct that capacity for choice.<br><br>Modify the examples in the exercise language to personalize it to the experiences of the clients. The more tailored the exercise, the more likely clients will be to connect with it and engage with to and imbue it with their own ideas, emotions and motivations. |

<table>
<tr><th colspan="2">Processing</th></tr>
<tr><td>We'll next ask you to share what specific choices you'll make to do this thing that is meaningful for you? What choices will you need to make related to your pain? What choices will you need to make related to your behavior? How might the tug of war show up for you…what will you need to open up to the pain that might come with this action?<br><br>[After each group member shares, invite the other group members to briefly share their experiences of the sharer's chosen action.]<br><br>Notice the reactions you're having to other people's stories. Those reactions are important, and we want you to notice them. We've seen that you all have your own pain and you all have your own sweet spots. You can also all live your own values as well.</td><td>Be mindful of how group members may be reacting to each other's chosen actions and, if needed, help them notice comparisons or self-critical thoughts bring them back to themselves as an observer of their own experiences.<br><br>Be ready to step in and take advantage of opportunities to reinforce and apply ACT-MI skills, such as giving up control, willingness, observer self, holding thoughts, etc.</td></tr>
</table>

**Bridge to Skills Practice**

*Thank you for joining us in that exercise. We want to highlight that you each have the ability to take steps toward what matters most to you, even in the presence of pain. That choice isn't something you make at one point in your life and then never revisit again. Instead, each moment of our lives is a choice about what we will choose to matter most to us.*

*Now we'd like to spend a little bit of time considering what choosing to live values could look like for you moving forward. As humans we experience an ebb and flow of awareness. Sometimes we find ourselves avoiding and have to choose to drop the rope. Sometimes we are living fully in the moment and immersed in experience. We want you to have all of these experiences and to also know that when you're stuck, you always have the choice to return to your values and to hold your pain as an observer. Connecting to your values might look small or inconsequential. We would invite you to consider a metaphor that we think nicely depicts the living of values.*

*We can think about living a meaningful life like the process of creating a rich soup stock…it takes time…involves adding basic ingredients… things that may seem small and inconsequential…that in combination they are critical to building richer stock…building a richer life. Over the remaining weeks in this group, we would like you to continue practicing building a richer stock…continuing to make changes in your life by doing things that are in the service of your values. Sometimes these steps may seem small or inconsequential. They aren't. They are critical to living meaningfully. You always have a choice to act on what's meaningful…to enrich the stock through awareness of your values and the choice to live them.*

*To build a richer stock…a richer life…what will you do over the next week to live the values? What small, deliberate steps will you take to connect to what matters to you? We'd like to hear from each of you what you will do to live your values over the next week. What choices might you need to make about your thoughts, emotions, urges to live this value? Connecting with moment to choose…pausing and then choosing.*

**Skills Practice**

*To help us practice holding and accepting that we can always contact our capacity for choice in the here and now, we'd ask each of you to complete another timeline exercise. In this second timeline exercise, you'll be asked to start at the present moment and look forward across potential moments of your own life that are yet to come. We'd ask you to reflect on what you will do to make this time meaningful… well spent… for you and your loved ones. We'd also ask you to identify future events (pleasant or painful) that you can reasonably foresee and imagine the kinds of choices you want to make that will help you show up in these moments in the way that serves what you care most about. When we come back together next week, we'll invite you to share what felt important to you as you answered these questions.*

**Post-Session Clinician Review**

- Check-in internally with experience of session and reflect on personal or clients shifts that were noticed.
- Identify potential patterns of behavior observed to be aware of in future sessions.
- Compassionately identify any self-care needs that became evident during the session and, if willing, make plan to address them as soon as workably possible.

# SESSION 12

# EXPLORING CHOICE IN THE PRESENT MOMENT

## CLINICIAN ORIENTATION AND PREPARATION

### Purpose of Session

This session's purpose is to practice connecting with the continuous observer self in relation to the living of values forward in time. In contrast to previous sessions which primarily focused on using the observer self to create flexibility around the past and the present, this session primarily focuses on using the observer self to create flexibility around the client's relationship with their own future. The continuous nature of the observer self is highlighted to emphasize that the client's observer self has been present across time and will continue to be present and available for the client to help hold their experiences, including future moral pain. From a perspective of openness and possibility created by connection to the observer self, clients are invited to consider future committed actions that would contribute to a lifetime of meaning and purpose.

### Materials needed

- ACT-MI Workbook

### Pre-Session Clinician Preparation

- 1-minute mindful check-in with self (noticing own contact with the present vs. conceptualized pasts and futures, potentially including those about group members)

## SESSION CONTENT

### Bridge from Previous Sessions

Given the strong connections that can form between group members and with clinicians, the Clinicians take a few minutes at the beginning of this session to discuss the remaining sessions of ACT-MI

*As we start today, we want to recognize that we are approaching the last few sessions of our group and provide a roadmap of what our remaining meetings will hold. Our next session will be a review of all the skills we've covered so far in group and is meant to help us remember and integrate these parts together. After that will be our final group session which will focus on the things each of us have taken away from our time together and sharing appreciations for the ways we've supported each other along the way. Finally, we will have our last individual sessions where we'll meet with you one on one to discuss the progress you've made in group and what your next steps might be on your journey.*

### Review and Process Practice Exercise from Previous Session

- Clinicians elicit valued actions taken by group members since the previous session, emphasizing clients' small, simple, and significant steps towards what matters to them.

### Introduce New Content

*In our recent sessions we've been talking about how your observer-self exists across time. Your observer self was there when you were born, it was there watching as you grew up. It's been there observing all your pleasant and painful experiences across time, including your morally injurious events. Today we're*

*going to stand here in the present but gaze into the future. Our goal for doing this isn't to perfectly predict what's going to happen since none of us can know that. Instead, the goal is to get in touch with how you want to live in the time you have left.*

*To help us do this get out the Timeline – Part II page in your workbooks from last week. If you weren't able to get to the practice exercise, we understand. Just turn to it. If you don't have the Timeline – Part II page with you, please get a piece of paper and a pen take a moment to recreate it.* [Provide a few moments and guidance to recreate the Timeline – Part II page from the workbook]

**Experiential Exercise**

- Conduct experiential and process elements
- Respond to challenges in comprehension or application of exercise
- Generalize skills to outside of session

**Introduction**

*We'll do an exercise now that includes us closing our eyes and imagining something and then opening our eyes and sharing what we pictured. We'll do this a couple of times.*

| Example Clinician Language | Notes for Clinician |
|---|---|
| **Experiential** | |
| *Take a look at the second timeline page… this one starting here and now in this moment and extending forward, eventually to the time when this life ends for you. Look at the page and imagine all the experiences, big and small that will someday dot its surface… And notice how the page is open to them all. It's not trying to fold up against the painful ones or cling onto the pleasant ones. See if you can be like the page in this moment, acknowledging and open to all that was, all that is, and all that is yet to come.*<br><br>*If you could imagine a version of yourself more fully living your values in the future, what would they be doing?... How would they be moving in the world?... How would other people see them?... What are you <u>not</u> doing now that you wish you <u>were</u> doing? ... Gazing ahead into the future, imagine what it would look like if you were taking those steps… Fully immerse yourself in the movements, the sights, the sounds of that future moment you want to bring into being.* | Clients will often experience a sense of anxiety or being overwhelmed at considering the remaining time left in their lives. If clients are older, they may reflect on how their remaining time is short compared to the amount of life they have already lived. The goal of this exercise is to help the client identify and begin moving towards valued actions they can take in their future, regardless of their remaining time. |
| **Processing** | |
| *We'd like to give each member of the group some time and space to share what they imagined themselves doing. We'll ask each of you to describe what you imagined to help us get a sense of what this valued action would look like. [Name] would you mind going first?* | As this is one of the last opportunities they will have in the group to focus for an extended time on identifying committed actions, clinicians should feel more free to gently push clients to articulate specific details about what their |

| | |
|---|---|
| [Each client shares in turn and the clinician's ask follow-up questions to help expand and refine each client's responses as necessary.]<br><br>*What about this is meaningful or important to you?*<br><br>*What's gotten in the way in the past?*<br><br>*If I were a fly on the wall, what would I see you doing?*<br><br>*Was there a time when you saw someone showing this quality? What did they do to show this value?*<br><br>*What's hard about picturing this? Where do you find yourself getting stuck?*<br><br>[If needed] *One example you might consider would be [example]. What do you notice as we share that idea? Would it lead you closer to your values or would you want to change it in some way?* | valued activity will entail and what actions will likely be required of them to move towards it.<br><br>It is very common even this late in the group's sessions for clients to struggle to identify concrete steps they can take to make to move towards their values. It can be helpful to normalize this as one of the skills that clients have come to session to learn. Clinician's should encourage clients to generate their own answers but if necessary, can offer suggestions to clients so they have content to continue the exercise with. |
| **Experiential** | |
| *[Return to eyes closed] We'll start by noticing where you are on the timeline… Stretching behind you is the path where you weren't living these values as fully, and laying out in front of you is the path continuing on, extending forward into the future. Notice how time is always flowing… presenting you with constant opportunities to change how you live... to shift…. to show up in a new way… Now look ahead to this valued action you imagined earlier… Connect again to why it matters… why it's meaningful… Keeping this valued action in your view, what choices do you need to start making now to point yourself toward that future where you are [Insert valued actions from first part of exercise]. What steps could you take today to move in that direction?…* | The experiential exercise here connects the client's stated goal in the more distant future to actions in the present that they can begin taking immediately to begin aligning their lives more closely with their chosen values. |
| **Processing** | |
| *We'd like to give each of you some time to share again.*<br><br>*[Name], what choices will you need to make now to start or continue moving in the direction of that change? Before the sun sets on today, what is something you can do?*<br><br>*What kinds of difficult feelings and thoughts are likely to come up based on your past?* | Clients may continue to focus on large or dramatic actions. These can be necessary at times, but should not come at the exclusion of smaller, more consistent acts.<br><br>Small Acts: Saying "I love you" to family members, make eye contact when talking, let kids choose an activity to do together |

| | |
|---|---|
| *What moves can you make in that moment to stay connected to what you care about?*<br><br>[As indicated, review and apply ACT-MI skills taught previously to help clients relate flexibly to their futures and the valued actions they're considering.] | Large Acts: Moving across the country, attending a funeral, taking on a new job |

**Bridge to Practice Assignment**
*Thank you for sharing with us. Our hope is that this exercise, and all the sessions of this group, have helped you see the possibilities available to you going forward. We also want us all to be aware that there's one common event that we'll all face, and that is that our time in this life is limited. Your time is precious, and that's why we're having this discussion now, so you can choose how you live and what you leave for the people who matter to you and the world.*

**Skills Practice**
*For your practice before our next session, we'd invite you to begin taking physical steps toward the future you each shared with us today. That may include some of the concrete steps we talked about, or it may include others we haven't shared. What's important is that you each begin that towards this future by choosing to engage in the behaviors that will eventually lead you there.*

**Post-Session Clinician Review**
- Check-in internally with experience of session and reflect on personal or clients shifts that were noticed.
- Identify potential patterns of behavior observed to be aware of in future sessions.
- Compassionately identify any self-care needs that became evident during the session and, if willing, make plan to address them as soon as workably possible.

# SESSION 13

# CONNECTING WITH THE ARC OF THE GROUP

## CLINICIAN ORIENTATION AND PREPARATION

### Purpose of Session

This session's first purpose is to review and reinforce the concepts and skills taught from across previous sessions. By moving through the major concepts and skills taught in ACT-MI, clients are given the opportunity to better integrate these separate elements into an interlocking and over-arching process of psychological flexibility. This review also provides clients an opportunity to explore the changes they have made over the course of ACT-MI and contrast their current functioning with the patterns of moral injury they had engaged in before starting group. The second purpose of the session is to help clients experience compassion for themselves by first eliciting compassion for a hypothetical person experiencing moral injury and then offering to themselves similar responses.

### Materials Needed

- ACT-MI Workbook

### Pre-Session Clinician Preparation

- 1-minute mindful check-in with self (noticing own contact with the present vs. conceptualized pasts and futures, potentially including those about group members)

## SESSION CONTENT

### Bridge from Previous Sessions

*This is our second to last group session. Next week we'll spend some time taking stock of where we've come as a group and what we've learned from one another. In two weeks, we'll have an individual session with each of to review the changes that you feel you've made over to course of group and discuss what you thinks comes next when it comes to living your values.*

*We realize that your journeys with moral pain didn't start with this group and that they won't end with this group either. Our work here has been to help teach a new way of relating to your moral pain so that it doesn't pull you away from living a meaningful life going forward. For today, we're going to look back across the key concepts we've been covering and what you've taken away from practicing those skills.*

### Review and Process Practice Exercise from Previous Session

At the start of this session, Clinicians do not typically check on the group members' valued actions from the previous week. Rather this session focuses on a review of the entire arc of the therapy up until this point in the group. If clinically indicated, clinicians may find opportunities

during the review to reinforce or deepen the work of previous sessions identifying concrete steps clients can take in the direction of their values.

| Example Clinician Language | Notes for Clinicians |
|---|---|
| **Clinician Note**: As the clinician's conduct this review and process the different components of the exercise, ensure that all group members are being given opportunities to comment and share. This both emphasizes their values as members of the group's community but also provides clinician's a chance to assess their comprehension of ACT-MI skills and concepts. | |
| **Experiential** | |
| *We started this group by talking about values…and why you made the choice to participate in this group in the first place. Remember the values sort exercise you did to better understand how you want to show up to different areas of your life. Our goal was to start putting these values into action, by building up small patterns of behavior, bit by bit, across our time together. See if you can remember the values you chose and what it was like to name them for yourself…* | Note that over the course of ACT-MI, the client may have evolved in what they value and how they prioritize them in different contexts. The goal is not to find the "true" or "correct' values, but help the client become more skilled at selecting and moving in the direction of values. |
| **Processing** | |
| *Doing this group was itself an important choice. What was important enough to you about this that you said 'yes' to doing this group to begin with?*<br><br>*What feels like an important direction for you <u>now</u>? What do you notice yourself caring about the most?*<br><br>*What has helped you connect with what matters to you? How can you continue to do that the future?* | If needed, remind clients that values are more like a compass than a destination. We never arrive but instead continue in a process of ongoing reflection and adjustment to continue moving in the direction of what matters to us. |
| **Experiential** | |
| *After exploring our values, we reflected together on all the possible strategies that you've used to avoid and control moral pain. We learned about the things you've tried across your life to avoid the moral pain—[alcohol, drugs, suicidal ideation, isolating, spending time less vulnerable emotions overworking]…the [number] total years of suffering. We built on this with the tug of war, where you connected with the struggle of trying to fight, avoid, control your pain…we introduced dropping the rope as a strategy that could free you to live your values if you make room for the pain that could come with it.* | If possible, display an image of the creative hopelessness exercise from session 2 to help clients recall the lists of morally injurious behaviors they had created and evoke a sense of contrast compared to their current experience. |

| **Processing** | |
|---|---|
| *What feelings or thoughts come up as you look back at that list we created, and all the years spent stuck?*<br><br>*What's happened to those urges, thoughts and emotions that make up the moral pain? Are they completely gone forever?*<br><br>*What's changed in your <u>relationship</u> with moral pain over the course of group?* | Listen for a greater sense of freedom and purpose, even if painful experiences have not left.<br><br>Consider noting that in ending their morally injurious behaviors, clients' total amount of suffering may have decreased, even as they have opened themselves to their pain. |
| **Experiential** | |
| *After we noticed that avoiding the pain wasn't very effective, we tried instead to better understand it….to literally connect with its value for each of you. We worked together to see that the pain and your values are really two sides of the same coin. Moral pain often shows up because we care about other people. We feel hurt because we care, and if we care about something we also open ourselves up to feel hurt related to it.* | To heighten clients' connections to the two-sided paper exercise from previous sessions, consider explicitly naming the value-pain labels clients previously articulated. |
| **Processing** | |
| *What will you want to remember about your pain when your mind tries to convince you to pick up the rope and start fighting it?*<br><br>*What does it mean for you that your pain and values are connected?*<br><br>*Practicing acceptance that our minds will want to keep fighting…and we will keep grabbing, but noticing when and choosing to hold pain and move toward values is key.* | Emphasize for clients that acceptance is a continuous skill. In other words, dropping the rope/letting go of the fight to control moral pain is a choice that must continually be made.<br><br>Connect this choice to what matters to the client so they can access the motivational quality of their personal values. |
| **Experiential** | |
| *We learned that getting stuck in the fight usually happens when we've gotten attached to or hooked by a particular thought, emotion or experience. To help us practice getting unstuck, we used the metaphor of a chessboard to help distinguish our experiences, which are like chess pieces, from our observer selves, which is like the board that holds the pieces. We saw that our observer selves hold and contact all your experiences…without becoming them.* | |

<table>
<tr><td colspan="2">Processing</td></tr>
<tr><td>How has the way you understand yourself changed over the course of the group?<br><br>What part does the moral injury now play in that understanding of yourself, if at all?<br><br>The chess board… You… hold it all…and continually expand as you experience more and more over the course of your life.</td><td>As in previous sessions, be quick to help clients practice noticing any thoughts or experiences that they seem to be getting stuck in by asking questions like, “[Name] is that a piece? Who’s aware of that thought? Can you just notice that for moment?”</td></tr>
<tr><td colspan="2">Experiential</td></tr>
<tr><td>The second half of group has been about applying these concepts and skills to our lives. That includes the really important and powerful memories we have, like the sweet spots we visited as well as the painful experiences we’ve worked to hold, like the morally injurious events. Because time keeps moving and choice keeps presenting itself to us, we also connected with our ability to choose in the present moment. That awareness of choice allows us all to takes steps here and now that will point us in the direction of how we want to live going forward.</td><td>As needed, highlight that no one can fully control what thoughts, emotions or other internal experiences show up in their awareness. What we can control is how we choose to relate to it while it’s there.</td></tr>
<tr><td colspan="2">Processing</td></tr>
<tr><td>What’s next for you and those you care about?<br><br>What steps will you take to connect to what you care about and build a richer life?</td><td>Review importance of small, repeated actions that build over time (e.g., Soup Stock metaphor)<br><br>Identify committed actions from each member</td></tr>
</table>

**Experiential Exercise**
- Conduct experiential and process elements
- Respond to challenges in comprehension or application of exercise
- Generalize skills to outside of session

**Bridge to New Content**
*To help us connect more with all these skills, we’d like to invite you to do an exercise. We’re going to tell you about a person, and we want you to start imagining them. While you do, pay attention to how you’re feeling towards them.*

| Example Clinician Language | Notes for Clinician |
|---|---|
| **Experiential** | |
| *(Eyes closed, ground self, settle into the moment). This person is an adult about your age. They came from a family that wasn't perfect, and that still isn't perfect. They joined the military (maybe even your same branch) and like you had many experiences and developed many relationships during their time serving. Some experiences were positive, and some were very, very painful. Like you they experienced a moral injury…. and in the years since they got out of the military, they've not known how to deal with it. They might wonder if they even deserve to recover. They're not sure they can trust others again. They often get stuck in a cycle of trying to forget and not being able to let go of what happened. In trying to escape or atone for the pain, they've made choices that have led them farther away from the people and things they care about. A Clinician at the VA has told them about a group that exists for Veteran's struggling with these kinds of moral injuries, but this person's not sure.* | The hypothetical person is intended to possess enough similarity to the group members that they are able to identify with their experience, while still be general enough for the client's to reasonably imagine them as a separate individual. Clinicians can modify the characteristics of the hypothetical person if needed to serve this purpose. |
| **Processing** | |
| (Eye's open) *What parts of their story do you connect with?*<br><br>*What do you feel for this person? What is happening right now as you imagine them?*<br><br>*What do you hope for them? What possibilities do you see as being open for them?* | Guide clients in identifying the goodwill and openness that the experience this hypothetical person. |
| **Experiential** | |
| (Eyes closed again) *Reconnect with this person you've been imagining. See them again. Reflect on how well you understand them, and what they're going through. In fact, as you look at them, you realize that the reason you understand them so well is because that this person <u>IS</u> you. Their experiences <u>are</u> your experiences… their life <u>is</u> your life…*<br><br>*…And allow yourself to open up to the goodwill and compassion that you've felt for this person… Hold yourself, and all your fears and pain and joy, with the* | Proceed slowly during this portion of the exercise. Client's may fear granting themselves this degree of self-acceptance and so benefit from time to gently and gradually approach themselves with compassion.<br><br>Within ACT-MI, compassion is not an emotion. Rather, compassion is a behavior… a |

| | |
|---|---|
| *same understanding, gentleness and acceptance that you held them with... Not criticizing, not condoning, but instead supporting… hoping… encouraging yourself forward on your own path and all the possibilities that lay before you…* | choice… to be present with ongoing suffering (from the Latin, meaning "to suffer with"). In this sense, this exercise represents a culmination of all the skills taught over the entire arc of ACT-MI. |
| **Processing** | |
| (Eye's open) *What would be different in your life if you could hold your own experiences going forward in the same way you held this other person and their experiences?*<br><br>*How might it free you to more fully connect with those you care about?*<br><br>*What would it look like to do this? How would others know it was happening?* | Pivot into discussing committed actions. Invite clients to be specific about the types and components of the actions they could take in the direction of their values. The goal in doing so it to help clients translate abstract statements about values into clear and concrete behaviors they can intentionally perform. |

**Bridge to Skills Practice**
*Thank you for joining us in that exercise. Our hope is that you can use your experience with it to more fully appreciate that your experiences also matter and are worthy of your own time and attention. Practicing this in our session today also provides an example of what we would like you to practice this coming week.*

**Skills Practice**
*We invite you this week to practice compassion for yourself and others, not as a way to avoid or change what you or others are feeling, but instead to show up to those feelings and honor them for the important experiences that they are… to make room for them… to acknowledge that they matter… that the person having them matters. Use the skills we've learned in this group to help you do this – being present, dropping the rope, seeing from the observer self, and remembering what matters most to you in your life and taking concrete steps to move in those directions*.

**Post-Session Clinician Review**

- Check-in internally with experience of session and reflect on personal or clients shifts that were noticed.
- Identify potential patterns of behavior observed to be aware of in future sessions.
- Compassionately identify any self-care needs that became evident during the session and, if willing, make plan to address them as soon as workably possible.

# SESSION 14

# HONORING OURSELVES AND EACH OTHER

## CLINICIAN ORIENTATION AND PREPARATION

### Purpose of Session

This purpose of this session is to connect group members with a sense of experimentation and possibility with their values and committed actions. An additional purpose of this session is to help group member's practice connecting with pleasant moral emotions.

### Materials Needed

- ACT-MI Workbook

### Pre-Session Clinician Preparation

- 1-minute mindful check-in with self (noticing own contact with the present vs. conceptualized pasts and futures, potentially including those about group members)

## SESSION CONTENT

### Review Final Session Agenda

*This is our final session as a group. After this, each of you will have your individual sessions where we'll discuss what your next steps will be in your recovery from moral injury.*

*Before we begin, we'd like to take care of some items of business:*

Discuss the following as needed:

- Processes for contacting group Clinicians
- Guidelines for group members exchanging contact information
- Coordinating future care with referring Clinicians
- ACT resources for additional practice

### Bridge from Previous Sessions and Review and Process Practice Exercise

*Last week we talked about what it might look like to support and care for yourself, as well as caring for others. We'd like to hear if you took any steps this week to support yourself but first, we'll do an exercise to get back in touch with why this might matter.*

| Example Clinician Language | Notes for Clinician |
|---|---|
| **Experiential** | |
| *We'd invite you to close your eyes and to start by noticing some of the people that you carry with you, in your mind and in your heart. They might be family or friends. People from your past or your present. We'd invite you to spend a moment calling to mind images of these people… noticing their faces… noticing the connections you have to all of them… the ways they have mattered and continue to matter in your life… see* | The majority of this session will focus on supporting group members in exchanging gratitude for one another and the changes they have made over the course over ACT-MI. This initial exercise is an opportunity for group members to have guided |

| | |
|---|---|
| *them fully… Allow them the space to have all their dreams and hopes…all their fears and shame… all their experiences exactly as they are. In seeing their hurt, you might sense your own pain… your own urge to pull back or struggle against… just notice it… holding this urge or any other experience you observe without acting on it.*<br><br>*Knowing that you can't fix or take away their feelings, instead we would invite you to offer them the gift of your presence... your willingness to be present… with them… and share in their moments… even the painful ones… This kind of presence…compassion isn't a feeling. It's an action… a choice you make to stay with pain… We invite you to choose now to stay with them, even in their pain and hurt…*<br><br>*And while you're here with them… we invite you to also choose to hold your own experiences in that moment too. Expanding to make room for your dreams and hopes, your fears and shame, all of your experiences to be, just as they are… Allowing these experiences that you hold within you… just to be… This kind of compassion isn't a feeling. It's a choice we make to stay with pain… including our own.* | practice choosing offer compassion towards themselves and others. |
| **Processing** | |
| (*Eyes open) What did you notice about making room for your pain? Did your mind tell you anything about this?*<br><br>*What ways were you able to engage in self-compassion since our last session?*<br><br>*Going forward, what are ways you can choose to practice self-compassion in your life? (How would you know you were practicing this?)* | Use this processing section to explore clients' willingness to behave compassionately.<br><br>As in previous sessions, help clients articulate the kinds of specific actions they could take to show compassion towards themselves and others. |

**Introduce New Content**
*You've each just shared some of the individual changes you've each been making in your lives over the course of this group, and of course, that's the point. But we've also been on this journey together. Moral injury can be as much about being disconnected from others as it can be about being disconnected from yourself. We do this intervention not only to practice connecting with painful emotions (like guilt shame, contempt, and anger) but to also connect with pleasant moral emotions like gratitude, caring, pride and inspiration. We  have this treatment in a group because the values that are often affected by moral injury, have to do with being in a community… and we've really built a community here together. There have been moments that we've been inspired by way each of you has connected with the people in your own life and with people in this group.  We think it's important to spend some time acknowledging and honoring the community we've built together and the positive impacts we've had on each other over the past few months.*

**Experiential Exercise**

- Conduct experiential and process elements
- Respond to challenges in comprehension or application of exercise
- Generalize skills to outside of session

**Introduction**

*Sometimes parts of our experience get lost when we are struggling against pain. Usually, in avoiding some emotions, we end up blocking others too, including the pleasant ones. That often means that in suppressing our pain, we also suppress our joy. We've been focused on not just learning how to hold pain, but on opening up to and contacting this joy too. We're going to use the work we've done in this group together to help us connect to the pleasant moral emotions of, gratitude, pride, caring, awe and inspiration.*

*To do this we're going to ask you to connect to a time when you felt these pleasant moral emotions sometime in the past as well as sometime during this group. Like we've done before we'll go back and forth between being in the memories and sharing with each other. We'll guide you along the way.*

| Example Clinician Language | Notes for Clinician |
|---|---|
| **Experiential** | |
| *(Eyes closed) We'd invite you to look across your entire life thus far observing all of it across time... See yourself as a child, as a teen, as an adult… As we've already discussed before, there have been many different experiences you've had, some extremely painful, and some extremely pleasant. As you survey the range of experiences you've had across your life, we invite you go back to a time before this group when you felt really grateful, proud, caring or inspired…*<br><br>*When you have it, signal us with your hand* [Wait for all group members]<br><br>*Now that you all are in touch with this moment… we'll take a little more time to let you connect with it. Really bring yourself into that moment in time… noticing what you felt then…. what it meant to you…. why it was important to you…(Eyes open)* | The point of the initial portion of this experiential exercise is to help the clients access pleasant moral emotions so that when they are asked in later portions to connect to values-consistent memories from the group, it will be easier for them to recognize and discuss them. |
| **Processing** | |
| *We'll now briefly share with each other. We won't go into much detail because there's a second part to this exercise, we want to tie us back into.*<br><br>*In a sentence or two, what was the event? And in a few more sentences, why it was important to you?* [Give each group member a chance to share] | If needed, gently redirect clients to ensure that they do not drift too far into storytelling. For this part of the exercise, only a brief description of the event and its significance for them is necessary. |
| **Experiential** | |

| | |
|---|---|
| *(Eyes closed). We now invite you to scroll forward from this event in the past up to the time we've been working together in this group. As you did with your whole life up to this point, scan over and observe the journey of the group together… Notice what it has meant to you… what each of the members of this group have meant to you… and how they have been a part of your journey with your moral pain… During this group, when have you noticed yourself prompted to feel inspired?… to feel caring?… to feel proud?… to feel grateful?… maybe it was from another group members actions or maybe in response to your own behavior… As we did before, choose a moment where one of these emotions was present for you and let us know with your hand when you have it.*<br><br>*Now bring yourself back to that moment from this group… What was said or what was done that made an impact on you?… Why did it matter to you?... What about that do you want to carry forward from this group into other parts of your life?...* | Asking clients to explore and acknowledge events from the group or group members that have prompted pleasant moral emotions is likely to be more evocative and so provides an opportunity to more fully practice ACT-MI skills such as contact with the present moment, willingness and committed actions.<br><br>Before or during this exercise, clinicians should identify instances from group that led them to experience pleasant moral emotions as well and be prepared to share them if time permits. |
| **Processing** | |
| *(Eyes open) What was the moment you chose from this group that was meaningful for you?*<br><br>*What about it was meaningful to you?*<br><br>*How do you want to bring those valued qualities into other parts of your life? What will you do to make this happen?* | Be careful to manage time so that every group member can share. If possible, allow other group members to respond to what their group members have shared. Once all have gone, clinician's should also share their responses to these questions. |

**Concluding Comments**
*This group has come to an end, but that doesn't mean the journeys or the relationships are over. The positive impacts we've made one each other will go on as we each move forward in our lives. We're grateful to have been a part of each of your recoveries and wish everyone the best.*

*The last session of ACT-MI will be a final individual check-in session [next week]. The purpose of these final 1-on-1 sessions is to review some of the patterns you've gotten stuck in and how to intervene in these patterns moving forward based on what you you've learned from the group. We're excited to meet with each of you.*

The clinicians then adjourn group.

**Post-Session Clinician Review**
- Check-in internally with experience of session and reflect on personal or clients shifts that were noticed.

- Reflect on your and this group's experience and try to identify lessons that may be learned to enable you to more effectively provide ACT-MI in the future.
- Compassionately identify any self-care needs that became evident during the session and, if willing, make plan to address them as soon as workably possible.

## SESSION 15

## CASE CONCEPTUALIZATION AND FUNCTIONAL ANALYSIS

**Purpose of Session**
The purpose of this session is to further consolidate and generalize the concepts and skills taught across previous sessions and help the client identify what behaviors and goals they would like to continue practicing to support their ongoing recovery from moral injury. To accomplish this, the client is guided in creating a third chain analysis with a clinician that reviews a recent situation that was relevant to their pattern of morally injurious behavior and apply ACT-MI skills that apply to this context. As part of the session, the clinician directs the clients attention to consider what future steps are indicated to support their ongoing recovery from moral injury (i.e. additional goals, unaddressed values, referrals for additional services).

As with the chain analyses in sessions 1 and 7, the content of these individual case conceptualization sessions can be used to help focus and tailor discussions on the factors most salient to each group member's moral injuries in subsequent group session.

**Materials Needed**
- ACT-MI Chain Analysis Worksheets

**Pre-Session Clinician Preparation**
- 1-minute mindful check-in with self (noticing own contact with the present vs. conceptualized pasts and futures, potentially including those about group members)

**SESSION CONTENT**

**Review Case Conceptualization/Functional Analysis**
*This meeting's purpose is similar to the prior individual meetings you attended as part of ACT-MI. Like those sessions, we'll go through an example of a recent time that moral injury showed up in your life and better understand what triggered your behavior and what is keeping that pattern alive. We'll also spend some time exploring ways we can break that pattern by using the skills we've learned so far in group and talk about what comes next for you on your path forward.*

| Example Clinician Language | Notes for Clinician |
|---|---|
| **Clinician Note:** Although chain analysis can be a clarifying and valuable tool for helping clients understand their behaviors, it is important to also track the client's emotional experience of the exercise. For example, some clients may find chain analysis to be a validating experience in that it helps them see that their suffering stems from understandable, if unhelpful, motivations to escape pain. Yet other clients may experience the chain analysis as emotionally evocative as they contact emotions and memories they otherwise attempting to avoid. | |
| **Identify target behavior** | |
| *Tell me about a recent behavior of yours that you'd like to understand better. For example, it could be a behavior that* | |

<table>
<tr><td>you'd like to change so you can better do what matters to you or it can be a time you lived your values.<br><br>What kicked this chain of events off for you…what triggered that behavior this time?<br><br>Did anything make it more likely you would respond to [prompting event] in the way you did? (e.g., prior alcohol use, poor night's sleep)</td><td></td></tr>
<tr><td colspan="2">Fill in steps between target behavior and prompting event</td></tr>
<tr><td>How did you get from here (prompting event) to here (target behavior)?<br><br>[For each link in the chain] What happened next [X]?</td><td></td></tr>
<tr><td colspan="2">Clinician Note: The chain/functional analysis is meant to tell a story of the reinforcer(s) maintaining the Veteran's moral injury. If the exercise starts to feel confusing or scattered, bring yourself back to the purpose of the exercise—to learn which behaviors are providing them relief or reward, and in so doing keeping their moral injury alive. The other purpose is to understand what the costs of these behaviors have been for the client.</td></tr>
<tr><td colspan="2">Introduce Short-Term Consequences (Why we keep doing what we do)</td></tr>
<tr><td>As we've shared before, people keep doing these behaviors because of the effect it has on us or our surroundings. If a behavior reduces something we don't like to feel, we keep doing it. If we are anxious and feel relief by isolating, for instance. If it increases our contact with something that feels good, we keep doing it. If you feel anger and you drink, and you feel more lose, for instance. It makes sense. Feeling better drives a lot of behavior. It's important that we know how this behavior impacts you and your environment—including other people around you—in the long run, so that we can identify strategies and tools you can use to give you more choice about what you do in response to moral pain.</td><td>Morally injurious behaviors, by definition, lead clients to depart from values or cause themselves or others added suffering.<br><br>Providing education about how short-term consequences give immediate reinforcement can help give clarity about to why the client has engaged in these behaviors that otherwise appear costly and destructive.</td></tr>
<tr><td colspan="2">Internal Short-Term Consequences</td></tr>
<tr><td>What did you notice immediately happened to your experience <u>during or after</u> <u>[target behavior]</u>?<br><br><u>What happened to</u> your emotions…Your thoughts…Your sensations…Your urges…your memories during and/or immediately after engaging in <u>[target behavior]</u>?<br><br>Did <u>anything shift</u> about the intensity of these experiences?</td><td></td></tr>
</table>

| | |
|---|---|
| *What did you notice about how this behavior affected you in the moment?*<br><br>*Did this behavior take away or reduce anything you didn't want to feel? (Did it give* <u>*relief*</u> *from something painful? Turn down the volume on the pain?)*<br><br>*Did it increase your ability to feel something you would rather feel instead? (Did it lead to something enjoyable?)* | <br><br>Examples: avoiding guilt and shame, reduced anger, distracted from painful memory<br><br>Examples: sense power/control, connection to people who have passed away, vindication |
| **Long-term Consequences** | |
| *How does* [target behavior] *affect your relationships, spirituality, mental health, physical health, work?*<br><br>*What are the long-term impacts of* [target behavior] *on you? …On those you care about?* | Probe how the target behavior affects a range of functional domains (social relationships, employment, physical health, finances, religion/spirituality) |
| **Breaking Pattern of Moral Injury** | |
| *Now let's look over these links in the chain to see if we can find places where some of the skills and tools we've been learning might have been helpful. What do you notice?*<br><br>*Where might it have been helpful to slow down?*<br><br>*Was there a thought you might have gotten hooked on or some pain you were pushing away?*<br><br>*How could you have gotten in touch with your Observer-Self or with your values?*<br><br>*What physical action could you have taken that might have demonstrated what you care about?* | Guide the client in identifying where ACT-MI skills might have helped interrupt morally injurious patterns of behavior. As needed, make suggestions to the client,<br><br>Even small actions, particularly early on in the chain's sequence of events, can help clients change the outcome of the situation. |
| **End Chain Analysis** | |
| *Thanks for working with me to better understand how these patterns of moral injury show up in your life. I want to check in with you and see what you've take away from this session.*<br><br>*What stands out to you?* | Clinician listens, validates, and responds supportively |

**Next steps**

- Review with client what they see as their next steps in recovery from moral injury. Continue to connect potential steps back to their primary values and help the client identify what concrete actions they can take to move in these directions.

- If appropriate, discuss potential referral options for additional services with the client and engage in shared decision-making about what would best serve their treatment goals.

**Closing of session**

- Thank client for their involvement in the group and review contact information and procedures in case client has additional questions or concerns.

**Post-Session Clinician Review**

- Check-in internally with experience of session and reflect on personal or clients shifts that were noticed.
- Identify potential patterns of behavior observed to be aware of in future sessions.
- Compassionately identify any self-care needs that became evident during the session and, if willing, make plan to address them as soon as workably possible.

**Chain Analysis Common Challenges and Possible Clinician Responses**

| Target Behavior Challenges | Possible Clinician Response |
|---|---|
| Client gives a broad, generalized target behavior (e.g., drinking) | Elicit further specific details (e.g., Tell me a time when you were drinking. Where were you? How much?) |
| Client identifies another person's behavior | Validate the event and invite them to share what they did in the situation that might be keeping them stuck. |
| Client gives a covert behavior (mental actions) | Clinician may need to provide more labeling (i.e., labeling the behavior 'rumination') |
| Client gives an example of NOT doing a valued behavior | Help the client identify what they were doing instead of the valued behavior |
| **Prompting Event Challenges** | **Possible Clinician Response** |
| Client has trouble identifying what the prompting event is because it happened so quickly | Work backwards from target behavior to get the full episode of the behavior ("*What happened right before*…") |
| Client has trouble identifying what the prompting event is because it feels so routine | Explore if there was something distinct about the timeframe/circumstances of this particular episode (Tell me what was unusual about this particular day)<br><br>Explore events/situations that may serve as vulnerability factors (e.g., anniversaries, alcohol use) |
| **Link Challenges** | **Possible Clinician Response** |
| Clinician or client feels unclear about the timeframe of the chain | Identify the time span between the prompting event and target behavior happened (e.g., two hours) |
| Chain feels unclear or doesn't tell the story of the behavior | Be direct and curious with the client about this and invite the client to collaborate with you in helping to understand potential missing links |
| Client experiences shame and self-critical thoughts in response to chain analysis | Use frequent validation techniques such as, validating their experience, normalize the experience as a product of their prior experiences. Eventually validate by recognizing short-term consequences that reinforce the behavior. |
| Clinician experiences discomfort in reaction to client's moral pain | Use ACT-consistent skills to lean into and validate both the client's and clinicians own experience |
| **Short-term Consequence Challenges** | **Possible Clinician Response** |
| Client is focused on long-term consequences of the target behavior and so can't validate that their own experience makes sense. | Reorient the client to the immediate consequences of the target behavior (e.g., during the target behavior or seconds or minutes after). "*What happens in that moment as you are* [insert target behavior]?" |
| Client doesn't report their target behavior reduces painful emotions and so can't be reinforcing in the short-term. | Explore whether the target behavior is ***positively*** reinforcing, such as increasing a sense of control or by creating a more preferred emotion (e.g., feeling guilt rather than shame). |
| **Long-term Consequence Challenges** | **Possible Clinician Response** |
| Client doesn't report their target behavior has long-term negative consequences for them. | With curiosity and openness, inquire what the costs of this behavior have been in the past. If the client cannot identify any costs, explore further what has brought them into treatment and see if any long-term consequences of |

| | |
|---|---|
| | morally injurious behaviors can be identified. If none can be identified, consider checking with the client's referral source and clarifying purpose of ACT-MI as needed. |
